# Supplementary material for: Tenascin-C orchestrates radiotherapy-induced head and neck tumor regression
Source: EMBO Mol Med. 2026 Mar 31;18(5):1707–43. doi: 10.1038/s44321-026-00406-8 (PMC13179387; doi:10.1038/s44321-026-00406-8)
Supplement: Supplementary file 1 — Appendix [file 44321_2026_406_MOESM1_ESM.pdf]

Appendix

Tenascin-C orchestrates radiotherapy-induced head and neck tumor regression

Thomas Loustau[1,2+]*, Ioanna Mitrentsi[1+], Nuohan Wang[1], Caroline Spenlé[1,3], Alexia Pavlidaki[1], Thibaud Tranchant[1], Gilles Riegel[1], Akhil Venu[1], Rime Oueidat[1], Manuel Koch[4], Marion Dumas[1], Fanny Wack[1], Aurelie Hirschler[5], Christine Carapito[5], Nicodème Paul[6], Raphael Carapito[6], Matthias Mörgelin[7], Uwe Hansen[8], Joyce Azzi[9,10], Lucie Aubergeon[11], Nathalie Salomé[1], Sayda Dhaouadi[12], Pierre Grenot[13], Balkiss Bouhaouala-Zahar[12], Simona La Cioppa[14], Philippe Oertle[14], Valerio Izzi[15], Marija Plodinec[14], Georges Noel[9,10], Hélène Burckel[9,10] and Gertraud Orend[1]*

Afiliations:

[1] INSERM U1109, The Tumor Microenvironment Laboratory, Hôpital Civil, Institut d'Hématologie et d'Immunologie, 67091 Strasbourg, France

[2] University of Strasbourg, UPR CNRS 9002, ARN, IUT Louis Pasteur, 1 allée d'Athènes, 67300 Schiltigheim, France

[3] University of Strasbourg, ESBS, INSERM-ERL1321, groupe Biothérapie peptidique, Pôle Api, Bâtiment D, 300 Boulevard Sébastien Brant, 67400 Illkirch, France

[4] University Cologne, Faculty of Medicine and University Hospital Cologne, Center for Dental, Oral and Maxillofacial Medicine (central facilities), Cologne, Germany

[5] Laboratoire de Spectrométrie de Masse BioOrganique, Institut Pluridisciplinaire Hubert Curien (IPHC), UMR 7178, Université de Strasbourg, CNRS, Infrastructure Nationale de Protéomique ProFi - UAR2048, 67000 Strasbourg, France.

[6] Laboratoire d'ImmunoRhumatologie Moléculaire, INSERM UMR_S 1109, Plateforme GENOMAX, ITI Médecine de Précision de Strasbourg, Transplantex NG, Faculté de Médecine, Fédération Hospitalo-Universitaire OMICARE, France

[7] Colzyx AB, Lund, Sweden

[8] Institute for Musculoskeletal Medicine (IMM), University Hospital Muenster, Muenster, Germany

[9] Institut de Cancérologie Strasbourg Europe (ICANS), UNICANCER, Radiobiology Laboratory, Paul Strauss Comprehensive, Cancer Center, 67000 Strasbourg, France.

[10] ICube, UMR7357, Equipe Imagerie Multimodale Intégrative en Santé, Université de Strasbourg, France

[11] University of Strasbourg, UPR CNRS 3572, I2CT, 2 allée Konrad Roentgen 67084 Strasbourg, France

[12] Pasteur Institute of Tunis, NanoBioMedika research Team, 13 Place Pasteur - B.P. 74 - 1002 Tunis, Tunisia.

[13] Laboratoire d'ImmunoRhumatologie Moléculaire, INSERM UMR_S 1109, Plateforme CYTOMAX, Institut d'Hématologie et d'Immunologie, 67091 Strasbourg, France.

[14] Artidis, Hochbergerstrasse 60c, 4057 Basel, Switzerland

[15] Faculty of Biochemistry and Molecular Medicine at University of Oulu, Oulu, Finland

+ Co-first authorship

* Corresponding authors:

Thomas Loustau, INSERM U1109, The Tumor Microenvironment laboratory,

thomas.loustau@unistra.fr

Gertraud Orend, INSERM U1109, The Tumor Microenvironment laboratory,

gertraud.orend@inserm.fr

Appendix	Description	Page
**Appendix Figure S1**	Characterization and flow cytometry immunoprofiling of the 4NQO tumors and associated tumor draining lymph nodes after irradiation in WT and TNCKO mice	3-6
**Appendix Figure S2**	TNC expression plays a pivotal role in determining the FRC identity	7-8
**Appendix Figure S3**	The FRC cell response to irradiation is determined by TNC expression	9-12
**Appendix Figure S4**	TNC impact on the OSCC/FRC crosstalk upon irradiation	13-15
**Appendix Figure S5**	Characterization of neck coengrafted tumors in C57Bl6 mice	16-17
**Appendix Figure S6**	Targeting TNC with MAREMO peptide MP5 reduces tumor cell numbers and plasticity upon IR	18-20
**Appendix Table S1**	Differential gene expression in WT vs TNCKO TdLNs	21-29
**Appendix Table S2**	Differential gene expression in WT FRCs in comparison to TNCKO FRCs	29-31
**Appendix Table S3**	Differential protein expression in FRCs in dependence of TNC and IR	32-42
**Appendix Table S4**	Cox regression analysis for HNSCC RT cohort	43-45
**Appendix Table S5**	P values	46-56

Figure S1

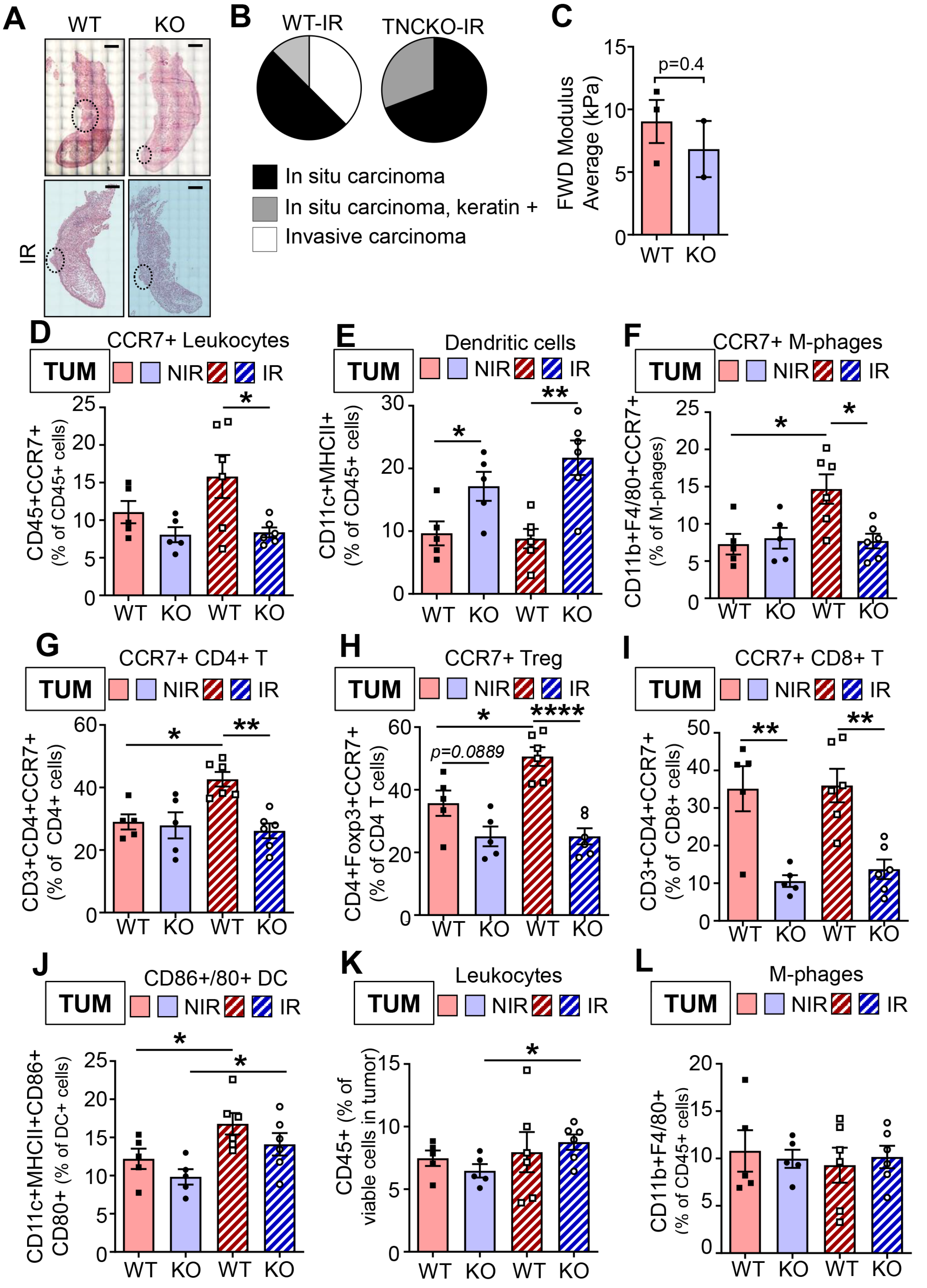

A
WT
KO
IR
B
WT-IR
TNCKO-IR
In situ carcinoma
In situ carcinoma, keratin +
Invasive carcinoma
C
FWD Modulus Average (kPa)
p=0.4
WT KO
D
CCR7+ Leukocytes
TUM NIR IR
CD45+CCR7+ (% of CD45+ cells)
E
Dendritic cells
CD11c+MHCII+ (% of CD45+ cells)
F
CCR7+ M-phages
CD11b+F4/80+CCR7+ (% of M-phages)
G
CCR7+ CD4+ T
CD3+CD4+CCR7+ (% of CD4+ cells)
H
CCR7+ Treg
p=0.0889
CD4+Foxp3+CCR7+ (% of CD4 T cells)
I
CCR7+ CD8+ T
CD3+CD4+CCR7+ (% of CD8+ cells)
J
CD86+/80+ DC
CD11c+MHCII+CD86+ CD80+ (% of DC+ cells)
K
Leukocytes
CD45+ (% of viable cells in tumor)
L
M-phages
CD11b+F4/80+ (% of CD45+ cells)
WT KO WT KO

Figure S1

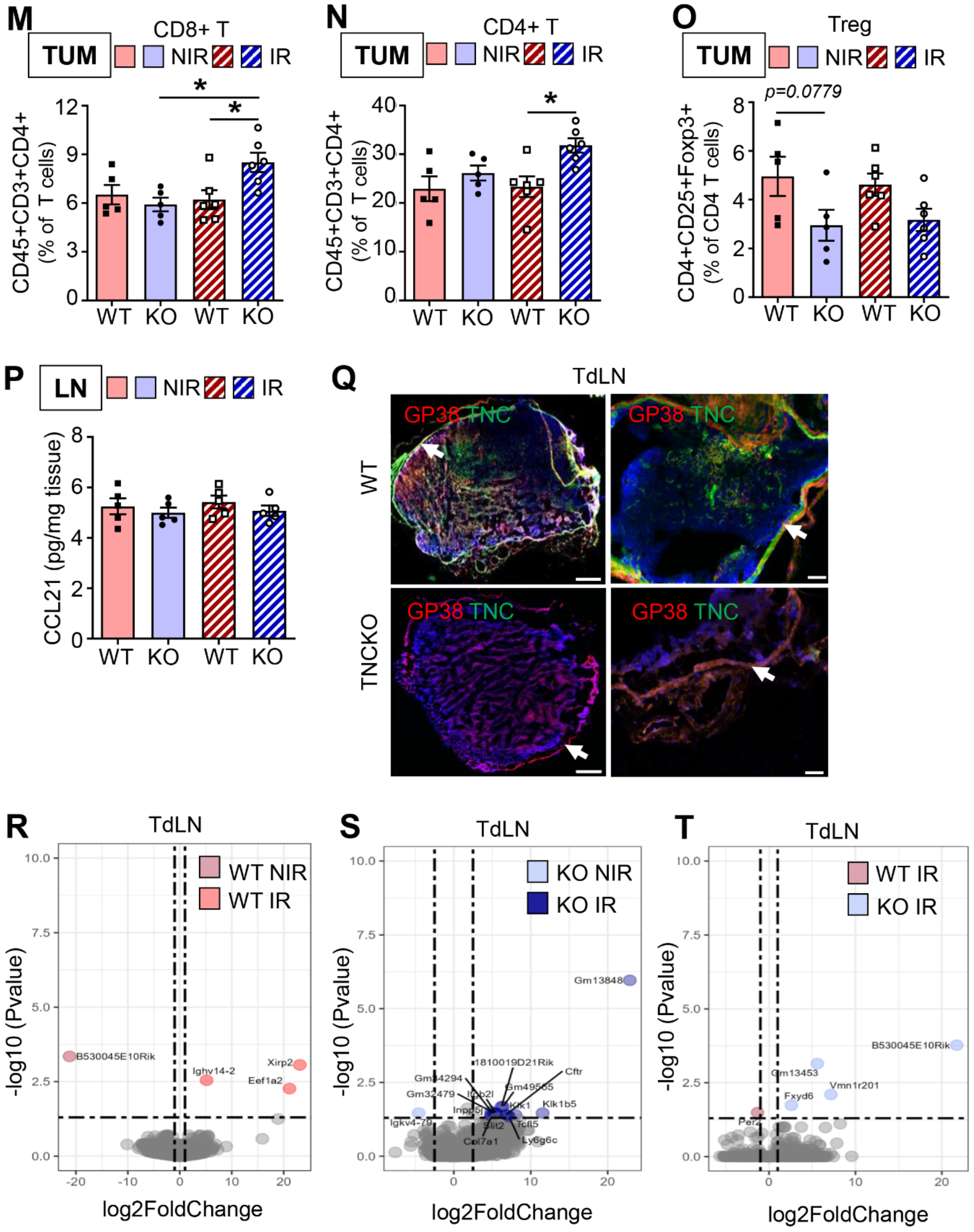

M
CD8+ T
TUM
NIR
IR
CD45+CD3+CD4+ (% of T cells)
WT KO WT KO
N
CD4+ T
TUM
NIR
IR
CD45+CD3+CD4+ (% of T cells)
WT KO WT KO
O
Treg
TUM
NIR
IR
p=0.0779
CD4+CD25+Foxp3+ (% of CD4 T cells)
WT KO WT KO
P
LN
NIR
IR
CCL21 (pg/mg tissue)
WT KO WT KO
Q
TdLN
GP38 TNC
WT
TNCKO
R
TdLN
WT NIR
WT IR
B530045E10Rik
Ighv14-2
Xirp2
Eef1a2
-log10 (Pvalue)
log2FoldChange
S
TdLN
KO NIR
KO IR
Gm13848
1810019D21Rik
Cftr
Gm49565
Klk1
Klk1b5
Tcfl5
Slit2
Ly6g6c
Col7a1
Igkv4-79
-log10 (Pvalue)
log2FoldChange
T
TdLN
WT IR
KO IR
B530045E10Rik
Gm13453
Vmn1r201
Fxyd6
Per2
-log10 (Pvalue)
log2FoldChange

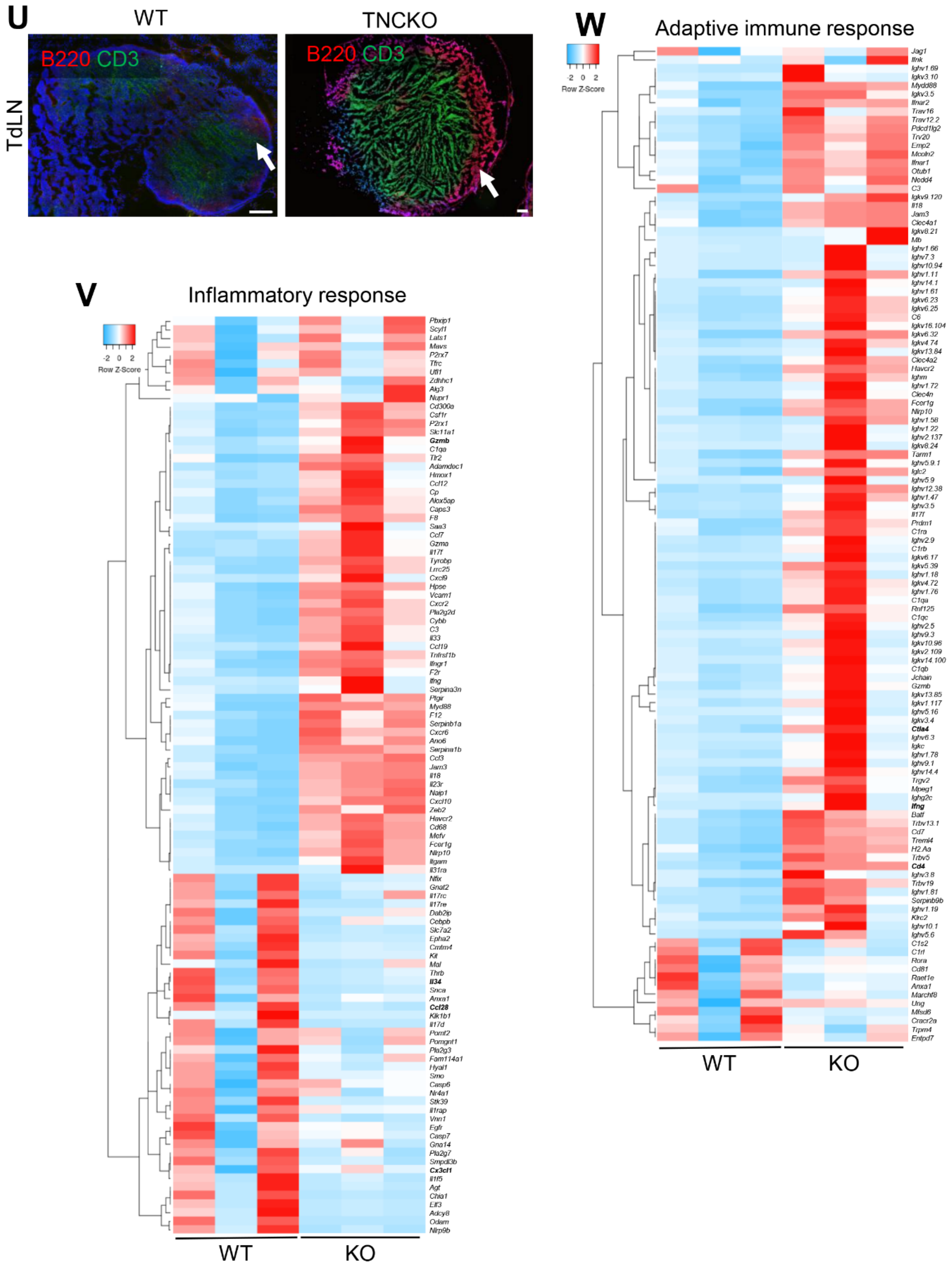
U
WT
TNCKO
TdLN
B220 CD3
B220 CD3
V
Inflammatory response
Row Z-Score
-2 0 2
WT
KO
W
Adaptive immune response
Row Z-Score
-2 0 2
WT
KO

Appendix Figure S1: Characterization and flow cytometry immunoprofiling of the 4NQO tumors and associated TdLNs after irradiation in WT and TNCKO mice (**A**) Representative composite images of hematoxylin and eosin-stained cross sections (n = 20) from tongues of 4NQO-treated WT and TNCKO mice non-irradiated (NIR) or irradiated (IR). The black circles indicate the tongue tumor. (**B**) Stage classification of radioresistant tongue tumors in WT and TNCKO mice upon IR. Lesions (n = 8 KO, 10 WT) were classified in differentiated squamous cell carcinoma (black), in situ carcinoma expressing keratin (gray) or invasive carcinoma (white). (**C**) Forward (FWD) E-Modulus average (kPA) of the corresponding Atomic Force Microscopy (AFM) measurements with passed quality check. Between 9 and 27 measurements were done per tumor sample. N = 2 or 3 tumors per condition. Mean ± SEM; Mann-Whitney test. (**D-O**) FACS analysis of NIR and IR 4NQO tumors (TUM) of WT and TNCKO mice for CCR7+ leukocytes (**D**), dendritic cells (**E**), CCR7+ macrophages (**F**), CCR7+ CD4+ T cells (**G**), CCR7+ Treg (**H**), CCR7+ CD8+ T cells (**I**), CD86/80+ DC (**J**), CD45+ leukocytes (**K**), macrophages (**L**), CD8+ T cells (**M**), CD4+ T cells (**N**) and T regulatory (Treg) cells (**O**). (**P**) ELISA for CCL21 in NIR and IR 4NQO TdLNs of 4NQO exposed WT and TNCKO mice. (**Q**) Representative IF images for GP38 and TNC in the TdLNs of WT and TNC KO mice in a lower (left panel) or higher (right panel) maginification. Scale bars, 50 µm. Arrows point at the reticular fibers. The IF image of the WT TdLN (upper left panel) is used in the summary cartoon of **Fig 7C.** N = 5 TdLNs per group. **(R-T)** Volcano plots of deregulated genes after RNA sequencing of the TdLNs of WT mice NIR or after 2Gy IR (**R**), TNC KO mice NIR or after 2Gy IR (**S**) and WT or TNC KO mice after 2Gy IR (**T**). n= 3 TdLNs per group. (**U**) Representative IF images for B220 and CD3 in the TdLNs of WT and TNCKO. n= 5 TdLNs per group. Scale bars, 50 µm. (**V, W**) Heatmap representation of gene expression data (derived from RNA seq analysis) of TdLNs from WT compared to TNCKO tumor mice. P <0.05. Mean ± SEM; Kruskal–Wallis test and Dunn post-test, *, P < 0.05, **, P < 0.01, ***, P<0.005. The exact p values are listed in **Appendix Table S5**.

Figure S2

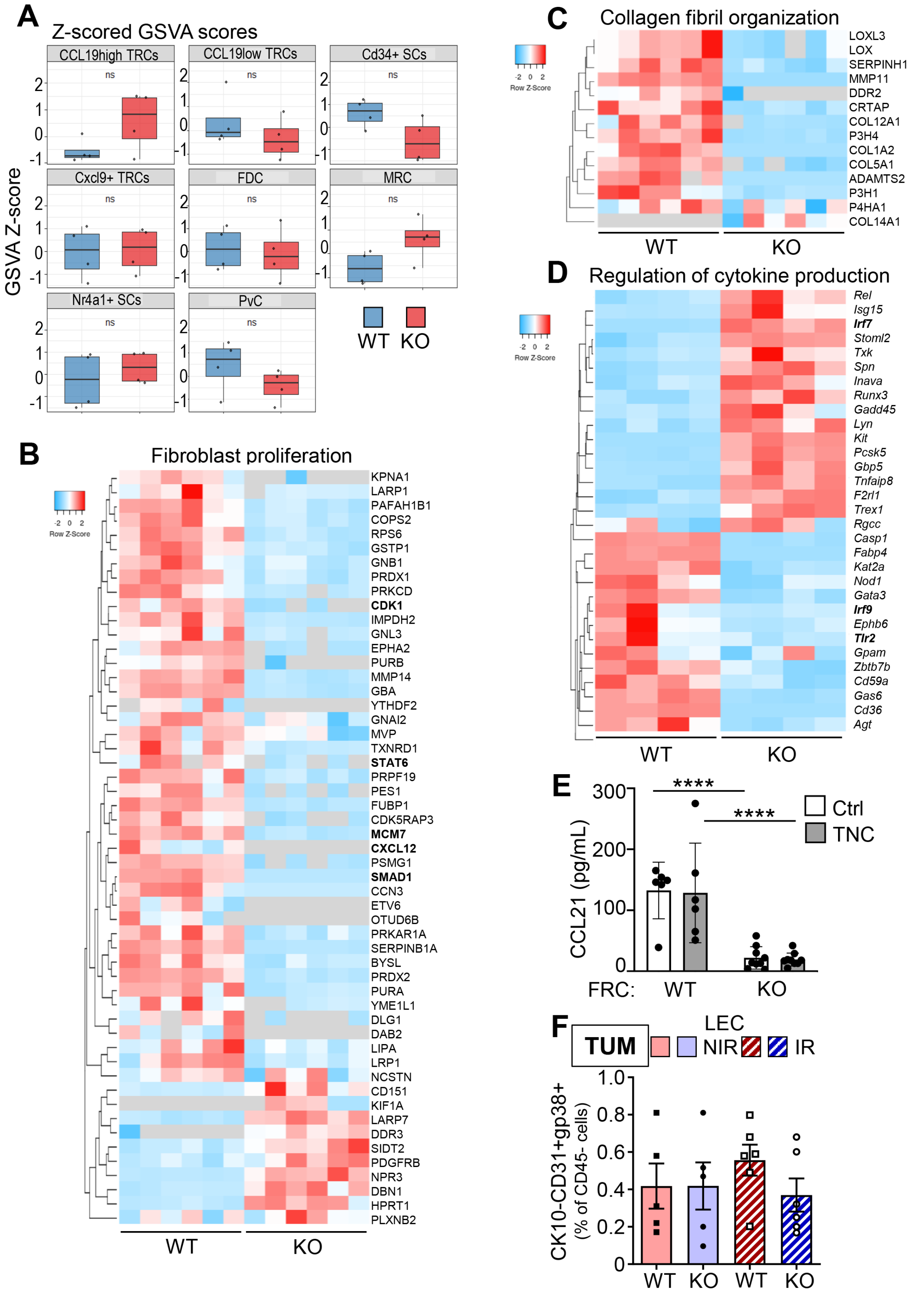
A
Z-scored GSVA scores
GSVA Z-score
CCL19high TRCs
CCL19low TRCs
Cd34+ SCs
Cxcl9+ TRCs
FDC
MRC
Nr4a1+ SCs
PvC
ns
WT KO
B
Fibroblast proliferation
Row Z-Score
KPNA1
LARP1
PAFAH1B1
COPS2
RPS6
GSTP1
GNB1
PRDX1
PRKCD
CDK1
IMPDH2
GNL3
EPHA2
PURB
MMP14
GBA
YTHDF2
GNAI2
MVP
TXNRD1
STAT6
PRPF19
PES1
FUBP1
CDK5RAP3
MCM7
CXCL12
PSMG1
SMAD1
CCN3
ETV6
OTUD6B
PRKAR1A
SERPINB1A
BYSL
PRDX2
PURA
YME1L1
DLG1
DAB2
LIPA
LRP1
NCSTN
CD151
KIF1A
LARP7
DDR3
SIDT2
PDGFRB
NPR3
DBN1
HPRT1
PLXNB2
WT
KO
C
Collagen fibril organization
LOXL3
LOX
SERPINH1
MMP11
DDR2
CRTAP
COL12A1
P3H4
COL1A2
COL5A1
ADAMTS2
P3H1
P4HA1
COL14A1
WT
KO
D
Regulation of cytokine production
Rel
Isg15
Irf7
Stoml2
Txk
Spn
Inava
Runx3
Gadd45
Lyn
Kit
Pcsk5
Gbp5
Tnfaip8
F2rl1
Trex1
Rgcc
Casp1
Fabp4
Kat2a
Nod1
Gata3
Irf9
Ephb6
Tlr2
Gpam
Zbtb7b
Cd59a
Gas6
Cd36
Agt
WT
KO
E
CCL21 (pg/mL)
****
****
Ctrl
TNC
FRC: WT KO
F
TUM
LEC
NIR
IR
CK10-CD31+gp38+
(% of CD45- cells)
WT KO WT KO

Appendix Figure S2: TNC expression plays a pivotal role in determining the FRC identity (**A**) RNA sequencing Gene Set Variation Analysis (GSVA) for the different FRC subclusters in the WT and TNCKO FRCs. Proteomics data analysis (**B, C**) represented as heatmaps for the most deregulated proteins that belong to fibroblast proliferation (**B**) and collagen fibril organization (**C**). $P < 0.05$. (**D**) RNA seq gene expression analysis represented as heatmap for deregulated gene members of the regulation of cytokine production. Bold text indicates genes/proteins with established roles in the respective categories and/or that are discussed in the text. $P < 0.05$. (**E**) Quantification of CCL21 by ELISA in WT or TNCKO FRCs, either non-treated control (Ctrl) or treated with soluble TNC (10 μg/mL) for 24 hours. Mean ± SEM; Kruskal–Wallis test and Dunn posttest, *, $P < 0.05$. (**F**) FACS analysis of lymphatic endothelial cells in NIR and IR 4NQO tumors (Tum) of WT and TNCKO mice. Mean ± SEM; Kruskal–Wallis test and Dunn post-test. The exact p values are listed in **Appendix Table S5**.

Figure S3

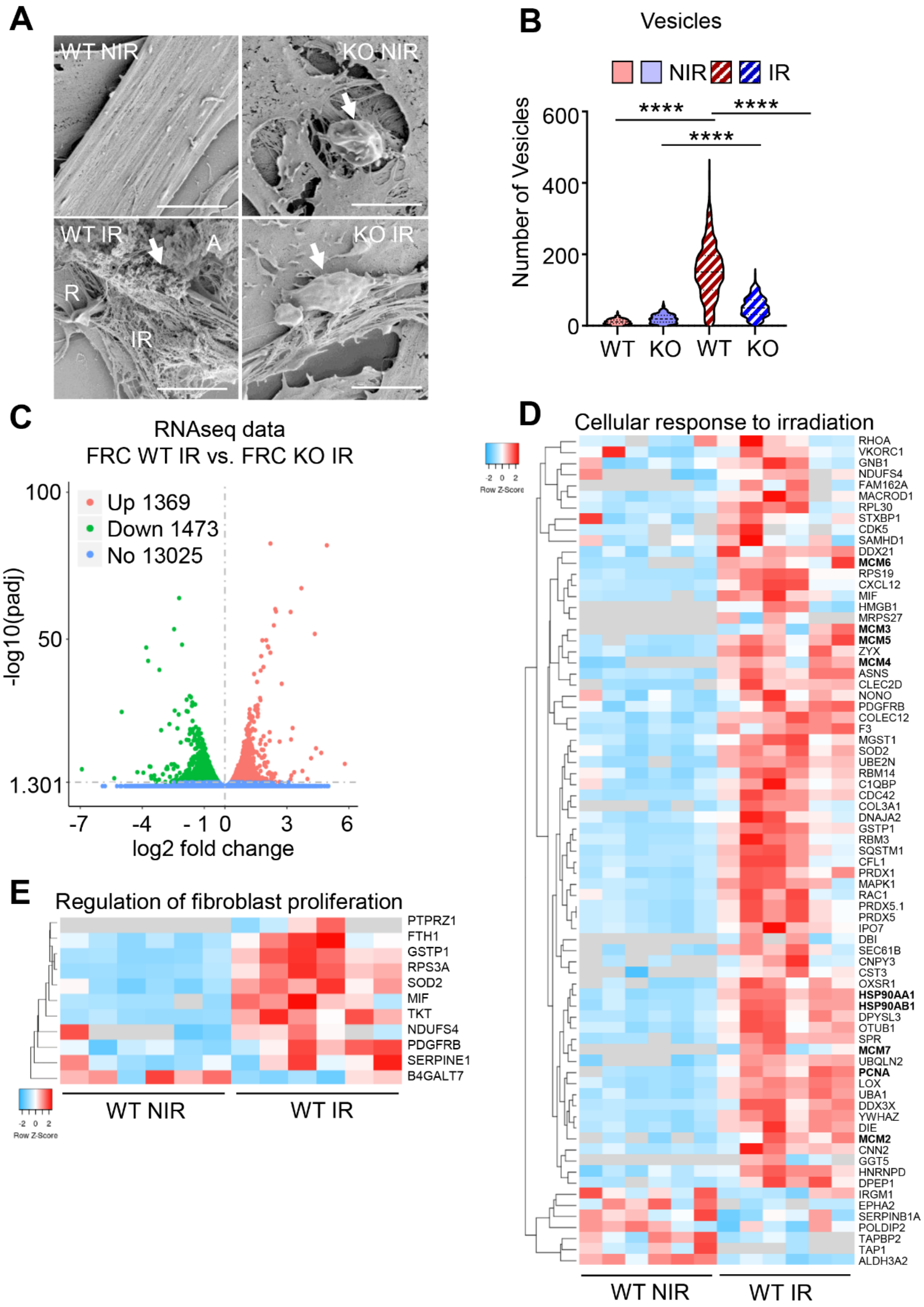
A
WT NIR
KO NIR
WT IR
A
R
IR
KO IR
B
Vesicles
NIR
IR
Number of Vesicles
600
400
200
0
****
****
****
WT
KO
WT
KO
C
RNAseq data
FRC WT IR vs. FRC KO IR
Up 1369
Down 1473
No 13025
-log10(padj)
100
50
1.301
-7
-4
- 1 0
3
6
log2 fold change
D
Cellular response to irradiation
-2 0 2
Row Z-Score
RHOA
VKORC1
GNB1
NDUFS4
FAM162A
MACROD1
RPL30
STXBP1
CDK5
SAMHD1
DDX21
MCM6
RPS19
CXCL12
MIF
HMGB1
MRPS27
MCM3
MCM5
ZYX
MCM4
ASNS
CLEC2D
NONO
PDGFRB
COLEC12
F3
MGST1
SOD2
UBE2N
RBM14
C1QBP
CDC42
COL3A1
DNAJA2
GSTP1
RBM3
SQSTM1
CFL1
PRDX1
MAPK1
RAC1
PRDX5.1
PRDX5
IPO7
DBI
SEC61B
CNPY3
CST3
OXSR1
HSP90AA1
HSP90AB1
DPYSL3
OTUB1
SPR
MCM7
UBQLN2
PCNA
LOX
UBA1
DDX3X
YWHAZ
DIE
MCM2
CNN2
GGT5
HNRNPD
DPEP1
IRGM1
EPHA2
SERPINB1A
POLDIP2
TAPBP2
TAP1
ALDH3A2
WT NIR
WT IR
E
Regulation of fibroblast proliferation
PTPRZ1
FTH1
GSTP1
RPS3A
SOD2
MIF
TKT
NDUFS4
PDGFRB
SERPINE1
B4GALT7
-2 0 2
Row Z-Score
WT NIR
WT IR

Figure S3

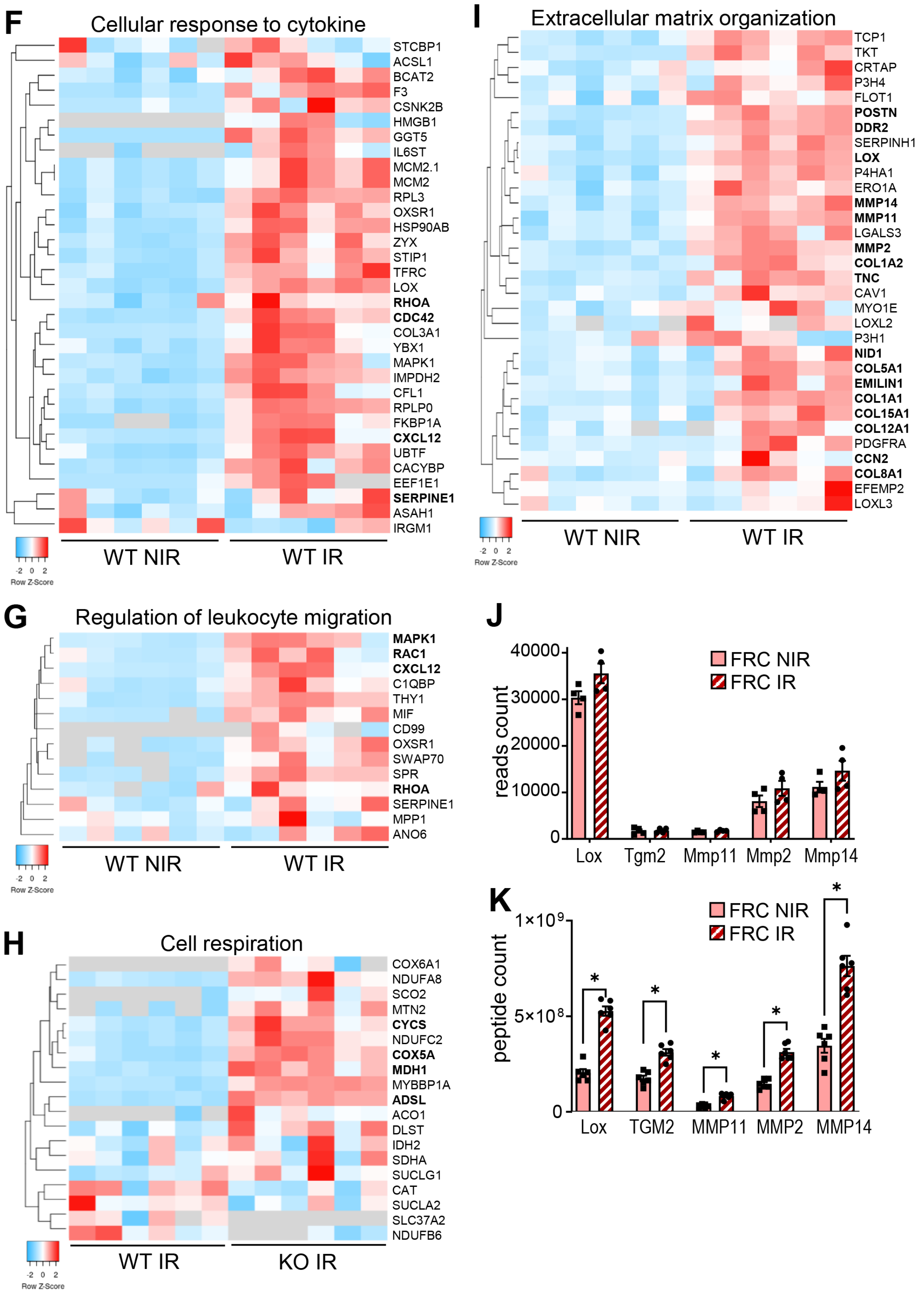
F
Cellular response to cytokine
STCBP1
ACSL1
BCAT2
F3
CSNK2B
HMGB1
GGT5
IL6ST
MCM2.1
MCM2
RPL3
OXSR1
HSP90AB
ZYX
STIP1
TFRC
LOX
RHOA
CDC42
COL3A1
YBX1
MAPK1
IMPDH2
CFL1
RPLP0
FKBP1A
CXCL12
UBTF
CACYBP
EEF1E1
SERPINE1
ASAH1
IRGM1
WT NIR
WT IR
-2 0 2
Row Z-Score
I
Extracellular matrix organization
TCP1
TKT
CRTAP
P3H4
FLOT1
POSTN
DDR2
SERPINH1
LOX
P4HA1
ERO1A
MMP14
MMP11
LGALS3
MMP2
COL1A2
TNC
CAV1
MYO1E
LOXL2
P3H1
NID1
COL5A1
EMILIN1
COL1A1
COL15A1
COL12A1
PDGFRA
CCN2
COL8A1
EFEMP2
LOXL3
WT NIR
WT IR
-2 0 2
Row Z-Score
G
Regulation of leukocyte migration
MAPK1
RAC1
CXCL12
C1QBP
THY1
MIF
CD99
OXSR1
SWAP70
SPR
RHOA
SERPINE1
MPP1
ANO6
WT NIR
WT IR
-2 0 2
Row Z-Score
J
reads count
40000
30000
20000
10000
0
FRC NIR
FRC IR
Lox
Tgm2
Mmp11
Mmp2
Mmp14
K
peptide count
1×10⁹
5×10⁸
0
FRC NIR
FRC IR
*
Lox
TGM2
MMP11
MMP2
MMP14
H
Cell respiration
COX6A1
NDUFA8
SCO2
MTN2
CYCS
NDUFC2
COX5A
MDH1
MYBBP1A
ADSL
ACO1
DLST
IDH2
SDHA
SUCLG1
CAT
SUCLA2
SLC37A2
NDUFB6
WT IR
KO IR
-2 0 2
Row Z-Score

Figure S3

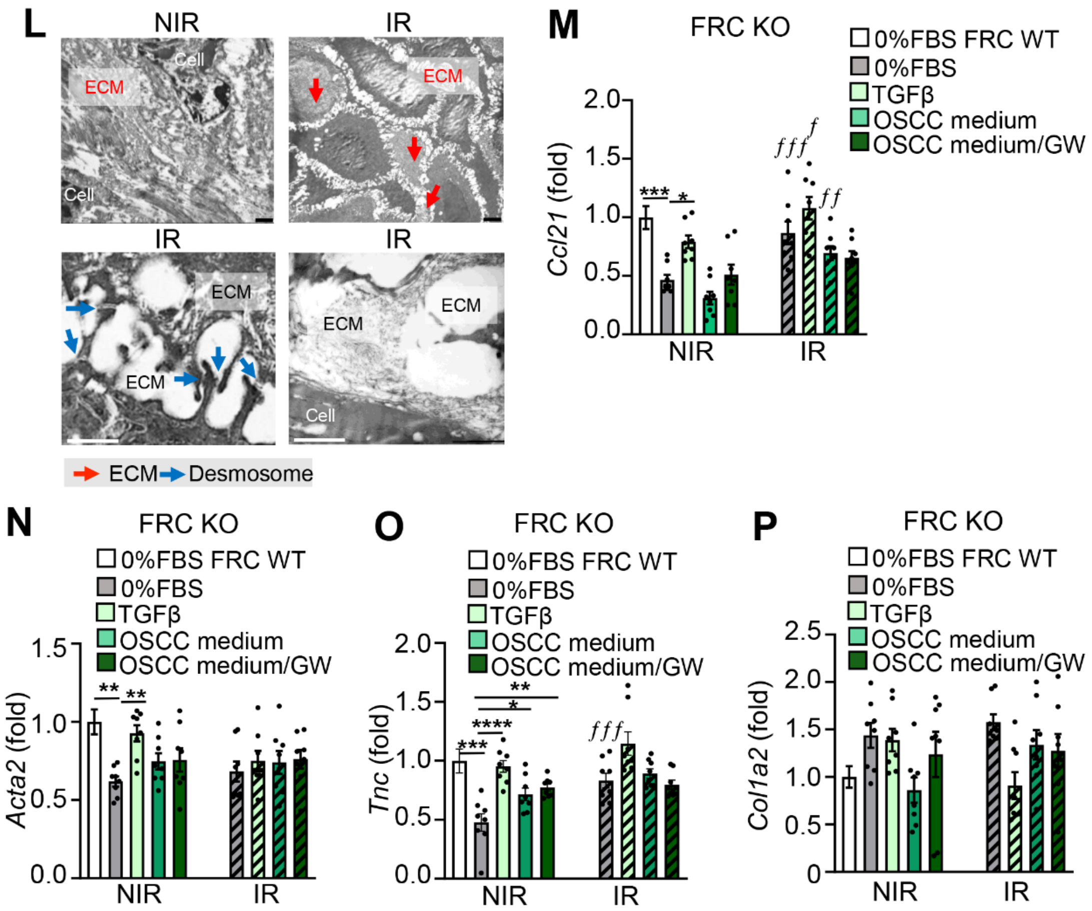

Appendix Figure S3: The FRC cell response to irradiation is determined by TNC expression (**A**) Representative SEM images of non-irradiated (NIR) or irradiated (IR) FRC WT and FRC TNCKO. Scale bar, 5µm. Images show regular (R), irregular (IR) and amorphous (A) fibril organization. (**B**) Cell vesicles quantification determined after the analysis of 300 SEM images of each cell condition. Mean ±SD; One way – ANOVA and Tukey's multiple comparisons post-test. (**C**) Volcano plot representation of deregulated genes (DEGs) obtained after RNA sequencing of irradiated FRC WT and TNCKO showing the fold change and the adjusted p-value for the 15 867 genes expressed. Red dots (1 369) represent genes significantly overexpressed in irradiated WT compared to TNCKO FRCs. Proteomic analysis of WT FRC in NIR vs. IR comparison, represented as heatmaps for deregulated proteins involved in the cellular response to irradiation (**D**), regulation of fibroblast proliferation (**E**), cellular response to cytokines (**F**), regulation of leukocyte migration (**G**) and ECM organization (**I**). $P < 0.05$. (**H**) Heatmaps representing deregulated proteins involved in cell respiration in irradiated WT vs. TNCKO FRCs. $P < 0.05$. (**J, K**) Gene reads counts (**J**) and peptide counts (**K**) of LOX, TGM2, MMP11, MMP2 and MMP14 in WT FRCs NIR vs IR. (**L**) EM images of NIR and IR tumors as indicated. Scale bar, 500 nm (upper panels), 1000 nm (lower panels). ECM (red) and desmosomes (blue) are indicated by

arrows. (**M-P)** Gene expression as determined by qRTPCR in FRC TNCKO for *Ccl21*, *Acta2*, *Tnc* and *Col1a2* after incubation with the indicated conditions. One-way ordinary ANOVA with * P < 0.05, ** P < 0.01, *** P < 0.005. The exact p values are listed in **Appendix Table S5**.

Figure S4

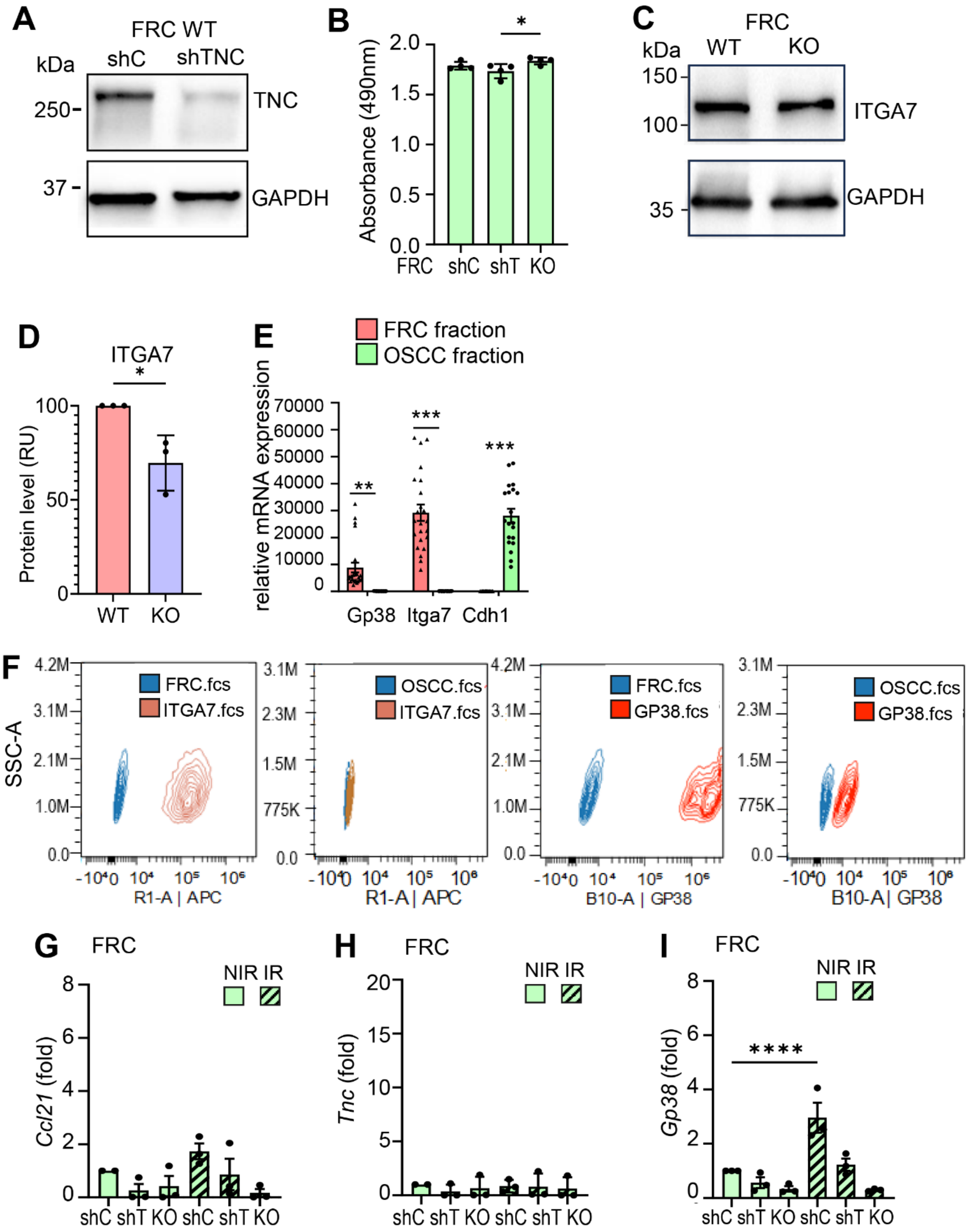

A
FRC WT
kDa
shC
shTNC
250
TNC
37
GAPDH
B
Absorbance (490nm)
2.0
1.5
1.0
0.5
0.0
FRC
shC
shT
KO
*
C
FRC
kDa
WT
KO
150
100
ITGA7
35
GAPDH
D
ITGA7
*
Protein level (RU)
100
50
0
WT
KO
E
FRC fraction
OSCC fraction
relative mRNA expression
70000
60000
50000
40000
30000
20000
10000
0
**
***
***
Gp38
Itga7
Cdh1
F
SSC-A
FRC.fcs
ITGA7.fcs
OSCC.fcs
ITGA7.fcs
FRC.fcs
GP38.fcs
OSCC.fcs
GP38.fcs
R1-A | APC
R1-A | APC
B10-A | GP38
B10-A | GP38
G
FRC
NIR IR
Ccl21 (fold)
shC shT KO shC shT KO
H
FRC
NIR IR
Tnc (fold)
shC shT KO shC shT KO
I
FRC
NIR IR
Gp38 (fold)
****
shC shT KO shC shT KO

Figure S4

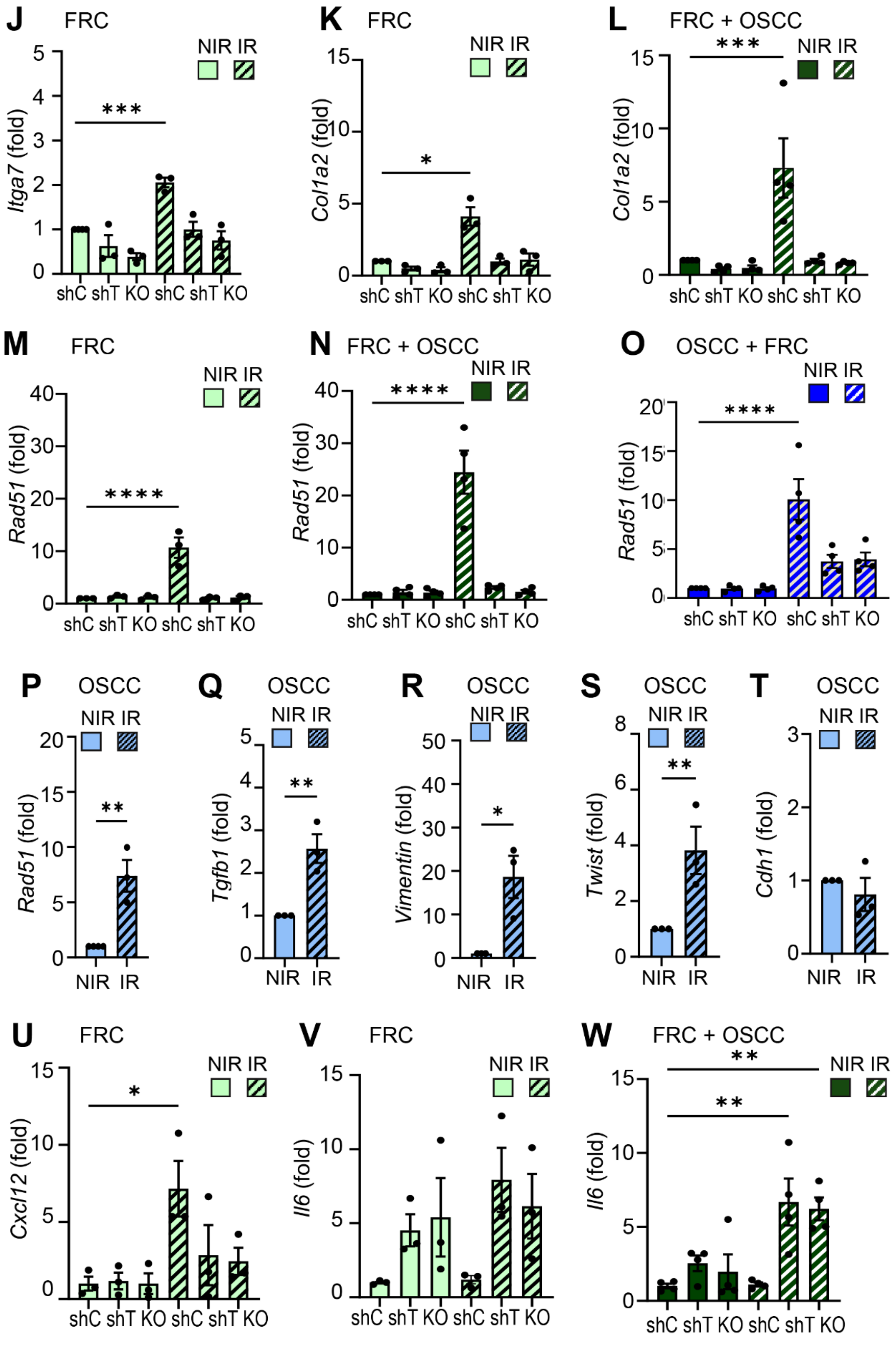
J FRC
K FRC
L FRC + OSCC
M FRC
N FRC + OSCC
O OSCC + FRC
P OSCC
Q OSCC
R OSCC
S OSCC
T OSCC
U FRC
V FRC
W FRC + OSCC
NIR IR
Itga7 (fold)
Col1a2 (fold)
Rad51 (fold)
Tgfb1 (fold)
Vimentin (fold)
Twist (fold)
Cdh1 (fold)
Cxcl12 (fold)
Il6 (fold)
shC shT KO shC shT KO
NIR IR

Appendix Figure S4: **TNC impact on the OSCC/FRC crosstalk upon irradiation (A)** TNC expression (western blot) in shC and shTNC FRCs. GAPDH is used as control. **(B)** Absorbance at 490 nm after an MTS assay in shC, shTNC and TNCKO FRCs. **(C, D)** Expression of ITGA7 (western blot) in WT and TNCKO FRCs. GAPDH is used as control **(E)** Gene expression (qRTPCR) analysis of *Gp38, Cdh1* and *Itga7* upon OSCC13/FRC coculture, followed by isolation of cells by MACS. Note, that OSCC13 cells express *Cdh1* but not *Gp38* nor *Itga7*, while FRCs express *Gp38* and *Itga7* but not *Cdh1*. **(F)** FACS analysis of GP38 and ITGA7 in OSCC13 and FRC cells. **(G-N, U-W)** Gene expression (qRTPCR) analysis of *Ccl21* **(G)**, *Tnc* **(H)**, *Gp38* **(I)** , *Itga7* **(J)**, *Col1a2* **(K-L), ***Rad51* **(M-N),** *Cxcl12* **(U)** and *Il6* **(V-W)** in FRC monocultures (light green) or in the sorted cocultures with OSCC13 (dark green), before (NIR) or 2 days after 2 Gy irradiation (IR). **(O-T)** Gene expression (qRTPCR) analysis of *Rad51* (**O-P**)*, Tgfb1* **(Q)**, *vimentin* **(R)**, *Twist* **(S)** and *Cdh1* **(T)**, in OSCC13 monocultures (light blue) or in co-cultures with FRCs (shC, shTNC, KO) (dark blue), NIR or 2 days after 2 Gy irradiation (IR). Error bars represent mean ± SEM. N = 3. One way ordinary ANOVA test with * P < 0,05, ** P < 0,01, *** P < 0,001, **** P < 0,0001. The exact p values are listed in **Appendix Table S5**.

Figure S5

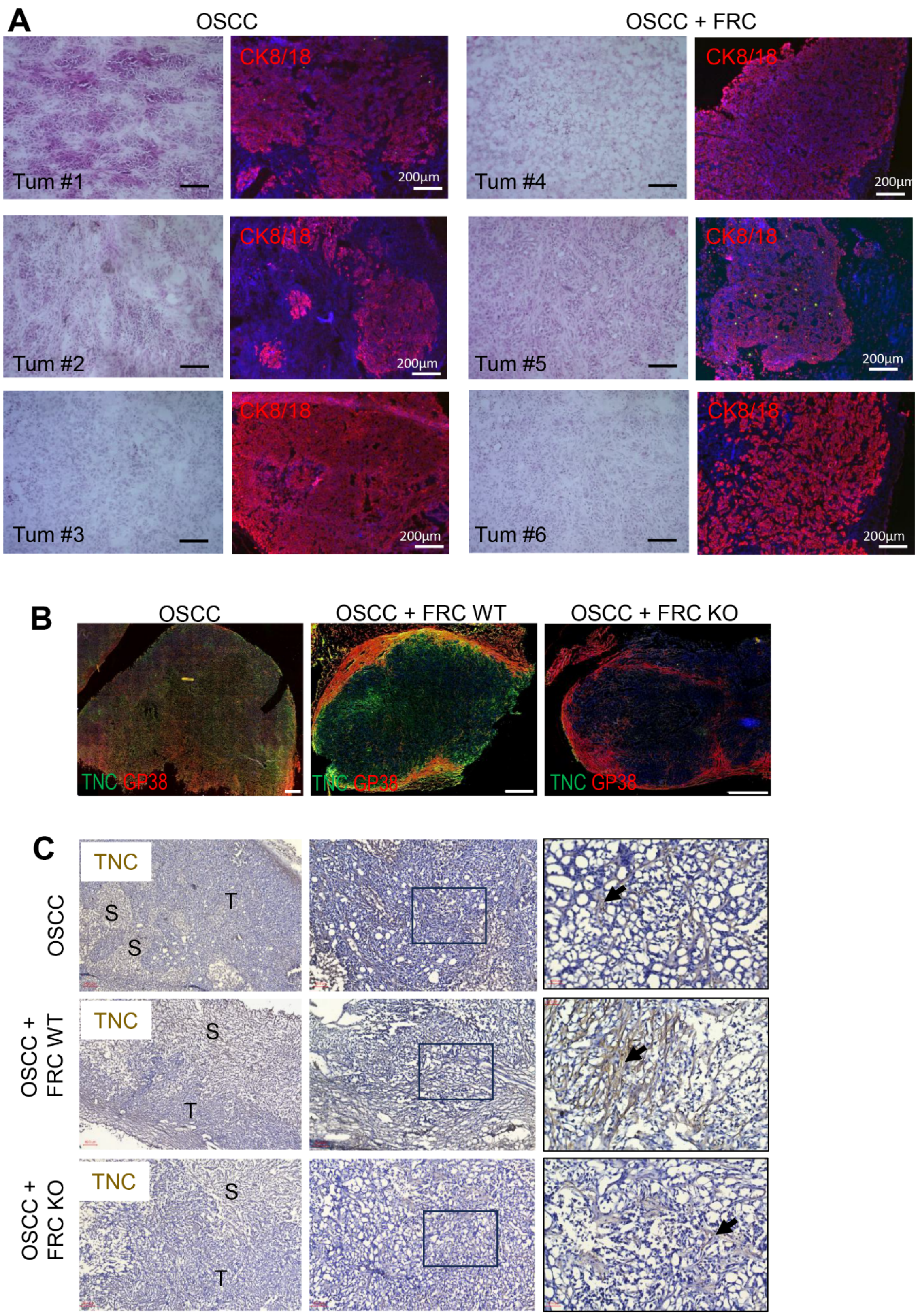

A
OSCC
OSCC + FRC
CK8/18
Tum #1
200µm
Tum #4
Tum #2
Tum #5
Tum #3
Tum #6
B
OSCC
OSCC + FRC WT
OSCC + FRC KO
TNC GP38
C
TNC
S
T
OSCC
OSCC + FRC WT
OSCC + FRC KO

Appendix Figure S5: Characterization of neck engrafted tumors in C57Bl6 mice (A) Staining of hematoxylin-eosin (HE) and IF staining in sections from 6 representative neck tumors obtained after OSCC grafting (1.5 x 10^6 cells) or OSCC/FRC (5:1) co-grafting of cells in WT mice. The CK8/18 IF image corresponding to **Fig 5B** is included here alongside additional tumors and H&E staining. Scale bar, 200 µm (**B, C**) Representative immunofluorescence images of TNC and GP38 (**B**) or Immunohistochemistry (IHC) images of TNC (**C**) in the tumors upon engraftment of OSCC cells alone (3 x 10^6 cells) or combined with WT FRC, or combined with FRC TNCKO cells, in a 5:1 ratio into the neck of WT mice, showing the tumor nests (S) and stroma (T) in the different conditions. OSCC indicates OSCC13 cells. Arrow points at TNC. Note that TNC is more abundant in FRC WT engrafted tumors. The IF image of the OSCC/FRC WT tumor shown in (**B**) is used in the summary cartoon of **Fig 7C.** Scale bar, 50 µm

Figure S6

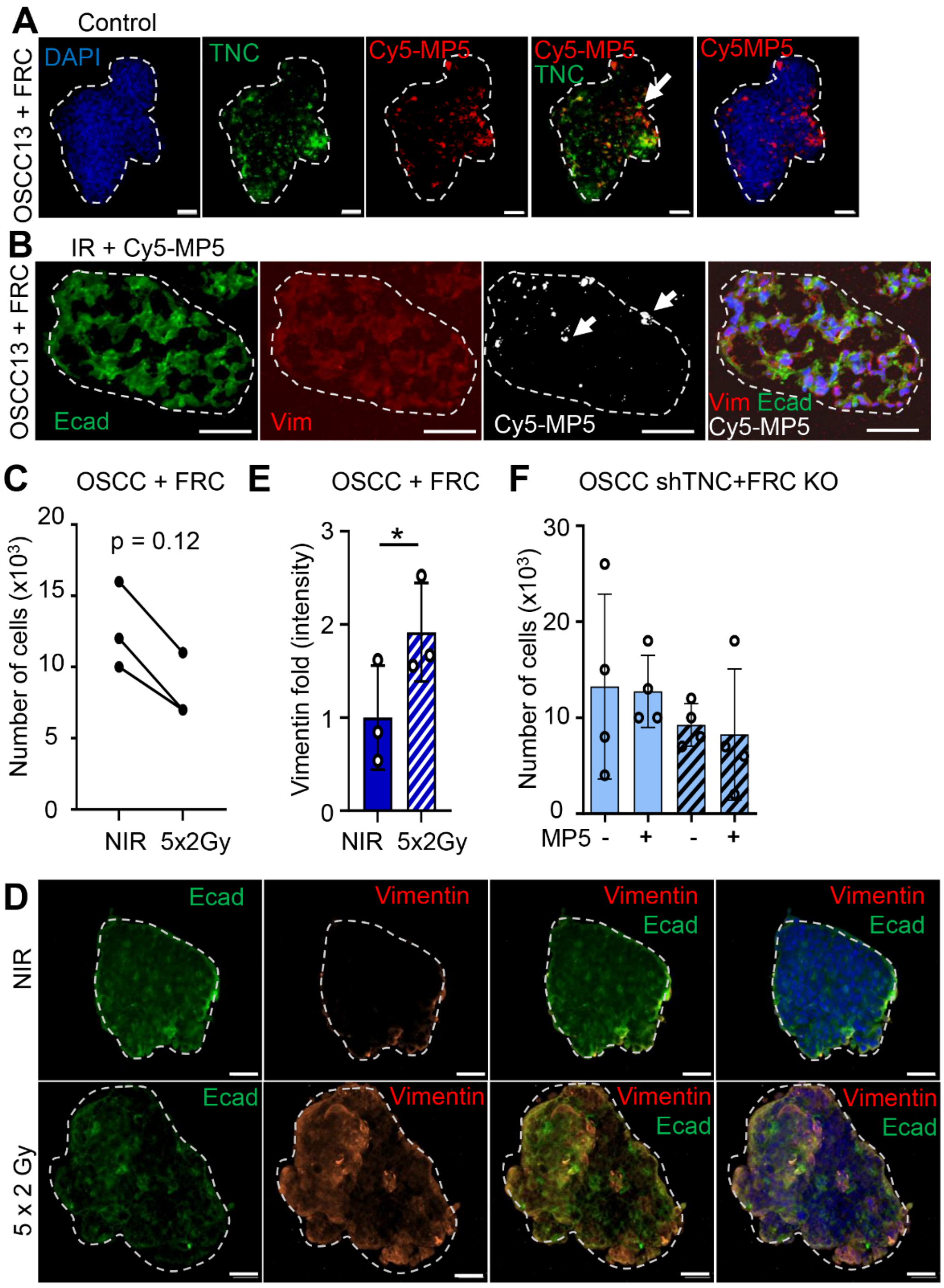
A
Control
OSCC13 + FRC
DAPI
TNC
Cy5-MP5
Cy5-MP5
TNC
Cy5MP5
B
IR + Cy5-MP5
OSCC13 + FRC
Ecad
Vim
Cy5-MP5
Vim Ecad
Cy5-MP5
C
OSCC + FRC
p = 0.12
Number of cells (x10³)
20
15
10
5
0
NIR
5x2Gy
E
OSCC + FRC
Vimentin fold (intensity)
*
3
2
1
0
NIR
5x2Gy
F
OSCC shTNC+FRC KO
Number of cells (x10³)
30
20
10
0
MP5
-
+
-
+
D
NIR
5 x 2 Gy
Ecad
Vimentin
Vimentin
Ecad
Vimentin
Ecad

Figure S6

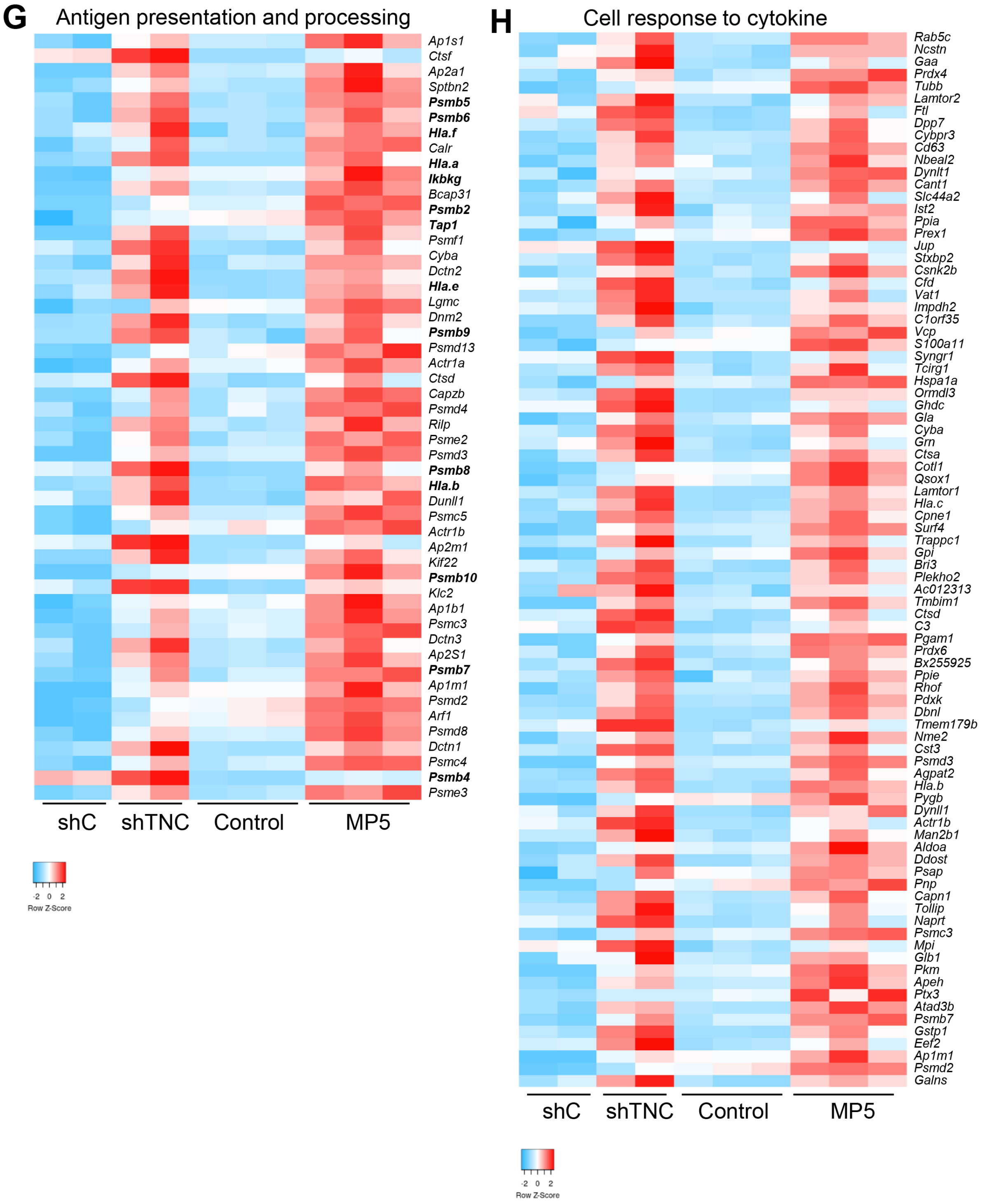
G
Antigen presentation and processing
Ap1s1
Ctsf
Ap2a1
Sptbn2
Psmb5
Psmb6
Hla.f
Calr
Hla.a
Ikbkg
Bcap31
Psmb2
Tap1
Psmf1
Cyba
Dctn2
Hla.e
Lgmc
Dnm2
Psmb9
Psmd13
Actr1a
Ctsd
Capzb
Psmd4
Rilp
Psme2
Psmd3
Psmb8
Hla.b
Dunll1
Psmc5
Actr1b
Ap2m1
Kif22
Psmb10
Klc2
Ap1b1
Psmc3
Dctn3
Ap2S1
Psmb7
Ap1m1
Psmd2
Arf1
Psmd8
Dctn1
Psmc4
Psmb4
Psme3
shC
shTNC
Control
MP5
-2 0 2
Row Z-Score
H
Cell response to cytokine
Rab5c
Ncstn
Gaa
Prdx4
Tubb
Lamtor2
Ftl
Dpp7
Cybpr3
Cd63
Nbeal2
Dynlt1
Cant1
Slc44a2
Ist2
Ppia
Prex1
Jup
Stxbp2
Csnk2b
Cfd
Vat1
Impdh2
C1orf35
Vcp
S100a11
Syngr1
Tcirg1
Hspa1a
Ormdl3
Ghdc
Gla
Cyba
Grn
Ctsa
Cotl1
Qsox1
Lamtor1
Hla.c
Cpne1
Surf4
Trappc1
Gpi
Bri3
Plekho2
Ac012313
Tmbim1
Ctsd
C3
Pgam1
Prdx6
Bx255925
Ppie
Rhof
Pdxk
Dbnl
Tmem179b
Nme2
Cst3
Psmd3
Agpat2
Hla.b
Pygb
Dynll1
Actr1b
Man2b1
Aldoa
Ddost
Psap
Pnp
Capn1
Tollip
Naprt
Psmc3
Mpi
Glb1
Pkm
Apeh
Ptx3
Atad3b
Psmb7
Gstp1
Eef2
Ap1m1
Psmd2
Galns
shC
shTNC
Control
MP5
-2 0 2
Row Z-Score

Appendix Figure S6: Targeting TNC with MAREMO peptide MP5 reduces tumor cell numbers and plasticity upon IR (**A,B**) representative IF images of TNC (**A**) and Vim and Ecad (**B**) after addition of 50 ng Cy5-MP5 for 72h to OSCC13/FRC (2.1 ratio) spheroid cocultures. The individual fluorescence channels correspond to the composite images shown in **Fig 6A, E**. Spheroid cultures were exposed to 10 Gy IR followed by exposure to Cy5-MP5 for another 72h. (**C-E**) Cell number (**C**), representative IF images of Ecadherin and Vimentin (**D**) and quantification of vimentin intensity (**E**) before or after 5 doses of 2 Gy IR in OSCC13/FRC spheroid cocultures. (**F**) Cell number quantification of OSCC13 shTNC /FRC TNCKO spheroids before (NIR) or after 10 Gy IR and, with or without the addition of 50 ng MP5 for 72h. (**G, H**) Heatmap representation of gene expression data derived from RNA seq analysis of NT193 tumors of the indicated conditions. $P < 0.05$. Scale bars, 50 µm. One way-ordinary ANOVA with * $P < 0.05$, ** $P < 0.01$, *** $P < 0.005$. The exact p values are listed in **Appendix Table S5**.

Table S1 Differential gene expression in TNCKO TdLNs in comparison to WT TdLNs

Expression and p values of the genes differentialy expressed in TNCKO TdLNs in comparison to WT TdLNs as determined by RNA sequencing analysis with p value < 0.05. Data are deposited at the EMBL-EBI ArrayExrpress archive (E-MTAB-16360).

Table S1.1 TNCKO TdLNs vs WT TdLNs

**Gene name**	**log2 (FoldChange)**	**padj**
Gm12987	2.511966203	0.041167779
Sema3d	2.567876577	0.014276272
Gm37126	2.592280617	0.041167779
Ighv2-2	2.632829109	0.020735723
8030451A03Rik	2.709609155	0.042088513
Vwc2	2.72165088	0.020357857
Elfn2	2.731762113	0.04641008
Lyve1	2.797793007	0.011094218
Adra1b	2.828720367	0.005147109
Alpk3	3.043175694	0.037722793
Gm13868	3.049307321	0.024225547
Mmrn1	3.081743339	0.028507208
Igkv9-120	3.156997628	0.03396288
Igkv10-96	3.242494261	0.036807815
Ighv10-1	3.293828605	0.047130474
Igkv10-94	3.317565048	0.046705107
Cd5l	3.372442946	0.008102236
Igkv6-25	3.377964341	0.031061075
Igkv1-110	3.407651813	0.039613645
Clec4n	3.508842111	0.022263943
Igkv12-98	3.63722609	0.041826946
A130023I24Rik	3.66219486	0.0436441
Igkv4-79	3.673405716	0.029864011
Neb	3.678289573	0.020735723
Igkv10-95	3.756552702	0.04401888
Ighv1-58	3.856734247	0.017357758
Ighv9-3	3.998069033	0.038844683
Ighv1-7	4.072525674	0.013961075
Igkv4-86	4.208188449	0.030641479
Ccl8	4.232857541	0.003744706
Gm30648	4.291271862	0.046449898
Mybpc1	4.302796745	0.025112208
Cacna1s	4.339475354	0.017357758

Igkv6-17	4.397951382	0.024415161
Ighg2c	4.430442467	0.023281198
Ighv1-4	4.568696561	0.005147109
Ighv4-1	4.592402064	0.010983625
Gm13453	4.607759915	0.005452016
Igkv8-28	4.914650833	0.005147109
Igkv14-111	4.914975877	0.00962195
Ckmt2	5.201991649	0.030137594
Myh3	5.271677386	0.026430348
Gm47523	5.300097721	0.01425198
Gm44428	5.334805372	0.029703678
Sebox	5.418034693	0.016708274
Igkv5-45	5.464465711	0.016209472
Igkv14-126	5.540184388	0.004654508
Art1	5.786544269	0.045709433
Igkv11-125	5.864876769	0.034860387
Lrrc2	6.029266812	0.020739803
A930016O22Rik	6.265093315	0.034478029
Hotairm1	6.441699375	0.003744706
Jsrp1	6.453687282	0.018694338
Ckm	6.721148762	0.038610518
Tcap	6.826427953	0.033168524
Kcnj11	6.884147782	0.025771171
Myh2	8.416161373	0.02719909
Acta1	9.777444106	0.016851919
2310065F04Rik	18.48034991	0.00723355
Cav3	19.11940832	0.005232615
Eef1a2	23.96702039	0.00013999
Xirp2	25.4350714	2.656E-05
Gm13848	-23.13927738	6.372E-07
Klk1b5	-12.02669382	0.005147109
Gm31497	-9.874301897	0.028507208
Ttc22	-9.789559717	0.026277587
Sowaha	-9.493708605	0.023913406
Gm14130	-9.390506843	0.005232615
Olfr459	-9.237650161	0.005232615
Gm37427	-9.173565658	0.048649149
Dcdc5	-8.802442662	0.017019191
Itprid1	-8.51604231	0.023952601
Gm28401	-8.489791063	0.010983625
Gm18636	-8.451344218	0.019125517
AC132444.6	-8.451305831	0.011094218
Pgr	-8.182130001	0.005232615

Gm21083	-8.136007925	0.017019191
Gm37673	-8.130671855	0.033225513
Gm30251	-8.072061998	0.016851919
Gm42575	-8.06310539	0.016162143
Gm36346	-8.025759187	0.018694338
Fut9	-8.01086766	0.005232615
Slc28a3	-7.995815727	0.018918241
Gm16138	-7.990769404	0.020589312
Ly6g6c	-7.961366638	0.005147109
9130214F15Rik	-7.846888281	0.020589312
1700111E14Rik	-7.806165259	0.044241867
Gm15454	-7.79755515	0.041167779
Klk1	-7.732902952	0.010796781
Gm4963	-7.726482557	0.046568935
Gm43534	-7.713948347	0.020589312
Gm40999	-7.661058887	0.010796781
Prr27	-7.642167321	0.045864383
Gm8439	-7.627393215	0.010983625
Gm36945	-7.583896537	0.024933342
Gm28845	-7.579135703	0.015082811
Gm20515	-7.533033392	0.016895148
Gm48092	-7.524642607	0.016708274
Gm41333	-7.474842916	0.020589312
Acsm1	-7.455464825	0.040871261
Gm32358	-7.446791105	0.011820252
Sval2	-7.441316561	0.043302679
Gm20758	-7.392733666	0.016851919
Mir6362	-7.364901687	0.011193115
Gm26918	-7.236065072	0.010416256
9430085M18Rik	-7.207300793	0.005147109
Gm47832	-7.203717135	0.017727415
Gm37184	-7.122976889	0.034342169
Cftr	-7.121437793	0.005232615
Gm49028	-7.041075532	0.015457445
Klk1b11	-7.026317873	0.024933342
Tcfl5	-6.997771239	0.00962195

Table S1.2 TNCKO TdLNs vs WT TdLNs
(inflammatory and adaptive immune responses)

Gene name	log2 (FoldChange)	padj
Igkv6-23	2.060094431	0.135716743

Klrc2	1.866847764	0.282105464
Ighv5-16	3.269982074	0.136438119
Trbv13-1	2.087068105	0.18926946
Ighv7-3	3.860142387	0.083803887
C1ra	1.283167598	0.173520807
Emp2	-1.289348725	0.168713276
Batf3	0.317544759	0.886344282
Prdm1	1.117802797	0.217753876
Ighm	1.512306097	0.180492633
Il17f	3.615860703	0.148797525
Ctla4	1.818144921	0.302111123
Cd81	-1.173811793	0.217753876
Havcr2	1.239762723	0.123621124
Clec4a1	1.495769426	0.273799206
Jam3	0.755781065	0.232618652
Igkv3-4	2.590395887	0.131237621
Ighv2-9-1	0.777117541	0.792026673
Igkv3-10	2.755912605	0.093519428
Ighv14-1	3.066337367	0.11500285
Ighv5-9	3.236577544	0.086448295
C1rl	-3.559422562	0.049102116
C1rb	3.747613016	0.082732098
Cd7	1.601309114	0.142197237
Ighv1-72	2.203084167	0.088387379
Igkv6-17	4.397951382	0.024415161
Igkv5-39	2.780336021	0.077892106
Ighv6-3	4.474278246	0.082722342
Ighv1-18	1.772684791	0.167513685
Ighv1-61	1.900667592	0.295995218
Trav12-2	2.605275765	0.206580636
Ifnk	-1.182059361	0.308141746
Clec4n	3.508842111	0.022263943
Rora	-1.226237066	0.214615554
Ighv1-69	2.369196343	0.146445523
Fcer1g	1.156495434	0.130809238
Igkv4-72	2.969074859	0.085418535
Treml4	2.027479001	0.157426935
Nedd4	-1.443508712	0.172934123
Igkv6-25	3.377964341	0.031061075
Raet1e	-1.755473963	0.199252937
Jag1	-0.890201081	0.286109368

Myd88	0.57070605	0.205105788
Ighv1-76	1.738478548	0.114460061
Il18bp	0.1637887	0.876742243
Nlrp10	1.727264582	0.162633934
Ighv1-58	3.856734247	0.017357758
C1qa	1.302911915	0.172730554
Igkc	2.71812945	0.067504255
Trbv20	1.724837589	0.311617314
C1s2	-2.015236263	0.30966936
Ighv1-47	2.351233196	0.178322674
Rnf125	1.143583988	0.220300129
Igkv12-38	3.54323699	0.134403591
C6	2.622346623	0.196495141
Ighv5-6	2.350470303	0.117085389
Igkv16-104	2.657092932	0.100276052
Ighv1-78	2.058015783	0.31168129
Igkv6-32	1.533086997	0.213386109
C1qc	1.610577595	0.077429026
Ighv2-5	2.81743118	0.053273658
Igkv3-5	2.097423952	0.121760633
Otub1	0.336445094	0.26570892
Igkv10-94	3.317565048	0.046705107
H2-Aa	0.961139711	0.279684189
Ighv9-1	3.237979016	0.160321272
Ighv14-4	2.030219047	0.11921615
C3	1.516298903	0.116210434
Ighv9-3	3.998069033	0.038844683
Entpd7	-1.308662757	0.067858687
Igkv9-120	3.156997628	0.03396288
Ighv1-19	2.715117067	0.165101291
Mb	2.834808849	0.241950982
Cracr2a	-1.921282414	0.097918084
Ighv1-22	2.850804887	0.067918715
Igkv2-137	3.110738031	0.137216543
Trbv5	2.090686879	0.288707442
Igkv10-96	3.242494261	0.036807815
Ung	-0.939395383	0.29851765
Igkv1-117	2.737634802	0.138936856
Igkv2-109	2.58980865	0.106607565
Igkv4-74	2.344448715	0.232929953
Trgv2	2.04184972	0.283267467

Igkv14-100	2.784154149	0.169827859
Igkv8-24	4.072872704	0.124535813
Trpm4	-1.700837765	0.109265022
Igkv13-85	3.211007478	0.168227914
Igkv8-21	2.103661668	0.185050714
Mcoln2	0.98925485	0.199029178
Gm12990	3.015204482	0.168227914
Cd4	1.257127706	0.267522615
Trav16	1.486249795	0.267522615
C1qb	1.333626464	0.181349428
Jchain	1.936710012	0.158551673
Clec4a2	2.31221991	0.168138186
Gzmb	2.251607353	0.264571985
Ighv1-11	2.383967925	0.260406932
Marchf8	-1.520047271	0.275550236
Ighv10-1	3.293828605	0.047130474
Tarm1	2.093582267	0.223227068
Ighv3-8	2.437131225	0.145282657
Ighv5-9-1	2.099033256	0.253629053
Mpeg1	1.205405468	0.176950538
Mfsd6	-1.886789909	0.098112064
Pdcd1lg2	1.395987723	0.138129283
Trbv19	2.270866541	0.263535227
Ighv3-5	4.696108866	0.054468986
Ighg2c	4.430442467	0.023281198
Ighv1-81	2.730974426	0.07793831
Iglc2	1.373875467	0.231326066
Anxa1	-1.601583217	0.272783014
Serpinb9b	2.669977269	0.258023492
Ifng	3.267871042	0.287667569
Ighv1-66	2.747434728	0.205398723
Ifnar1	0.518456796	0.512411322
Ifnar2	0.12403608	0.86142948
Fam114a1	-1.362696967	0.130809238
Slc7a2	-3.910718134	0.050873476
Vnn1	-4.447793763	0.06023864
Pomgnt1	-1.251934423	0.075066604
Lrrc25	1.353640206	0.171402892
Tyrobp	0.964393663	0.221776837
Csf1r	1.134204787	0.131237621
Ccl7	2.84628275	0.165431132

Il17f	3.615860703	0.148797525
Pla2g3	-2.409834618	0.106750796
Casp7	-1.289799404	0.126832892
Cd68	0.7911457	0.240992906
Havcr2	1.239762723	0.123621124
Jam3	0.755781065	0.232618652
Cxcl9	2.426840578	0.258023492
Mal	-5.351686848	0.177571444
Chia1	-6.777023819	0.041735252
Mefv	1.791724776	0.177761923
Nupr1	-3.790631422	0.022263943
Saa3	4.423792357	0.150330847
Gnat2	-2.122885708	0.041167779
Smpdl3b	-3.166037305	0.053611748
Il23r	1.674024279	0.202241438
Nfix	-2.438962849	0.082567316
Agt	-5.093882591	0.118546845
Tlr2	0.935904281	0.258835596
Hpse	1.213543218	0.20188904
Serpinb1a	0.994210409	0.199029178
Il17rc	-1.596305852	0.169682616
Fcer1g	1.156495434	0.130809238
Serpina1b	1.698154699	0.207834507
Egfr	-1.315420633	0.299173238
Vcam1	1.135584218	0.268600145
Cxcr2	2.05190639	0.205398723
Ufl1	-0.93943122	0.172934123
Hyal1	-1.911807295	0.158428994
Myd88	0.57070605	0.205105788
Il31ra	2.37706897	0.161180117
Il18bp	0.1637887	0.876742243
Nlrp10	1.727264582	0.162633934
Pla2g2d	1.407508699	0.231936063
C1qa	1.302911915	0.172730554
Pla2g7	-2.2862543	0.128941532
Cd300a	0.950488387	0.257315141
Gpr15	2.90596576	0.196495141
Naip1	1.511439076	0.120262083
Cybb	1.15739914	0.273634695
Snca	-4.839158665	0.227817618
Nr4a1	-1.454044888	0.106607565

Il34	-2.327650768	0.11392492
Cx3cl1	-1.224048828	0.243899532
Smo	-1.571167724	0.292633837
Scyl1	-0.757954651	0.263535227
Alg3	-0.927808463	0.133945962
C3	1.516298903	0.116210434
Gna14	-1.161605622	0.190414485
Cebpb	-1.531873815	0.206411395
F12	1.647153187	0.159552467
Casp6	-1.07991334	0.231936063
Zfp941	1.569842874	0.396838823
Adamdec1	2.09160785	0.176617002
Thrb	-2.346395092	0.141931576
Nlrp9b	-5.035607983	0.089565358
Ccl19	2.623513567	0.161869829
Cxcr6	0.834798323	0.297349639
Il1rap	-1.273696765	0.162902192
Tnfrsf1b	1.145111728	0.291730891
Hmox1	1.702187244	0.171384835
Ifngr1	1.06058828	0.121462981
Il17re	-3.502028678	0.037380691
F2r	1.457635002	0.197353699
Elf3	-5.897147654	0.072593349
Pbxip1	-0.679991313	0.291788747
Serpina3n	2.276323533	0.196495141
Tfrc	-0.782547264	0.189101402
P2rx1	2.034831103	0.242835458
Pomt2	-1.197256333	0.20218044
Ccl12	2.72440139	0.086193977
Slc11a1	0.895556229	0.300768831
Odam	-6.741045436	0.022644205
P2rx7	-0.876566399	0.299048232
Epha2	-3.095428975	0.038241923
Gzmb	2.251607353	0.264571985
Cxcl10	1.924662327	0.106607565
Ptgir	0.930924666	0.089651665
Stk39	-2.366996113	0.046153392
Ccl3	2.584398254	0.220464882
Dab2ip	-1.64576505	0.249601881
Klk1b11	-7.026317873	0.024933342
Ano6	0.294258285	0.302612534

Il17d	-4.630830233	0.022133015
Adcy8	-6.275325056	0.0248165
Ccl28	-5.007994902	0.055174806
Lats1	-0.446218866	0.261434459
Mavs	-0.900345363	0.234608387
Cp	0.945395994	0.287631157
Zdhhc15	1.014823087	0.547153666
Il1f5	-4.408381039	0.212470217
Cmtm4	-2.353648446	0.085432396
Kit	-1.974174656	0.130146244
Anxa1	-1.601583217	0.272783014
Alox5ap	1.245170203	0.175944393
Casp3	1.15352974	0.167448857
F8	1.390147618	0.070274968
Zeb2	0.685861352	0.258003473
Ifng	3.267871042	0.287667569
Itgam	1.709764278	0.115313718

Table S2 Differential gene expression in WT FRCs in comparison to TNCKO FRCs

Expression and p values of the genes differentialy expressed in WT FRCs in comparison to TNCKO FRCs as determined by RNA sequencing analysis with p value < 0.05. Data are deposited at the EMBL-EBI ArrayExrpress archive (accession no E-MTAB-14801).

**Gene name**	**log2 (FoldChange)**	**padj**
Pdpn	0.9066	0.32465967
Ccl21 (Ckb9)	0.918584905	0.602453789
Vcam1	0.34537292	0.7461508
Col6a1	1.70150337	0.109207083
Ccl19 (Ckb11)	0.293943055	0,613416044
Col1a2	0.683499388	0.933790068
Col1a1	0.405388035	0.574245747
Acta2	0.788261593	0.533430249
Ltbr	0.202147496	0.831079757
Icam1	-6.021832898	0.002841558
Il7	-2.625790363	0.295134076
Igfbp4	-1.245117066	0.29552561
Il7	-2.625790363	0.295134076
Cxcl10	-6.772976388	0.236006461
Cxcl1	-0.570922899	0.945824603
Cxcl16	-2.715864475	0.521381362

Tgfb2	-0.242581639	0.94255552
Ccl5	-2.022505602	0.432339941
Il15	-2.969866695	0.287998067
Il6	-0.265916546	0.968089207
Igfbp3	-2.139929297	0.502956655
Ccl27a	-1.320227028	0.310626853
Il33	-4.21210021	0.006072829
Crp	-0.159696707	0.976510675
Tnfa (Tnfsf2)	-1.681406034	0.829677164
Il34	-0.119826095	0.976510675
Ccl25	-0.018259794	0.992838044
Il16	0.144489022	0.975575439
Ccl9	4.464008769	0.051145251
Ccl2	0.003586239	0.999307763
Hgf	1.048730604	0.646667667
Vegfa	0.960713505	0.345126123
Cxcl14	9.684574217	0.132596255
Adipoq	26.28602822	1.61e-10
Ccl7	1.430097504	0.528721404
Tgfb1	1.084297453	0.354767505
Ccl8	3.763700754	0.185308077
Tgfbi	3.625251642	0.152086568
Cxcl12	0.301928787	0.916853433
Lgals9	-0.752434186	0.288990611
Eif2ak2	-1.360615552	0.094921087
F2rl1	-3.841659033	0.023169835
Tlr2	-1.390095548	0.321701722
Egf1	-1,960461286	0.121843013
Ccl5	-2.022505602	0.432339941
Epha2	-1.051348615	0.17436972
Il33	-4.21210021	0.006072829
Cd24a	-8.255105481	0.001743409
Il7	-2.625790363	0.295134076
Tlr3	-2.556072186	0.020666461
Wnt5a	0.392162701	0.399524647
Csf1r	1.405081728	0.159563197
Apod	3.218643081	0.05747062
Snai2	0.638650028	0.355213351
Ffar2	1.933807649	0.32465967
Il17ra	0.799270997	0.15901213
Twist1	1.276393849	0.239292064
Adipoq	26.28602822	1.61e-10
Il1rl1	4.214144229	0.05747062

Lpl	3.161434253	0.089168476
Rel	-1.025996838	0.378344336
Isg15	-3.295043876	0.009973609
Irf7	-4.982330684	0.012718103
Stoml2	-0.857129051	0.301717494
Txk	-1.754646539	0.181038613
Spn	-2.734786632	0.009973609
Inava	-2.059321604	0.015493105
Runx3	-3.234719567	0.175944291
Gadd45b	-0.744995599	0.440039021
Lyn	-0.760310299	0.379913438
Kit	-8.642351023	0.160778438
Pcsk5	-4.116269896	0.048680318
Gbp5	-3.990967972	0.01938699
Tnfaip8	-1.341020009	0.382772181
F2rl1	-3.841659033	0.023169835
Trex1	-2.289907425	0.066002196
Rgcc	-4.221250551	0.148330764
Casp1	6.958609484	0.407489104
Fabp4	5.152788419	0.092264898
Kat2a	-0.807567863	0.367133179
Nod1	-1.682933597	0.090534737
Gata3	1.431834337	0.265647885
Irf9	1.627592456	0.079488545
Ephb6	3.389544468	0.031818849
Tlr2	1.390095548	0.321701722
Gpam	0.621859639	0.257674652
Zbtb7b	0.629449632	0.210136474
Cd59a	0.833568431	0.298280029
Gas6	2.285046779	0.343951042
Cd36	8.263129481	0.031025652
Agt	3.286630024	0.151014761

Table S3 Differential protein expression in FRCs in dependence of TNC and IR

Expression and p values of the proteins differentially expressed in WT FRCs in comparison to TNCKO FRCs and in dependence of IR as determined by mass spec analysis with p value < 0.05. Data are deposited at the EMBL-EBI ArrayExrpress archive (accession no at the PRIDE partner repository with the dataset identifier PXD060164).

Table S3.1 FRC WT NIR vs FRC TNCKO NIR

Gene name	logFC	P_Value
Nid1	1.25655397	0.0087878
Col5a1	4.82	1.54934E-09
Ero1a	3.49	4.15E-10
Cav1	2.25	7.36E-09
Eng	5.19	4.43811E-13
Lgals3	2.10262835	0.01426803
Adamts2	1.4	0.00104054
Col1a2	9.64	3.85E-15
Pdgfra	4.33	1.31E-07
Col12a1	2.3405077	0.03486441
P3h4	5.58	1.50E-11
Loxl3	3.3	1.41E-10
Mmp14	4.46	8.44E-10
Crtap	2.05	4.65E-07
Serpinh1	0.9	8.07E-06
Lox	3.64	4.22E-11
Col15a1	4.02	2.48E-05
Postn	6.66	4.79E-14
Mmp11	5.07	3.99E-14
Ddr2	6.92	2.01E-16
Hsd17b1	3.23	1.96251E-09
Flot1	0.211636	0.32368855
P3h1	3.28	4.77E-05
Cav2	1.99	0.00107278
Mmp2	7.01	4.96E-16
Aplp2	0.40334582	0.32726395
Myo1e	2.66	2.93E-06
Efemp2	4.1	3.13E-07
P4ha1	2.02	1.40E-09
Tcp1	-0.53139757	0.00514761
Col8a1	-0.35479269	0.05696238
Col14a1	-4.6356	4.01957E-10

Flrt2	-4.7238	2.55639E-11
Kpna1	1,3843	1.86893E-06
Larp1	1.03481161	0.0202512
Pafah1b1	0.22793755	0.31247988
Cops2	0.29835892	0.28276225
Rps6	1.16	8.83E-05
Gstp1	0.75	0.00231166
Gnb1	0.72801585	0.02311979
Prdx1	0.68	0.00216506
Prkcd	3.65	4.35E-08
Cdk1	2.84	3.27E-05
Impdh2	1.28	5.77E-05
Gnl3	1.43	0.00466183
Epha2	1.92	0.0002052
Purb	4.91	6.52E-09
Gba	3.02	2.11E-11
Ythdf2	2.39	1.30E-08
Gnai2	0.57	0.00052856
Mvp	1.51	2.46E-06
Txnrd1	1.64	2.71E-05
Stat6	1.52	0.00038429
Prpf19	1.28	6.04E-06
Pes1	2.09	0.00056149
Fubp1	2.67	5.46E-07
Cdk5rap3	2.27	1.23E-05
Mcm7	2.76	1.39E-07
Cxcl12	3.77	8.35E-09
Psmg1	1.28	0.0002308
Smad1	0.4619	0.004599501
Ccn3	3.34	0.00018798
Etv6	3.92	8.92E-12
Otud6b	1.2159	0.001648941
Prkar1a	0.36004737	0.27482184
Serpinb1a	2.59	6.17E-08
Bysl	0.37859199	0.43220622
Prdx2	1.73	6.12E-08
Pura	1.59	1.63E-06
Yme1l1	1.60368809	0.03843443
Dlg1	3.83	1.59E-07
Dab2	1.0799	0,004384278
Lipa	3.19	1.74E-06

Lrp1	4.94	1.06E-06
Ncstn	0.7	0.00032801
Cd151	-2.19	1.86E-06
Kif1a	1.31	0.00052711
Larp7	-0.83554952	0.01131989
Ddr3	-6.92	2.01E-16
Sidt2	-1.07	0.00013116
Pdgfrb	-2.45099623	0.04332009
Npr3	-3.28	2.98813E-09
Dbn1	-1.06	1.34E-05
Hprt1	-3	4.74E-07
Plxnb2	-1.84	4.24E-07
Serpinh1	0.9	8.07E-06
Ddr2	6.92	2.01E-16
Crtap	2.05	4.65E-07
Col5a1	4.82	1.55E-09
Adamts2	1.4	0.00104054
P4ha1	2.02	1.40E-09
Strap	1.33	0.00048527
Vasn	1.2187029	0.03107797
Lox	3.64	4.22E-11
Tcp1	0.53139757	0.00514761
Hsp90aa1	0,65	0,001119881
Tgfb2	1.3	8.04E-05
Fbn1	1.45	0.00033005
Cav1	2.25	7.36E-09
Htra1	2.27	3,19698E-09
Cd109	4.98	3.54E-10
Thbs1	8.67	4.93E-16
Got1	2.77	1.53E-08
Dnm2	0.00575385	0.97626355
Itga3	-6.89	2.01877E-12
Mpp5	-1.09	0.00189969
Snx1	-1.24019313	0.03143405

Table S3.2. FRC WT IR vs NIR

Gene name	logFC	P Value
Rhoa	0.46734991	0.40846868
Vkorc1	0.96389137	0.09372388

Gnb1	0.24066404	0.53679014
Ndufs4	0.93630231	0.2190456
Fam162a	1.65	0.00907001
Macrod1	0.2154367	0.72553395
Rpl30	1.46	0.00678679
Stxbp1	0.2105802	0.73203121
Cdk5 (Crk6)	0.58329804	0.62984251
Samhd1	0.25734939	0.77848329
Ddx21	0.71508945	0.02805155
Mcm6	1.85	3.33E-05
Rps19	2.29	5.35E-06
Cxcl12	1.42	0.0001391
Mif	2.54	0.00037409
Hmgb1	3.42	1.15E-07
Mrps27	1.03	0.000254888
Mcm3	2.12	9.98E-05
Mcm5	1.63	1.52E-05
Zyx	1	0.00076839
Mcm4	2.47	5.63E-07
Asns	1.44	0.00020161
Clec2d	1.79	1.16E-06
Nono	0.06215871	0.76066853
Pdgfrb	0.26941214	0.28954524
Colec12	1.16	0.00040782
F3	2.01	0.00010822
Mgst1	0.00679346	0.96363743
Sod2	0.5502238	0.0173167
Ube2n	2.67	0.00015748
Rbm14	0.77684232	0.02965056
C1qbp	0.38614221	0.08233306
Cdc42	1.07	2.32E-05
Col3a1	4.28	2.96E-07
Dnaja2	0.66293194	0.05529391
Gstp1	1.01	5.90E-05
Rbm3	1.2	0.00240381
Sqstm1	0.81	0.00188574
Cfl1	1.18	0.00128202
Prdx1	0.93	0.00081993
Mapk1	1.31	0.00226913
Rac1	1.22515284	0.0265266
Prdx5	3,67	8.091E-06

Ipo7	1.13	0.00081783
Dbi	1.57	0.00054805
Sec61b	2.88	0.00037676
Cnpy3	2.26	0.00048108
Cst3	2.62	1.76E-06
Oxsr1	1.39	0.00011428
Hsp90aa1	0.96	8.08E-05
Hsp90ab1	0.7	0.00095471
Dpysl3	1.07	0.00014194
Otub1	1.38	0.00095902
Spr	1.64	0.00056102
Mcm7	3	1.77E-09
Ubqln2	1.17	2.02E-05
Pcna	2.94	8.74E-05
Lox	0.76	0.00016952
Uba1	0.81	0.00035936
Ddx3x	2.4	6.00E-06
Ywhaz	1.05	2.81E-05
Ide	0.82	0.000254359
Mcm2	1.83	7.99E-05
Cnn2	1.67	0.00033997
Ggt5	3.16	0.000490244
Hnrnpd	1.29	0.000387103
Dpep1	1.55	0.00051699
Irgm1	-0.98	0.00081527
Epha2	-1.98	6.39E-05
Serpinb1a	-1.05	0.00014934
Poldip2	-1.18	0.00300716
Tapbp	-2.06	0.000448726
Tap1	-1.7	1.76E-06
Aldh3a2	-1.37	1.73E-06
Ptprz1 (Hptpz)	0.59	0.003400374
Fth1	0.38173893	0.07945144
Gstp1	1.01	5.90E-05
Rps3a	0,72	0.004536503
Sod2	1.27	0.000105348
Mif	2.54	0.00037409
Tkt	1.17	4.65E-05
Ndufs4	0.93630231	0.2190456
Pdgfrb	0.26941214	0.28954524
Serpine1	-0.29698804	0.15042892

B4galt7	-1	0.00015715
Tcp1	0.63	0.00490854
Tkt	1.17	4.65E-05
Crtap	-0.13765949	0.56691166
P3h4	0.18826537	0.57238582
Flot1	-0.25129768	0.18833448
Postn	0.64	0.00094707
Ddr2	0.63	0.00345901
Serpinh1	0.02466483	0.86378546
Lox	0.76	0.00016952
P4ha1	0.08733152	0.55827607
Ero1a	0.0696698	0.64698749
Mmp14	0.54291001	0.01461059
Mmp11	1.44364716	0.01980396
Lgals3	0.58058339	0.04068826
Mmp2	0.49	0.00901394
Col1a2	0.7	0.00493934
Tnc	0.356591	0.51340939
Cav1	0.13695505	0.3727634
Myo1e	0.33974737	0.47637916
Loxl2	0.35445031	0.22989815
P3h1	0.54043408	0.13121827
Nid1	0.06380708	0.82801393
Col5a1	0.617897	0.11556653
Emilin1	0.11354898	0.72112982
Col1a1	0.84245017	0.0347062
Col15a1	0.07897689	0.90820059
Col12a1	0.28945392	0.72305869
Pdgfra	0.85791672	0.08353464
Ccn2	0.30836172	0.55568145
Col8a1	0.31532387	0.06394914
Efemp2	-0.12619315	0.72648919
Loxl3	-0.46876663	0.0338956
Stxbp1 (Munc18-1)	-0.51	0.001813249
Acsl1	0.33769687	0.24409524
Bcat2	0.08054628	0.6601347
F3	2.01	0.00010822
Csnk2b	1.84	0.00556816
Hmgb1	3.42	1.15E-07
Il6st	1.84	8.56E-05
Mcm2	1.83	7.99E-05

Rpl3	1.02	0.00017467
Oxsr1	1.39	0.00011428
Hsp90ab	0.70	0.000954713
Zyx	1	0.00076839
Stip1	1.01	0.00032317
Tfrc	1.62	0.00066124
Lox	0.76	0.00016952
Cdc42	1.07	2.32E-05
Col3a1	4.28	2.96E-07
Ybx1	0.299501	0.08671835
Mapk1	1.31	0.00226913
Impdh2	2.11	0.00019303
Cfl1	1.18	0.00128202
Rplp0	0.95	2.18E-05
Fkbp1a	2.01	0.00019824
Cxcl12	1.42	0.0001391
Ubtf	1.21	0.00020596
Cacybp	1.44	0.00025378
Eef1e1	2.59	3.65796E-07
Serpine1	0.29698804	0.15042892
Asah1	0.32301578	0.08570793
Irgm1	-0.98	0.00081527
Mapk1	1.31	0.00226913
Rac1	1.22515284	0.0265266
Cxcl12	1.42	0.0001391
C1qbp	0.38614221	0.08233306
Thy1	1.25	4.76E-05
Mif	2.54	0.00037409
Cd99 (Mic2)	1.14	0.00479148
Oxsr1	1.39	0.00011428
Swap70	2.48	1.14E-06
Spr	1.64	0.00056102
Serpine1	0.29698804	0.15042892
Mpp1	0.41102678	0.12788852
Ano6	-0.30183702	0.32596197

Table S3.3 FRC WT IR vs FRC TNCKO IR

Gene name	logFC	P Value
Sfrp1	-4.17	2.48E-10

Isg15	-2.79	5.26E-05
Bcat2	-2.21	1.43E-07
Tollip	-1.65	3.20E-05
Gsn	-0.71	0.00511165
Myo1c	-0.45	0.00416351
Asah1	-1.73	4.24E-07
Cxcl12	2.07	1.48E-09
Bclaf1	3.07	2.62E-06
Ctbp2	2.31	2.42E-05
Gas6	0.42	0.49199572
Zc3h15	1.37	0.00014258
Cdk9	0.98	2.34279E-05
Entpd2	1.74	4.73E-08
Rpl3	1.62	1.08E-05
Mcm2	5.47	2.02E-09
Hsp90ab1	0.85	0.000549904
Ifi204	1.29	0.001341586
Dapk3	1.21	5.72E-05
Smarca5	4.33	2.13E-06
Kars1	1.01	0.00029804
Gba	3.09	5.00E-10
Lox	2.81	5.73E-10
Rplp0	0.37	0.03344401
Actn4	0.75	0.00033299
Srsf3	0.08	0.9225257
Dbn1	0.69	0.00306163
Rps3	1.07	3.29E-05
Upf1	2.27	3.30E-08
Ncl	1.07	1.94E-05
Tfrc	4.97	8.75E-10
Mapk1	0.25	0.57796411
Atic	1.4	8.79E-05
Oxsr1	1.98	1.49E-06
Ifitm3	0.12	0.84266258
Alad	3.32	3.31879E-09
Trex1	1.85	7.21264E-05
Tgtp1	3.51	1.57573E-07
Stat2	1.90	2.89369E-06
Il1rap	2.92	5.92E-10
Ybx3	2.05	5.10E-07
Crkl	0.21	0.46948913

Sting1	0.63	0.0058375
Ybx1	1.66	9.06E-08
Irgm1	0.09	0.55491096
Erbin	2.48	1.95E-09
Ndufa13	2.37	0.00025289
Adar	1.94	1.27252E-05
Stat1	3.6	1.91E-09
Nmi	4.51	8.76759E-13
Iigp1	6.99	1.0626E-15
Ifit1	2.46	0.00043826
Pml	1.03	0.00025181
Eef1e1	1.08	1.90E-05
Jak1	3.67	1.23E-09
Nrp2	2.73	9.21E-05
Prkca	1.40	0.004123093
Ifit3	3.97	1.93896E-10
Impdh2	2.85	1.35E-05
Syncrip	2.11	4.93E-08
Eif4a2	1.89	0.00081529
Kif5b	1.94	3.21E-05
Gbp2	3,59	5.41146E-10
Anxa1	1.99	7.86E-08
Oas2	1.08	0.002870635
Usp10	1.47	3.92E-05
Gbp4	2.85	2.2778E-07
Ifit2	2.50	4.48639E-05
Smpd4	0.83515082	0.0484886
Zyx	2.47	6.25526E-09
Eif2ak2	2.54	2.44E-08
Stip1	1.17	0.00013831
Mat2a	0.86455759	0.00852482
Cacybp	1.85	5.31E-05
Flnb	1.65	0.00154126
Cad	0.90304897	0.04147857
Eprs1	0.65507942	0.11847934
Pdia3	0.76	8.25E-05
Rab12	-1.84	0.00015204
Rab11fip5	0.42915921	0.38414977
Ggt7	0.78455726	0.30103674
Shmt2	0.85	5.70E-05
Cox6a1	-2.80	0.000346801

Ndufa8	-0.54791588	0.03381228
Sco2	-3,67	2.15765E-06
Mtnd2	-0.54	0.004140451
Cycs	-0.71834406	0.01427243
Ndufc2	-0.101576	0.61944516
Cox5a	1.25305346	0.0345687
Mdh1	-1.83272413	0.00825116
Mybbp1a	-1.98751495	0.01258872
Adsl	-1.8	0.00035699
Aco1	-2.42	3.78E-07
Dlst	-0.42209797	0.04243473
Idh2	-0.67	0.00041874
Sdha	-0.08887283	0.52308894
Suclg1	0.88	0.00012556
Cat	-1.44	1.07E-05
Sucla2	0.95	8.62E-05
Slc37a2	0.87	0.00072168
Ndufb6	1.07049324	0.01579918
Cav1	1.66	1.09E-06
Ero1a	3.45	1.04E-09
Col1a2	8.66	2.08E-17
Lox	2.81	5.73E-10
Serpinh1	0.69	0.00013058
Flot1	0.03163443	0.87938753
P3h4	5.11	3.95E-11
Mmp14	3.86	7.80E-07
Crtap	2.54	3.02E-06
Ccn2	3.06	9.17E-07
Ddr2	6.01	4.83E-14
Tnc	2.92	1.37E-05
P3h1	3.8	1.45E-05
Mmp11	3.35	3.64E-05
Lgals3	2.31	0.0016581
Pdgfra	3.19	1.81E-08
Col15a1	3.66	4.32E-08
Col12a1	1.12907197	0.10516776
Nid1	0.82	0.00415414
Col5a1	3.92	1.01E-09
Eng	5.56	3.57023E-14
Loxl3	3.48	2.86E-12
Emilin1	5.01	5.47E-13

Postn	5.32	5.27E-12
Mmp2	6.24	8.37E-15
Tkt	1.69	5.84E-07
Efemp2	4.21	3.60E-13
Myo1e	2.13	0.0002141
Col1a1	0,98	1.98
Tcp1	-0.74	0.00244475
Col8a1	-0.41761062	0.02041826
Flrt2	-4.81	1.73763E-12
Loxl2	-1.15	1.87508E-05
Col14a1	-4.33	1.55902E-09
Ccn1	-1.91	0.002536102
P4ha1	0,60	0.002285728

Table S4 Cox regression analysis for HNSCC RT cohort (TNC-high)

Parameters	n (%) mean (SD)/median (IQR)	Univariate p value	Hazard Ratio (HR)	LowerCI	HigherCI	Multivariate p value	HR	LowerCI	HigherCI
**TNC**	148.69 (96.55-236.61)	0.617	1	0.999	1.002				
**PDPN**	48.88 (32.81-72.61)	0.886	0.999	0.991	1.008				
**CCL21**	15.86 (5.52-46.34)	0.507	1.001	0.998	1.004				
**CCL19**	4.33 (1.47-12.15)	0.416	1.002	0.998	1.005				
**VCAM1**	2.25 (0.99-5.33)	0.941	0.999	0.981	1.018				
**COL1A1**	523.04 (243.87-1328.81)	0.571	1	1	1				
**COL6A1**	158.45 (82.59-291.30)	0.511	0.999	0.998	1.001				
**ACTA2**	11.45 (6.15-21.51)	0.258	1.006	0.995	1.018				
**LTBR**	29.00 (24.07-36.54)	0.586	0.994	0.972	1.016				
**Age**	59 (51-67)	0.287	1.015	0.988	1.042				
**Gender**									
Male	104 (78.20)		ref						
Female	29 (21.80)	0.939	0.973	0.482	1.966				
**Tabacco amount**									
0	1 (0.75)		ref						
1	26 (19.55)	0.912	7608.951	0	1.11E+73				
2	52 (39.10)		13494.747	0	1.97E+73				
3	16 (12.03)		8361.312	0	1.22E+73				
4	37 (27.82)		17518.341	0	2.56E+73				
5	1 (0.75)		1.433	0	2.08E+150				
**Grade**									
1	14 (10.53)		ref						
2	76 (57.14)	0.206	2.529	0.6	10.656				
3	38 (28.57)	0.189	2.689	0.615	11.758				

4	2 (1.50)	0.979	0	0	0
x	3 (2.26)	0.434	2.611	0.236	28.83
**Pathological stage**					
1	5 (3.76)		ref		
2	8 (6.01)	0.903	3762.336	0	2.33E+61
3	17 (12.78)	0.895	7894.013	0	4.87E+61
4a	83 (62.41)	0.893	9023.291	0	5.55E+61
4b	3 (2.26)	0.883	22340.409	0	1.38E+62
x	17 (12.78)	0.885	625.184	0	1.09E+62

(continued)

**Clinical stage**					
1	2 (1.50)		ref		
2	14 (10.53)	0.919	1417.294	0	3.71E+63
3	21 (15.79)	0.911	2765.219	0	7.22E+63
4a	86 (64.66)	0.907	3840.715	0	1.00E+64
4b	3 (2.26)	0.909	3374.122	0	8.91E+63
4c	3 (2.26)	0.908	3658.352	0	9.66E+63
x	4 (3.01)	1	0.996	0	1.96E+72
**T stage**					
1	11 (8.27)		ref		
2	25 (18.80)	0.311	2.963	0.362	24.269
3	32 (24.06)	0.161	4.299	0.56	33.013
4	4 (3.01)	0.476	2.744	0.171	43.94
4a	43 (32.33)	0.19	3.875	0.51	29.415
4b	2 (1.50)	0.034	13.583	1.223	150.824
x	16 (12.03)	0.056	7.585	0.948	60.699
**N stage**					
0	35 (26.32)		ref		
1	14 (10.53)	0.876	1.114	0.287	4.318

2	3 (2.26)	0.595	1.767	0.217	14.414				
2a	2 (1.50)	0.317	2.916	0.358	23.778				
2b	41 (30.83)	0.102	2.112	0.863	5.169				
2c	18 (13.53)	0.001	4.824	1.858	12.527				
3	1 (0.75)	0.977	0	0	2.49E+281				
x	19 (14.29)	0.103	2.347	0.841	6.554				
**Alcohol history**									
Never	33 (24.81)		ref						
Yes	100 (75.19)	0.286	1.488	0.717	3.088				
**FRC marker**						0.042			
Low	66 (49.62)		ref				ref		
High	67 (50.38)	0.042	1.863	1.022	3.395	0.042	1.86	1.022	3.395

Table S5 p values

**Main Figures**		
**Figure**	**Group**	**p value**
1A	WT vs KO	0.0181
	WT NIR vs WT IR	0.037
	KO NIR vs KO IR	0.9985
	WT NIR vs KO IR	0.0257
	WT IR vs KO IR	0.9981
1C	WT vs KO	>0.9999
	WT NIR vs WT IR	0.001
	KO NIR vs KO IR	>0.9999
	WT IR vs KO IR	0.9379
1D	WT vs KO	<0.0001
	WT NIR vs WT IR	0.0436
	KO NIR vs KO IR	0.9959
	WT IR vs KO IR	<0.0001
1E	WT vs KO	0.048
	WT NIR vs WT IR	0.0246
	KO NIR vs KO IR	0.9996
	WT IR vs KO IR	0.0465
1F	WT vs KO	<0.0001
	WT NIR vs WT IR	0.0594
	KO NIR vs KO IR	0.9863
	WT IR vs KO IR	<0.0001
1G	WT vs KO	0.0272
	WT NIR vs WT IR	0.0111
	KO NIR vs KO IR	>0.9999
	WT IR vs KO IR	<0.0001
1H	WT vs KO	<0.0001
	WT NIR vs WT IR	0.0055
	KO NIR vs KO IR	0.9851
	WT IR vs KO IR	<0.0001
1K	WT vs KO (B220)	0.0159
	WT vs KO (CD3)	0.0317
2F	WT Ctrl vs WT + TNC	0.0004
	WT Ctrl vs KO Ctrl	0.0012
	WT + TNC vs KO + TNC	0.507
	KO Ctrl vs KO + TNC	0.8945

3B	WT vs KO	<0.0001
3D	WT vs KO	0.0486
	WT NIR vs WT IR	0.5147
	KO NIR vs KO IR	0.9935
	WT IR vs KO IR	0.275
3F	WT vs KO	<0.0001
	WT vs WT + α- CCR7	<0.0001
	WT vs WT + Nb3	0.0002
	WT vs WT IR	0.0001
	WT IR vs KO IR	<0.0001
	WT IR vs WT IR + α-CCR7	<0.0001
	WT IR vs WT IR + Nb3	<0.0001
3G	No FBS NIR vs TGFβ NIR	<0.0001
	No FBS NIR vs OSCC-CM NIR	<0.0001
	No FBS NIR vs OSCC-CM/GW NIR	0.4003
	OSCC-CM NIR vs OSCC-CM/GW NIR	<0.0001
	No FBS IR vs TGFβ IR	<0.0001
	No FBS IR vs OSCC-CM IR	0.0001
	No FBS IR vs OSCC-CM/GW IR	0.0002
	OSCC-CM IR vs OSCC-CM/GW IR	<0.0001
	No FBS NIR vs IR	<0.0001
	TGFβ NIR vs IR	<0.0001
	OSCC-CM NIR vs IR	0.0004
	OSCC-CM/GW NIR vs IR	0.999
3H	No FBS NIR vs TGFβ NIR	<0.0001
	No FBS NIR vs OSCC-CM NIR	0.0007
	No FBS NIR vs OSCC-CM/GW NIR	>0.9999
	OSCC-CM NIR vs OSCC-CM/GW NIR	0.0008
	No FBS IR vs TGFβ IR	<0.0001
	No FBS IR vs OSCC-CM IR	0.7623
	No FBS IR vs OSCC-CM/GW IR	<0.0001
	OSCC-CM IR vs OSCC-CM/GW IR	<0.0001
	No FBS NIR vs IR	<0.0001
	TGFβ NIR vs IR	<0.0001
	OSCC-CM NIR vs IR	0.0016
	OSCC-CM/GW NIR vs IR	0.4509
4B	shC NIR vs shT NIR	0.5503
	shC NIR vs KO NIR	0.4654
	shC NIR vs shC IR	<0.0001

	shC NIR vs shT IR	0.7733
	shC NIR vs KO IR	0.9382
4D	shC NIR vs IR	<0.0001
	shT NIR vs IR	0.0677
	KO NIR vs IR	0.0953
4E	shC NIR vs shT NIR	0.6281
	shC NIR vs KO NIR	0.5544
	shC NIR vs shC IR	0.0001
	shC NIR vs shT IR	0.0516
	shC NIR vs KO IR	0.2038
4F	shC NIR vs shT NIR	0.9943
	shC NIR vs KO NIR	0.5666
	shC NIR vs shC IR	<0.0001
	shC NIR vs shT IR	0.0079
	shC NIR vs KO IR	0.1367
4H	NIR vs IR	0.001
	IR vs IR + GW	0.0002
	NIR vs IR+ GW	0.4818
4I	NIR vs IR	0.0002
	IR vs IR + GW	0.0076
	NIR vs IR+ GW	<0.0001
5C	Ccl21 OSCC vs OSCC+ FRC	0.0002
	Ccr7 OSCC vs OSCC+ FRC	<0.0001
	Tgfβ1 OSCC vs OSCC+ FRC	<0.0001
	Il-10 OSCC vs OSCC+ FRC	0.0015
	Il-7 OSCC vs OSCC+ FRC	>0.9999
	Il-17 OSCC vs OSCC+ FRC	0.0012
	Acta2 OSCC vs OSCC+ FRC	<0.0001
	Tnc OSCC vs OSCC+ FRC	0.0011
	Col1a2 OSCC vs OSCC+ FRC	0.0001
	Cd206 OSCC vs OSCC+ FRC	0.9837
	Foxp3 OSCC vs OSCC+ FRC	0.534
	Ctla4 OSCC vs OSCC+ FRC	>0.9999
5D	Day1 OSCC vs OSCC + FRC	>0.9999
	Day1 OSCC vs OSCC + FRC KO	>0.9999
	Day1 OSCC+FRC vs OSCC + FRC KO	>0.9999
	Day8 OSCC vs OSCC + FRC	0.004623929
	Day8 OSCC vs OSCC + FRC KO	0.015315977
	Day8 OSCC+FRC vs OSCC + FRC KO	0.050026019

	Day15 OSCC vs OSCC + FRC	0.000726622
	Day15 OSCC vs OSCC + FRC KO	5.24083E-05
	Day15 OSCC+FRC vs OSCC + FRC KO	0.056366275
	Day18 OSCC vs OSCC + FRC	0.000325007
	Day18 OSCC vs OSCC + FRC KO	0.000836047
	Day18 OSCC+FRC vs OSCC + FRC KO	0.076750028
6B	NIR vs NIR + MP5	0.4786
	NIR vs IR	0.0087
	NIR vs IR + MP5	0.001
	IR vs IR+ MP5	0.0591
6D	NIR vs NIR + MP5	0.0456
	NIR vs IR	0.0039
	NIR vs IR + MP5	0.0011
	IR vs IR+ MP5	0.03
6F	NIR vs IR	0.0021
	NIR vs IR + MP5	0.8608
	IR vs IR+MP5	0.0013
7A	TNC high	0.039
7B	TNC low	0.08
**Extended view Figures**		
**Figure**	**Group**	**p value**
EV1B	WT vs KO	0.0366
	WT NIR vs WT IR	0.0055
	KO NIR vs KO IR	0.6333
	WT IR vs KO IR	0.3239
EV1E	WT vs KO	0.0094
	WT NIR vs WT IR	0.6905
	KO NIR vs KO IR	0.9975
	WT IR vs KO IR	0.0006
EV3E	WT vs KO	>0.9999
	WT NIR vs WT IR	0.017
	KO NIR vs KO IR	>0.9999
	WT IR vs KO IR	0.0179
EV1F	WT vs KO	0.067
	WT NIR vs WT IR	0.0127
	KO NIR vs KO IR	0.9241
	WT IR vs KO IR	0.0006
EV1G	WT vs KO	0.0723
	WT NIR vs WT IR	0.0103

	KO NIR vs KO IR	>0.9999
	WT IR vs KO IR	<0.0001
EV3G	No FBS NIR vs TGFβ NIR	<0.0001
	No FBS NIR vs OSCC-CM NIR	<0.0001
	No FBS NIR vs OSCC-CM/GW NIR	0.4538
	OSCC-CM NIR vs OSCC-CM/GW NIR	0.0059
	No FBS IR vs TGFβ IR	<0.0001
	No FBS IR vs OSCC-CM IR	0.8758
	No FBS IR vs OSCC-CM/GW IR	0.0017
	OSCC-CM IR vs OSCC-CM/GW IR	0.0001
	No FBS NIR vs IR	<0.0001
	TGFβ NIR vs IR	0.0004
	OSCC-CM NIR vs IR	0.7653
	OSCC-CM/GW NIR vs IR	>0.9999
EV3H	No FBS NIR vs TGFβ NIR	0.0026
	No FBS NIR vs OSCC-CM NIR	<0.0001
	No FBS NIR vs OSCC-CM/GW NIR	0.0317
	OSCC-CM NIR vs OSCC-CM/GW NIR	0.0042
	No FBS IR vs TGFβ IR	<0.0001
	No FBS IR vs OSCC-CM IR	<0.0001
	No FBS IR vs OSCC-CM/GW IR	0.0023
	OSCC-CM IR vs OSCC-CM/GW IR	<0.0001
	No FBS NIR vs IR	0.0306
	TGFβ NIR vs IR	0.0004
	OSCC-CM NIR vs IR	<0.0001
	OSCC-CM/GW NIR vs IR	0.0021
EV4D	shC NIR vs shT NIR	0.8354
	shC NIR vs KO NIR	0.916
	shC NIR vs shC IR	<0.0001
	shC NIR vs shT IR	0.0011
	shC NIR vs KO IR	0.1521
EV4E	shC NIR vs shT NIR	0.9996
	shC NIR vs KO NIR	0.8909
	shC NIR vs shC IR	<0.0001
	shC NIR vs shT IR	>0.9999
	shC NIR vs KO IR	0.9787
EV4F	shC NIR vs shT NIR	0.982
	shC NIR vs KO NIR	0.9995
	shC NIR vs shC IR	<0.0001

	shC NIR vs shT IR	0.1796
	shC NIR vs KO IR	0.919
EV4H	shC NIR vs shT NIR	>0.9999
	shC NIR vs KO NIR	0.3737
	shC NIR vs shC IR	0.001
	shC NIR vs shT IR	0.8021
	shC NIR vs KO IR	>0.9999
EV4G	shC NIR vs shT NIR	>0.9999
	shC NIR vs KO NIR	>0.9999
	shC NIR vs shC IR	<0.0001
	shC NIR vs shT IR	<0.0001
	shC NIR vs KO IR	0.0003
EV4I	shC NIR vs shT NIR	0.7047
	shC NIR vs KO NIR	0.9898
	shC NIR vs shC IR	<0.0001
	shC NIR vs shT IR	0.985
	shC NIR vs KO IR	0.0816
EV6A	TNC vs MP5	0.0305
	TNC vs Cy5-MP5	0.0255
	MP5 vs Cy5-MP5	0.9873
EV6B	MP5 vs Cy5-MP5	0.1765
**Appendix Figures**		
**Figure**	**Group**	**p value**
S1C	WT vs KO	0.4
S1D	WT vs KO	0.6804
	WT NIR vs WT IR	0.1881
	KO NIR vs KO IR	0.9994
	WT IR vs KO IR	0.0325
S1E	WT vs KO	0.0372
	WT NIR vs WT IR	0.7364
	KO NIR vs KO IR	0.2483
	WT IR vs KO IR	0.0022
S1F	WT vs KO	0.9833
	WT NIR vs WT IR	0.0132
	KO NIR vs KO IR	0.9979
	WT IR vs KO IR	0.0143
S1G	WT vs KO	0.9932
	WT NIR vs WT IR	0.0159
	KO NIR vs KO IR	0.9723

	WT IR vs KO IR	0.0023
S1H	WT vs KO	0.0889
	WT NIR vs WT IR	0.0103
	KO NIR vs KO IR	>0.9999
	WT IR vs WT IR	<0.0001
S1I	WT vs KO	0.003
	WT NIR vs WT IR	0.9988
	KO NIR vs KO IR	0.944
	WT IR vs KO IR	0.0032
S1J	WT vs KO	0.1943
	WT NIR vs WT IR	0.0446
	KO NIR vs KO IR	0.0476
	WT IR vs KO IR	0.2159
S1K	WT vs KO	0.9078
	WT NIR vs WT IR	0.9862
	KO NIR vs KO IR	0.0303
	WT IR vs KO IR	0.937
S1L	WT vs KO	0.9852
	WT NIR vs WT IR	0.9123
	KO NIR vs KO IR	0.9998
	WT IR vs KO IR	0.9784
S1M	WT vs KO	0.8857
	WT NIR vs WT IR	0.9796
	KO NIR vs KO IR	0.0214
	WT IR vs KO IR	0.0344
S1N	WT vs KO	0.6906
	WT NIR vs WT IR	0.9987
	KO NIR vs KO IR	0.2132
	WT IR vs KO IR	0.0241
S1O	WT vs KO	0.0779
	WT NIR vs WT IR	0.9511
	KO NIR vs KO IR	0.993
	WT IR vs KO IR	0.2911
S1Q	WT vs KO	0.8874
	WT NIR vs WT IR	0.9611
	KO NIR vs KO IR	0.9957
	WT IR vs KO IR	0.7625
S2E	WT Ctrl vs WT + TNC	<0.0001
	WT Ctrl vs KO Ctrl	<0.0001

	WT + TNC vs KO + TNC	0.8729
	KO Ctrl vs KO + TNC	0.896
S2F	WT vs KO	>0.9999
	WT NIR vs WT IR	0.7805
	KO NIR vs KO IR	0.9871
	WT IR vs KO IR	0.5516
S3B	WT vs KO	0.0706
	WT NIR vs WT IR	<0.0001
	KO NIR vs KO IR	<0.0001
	WT IR vs KO IR	<0.0001
S3J	Lox NIR vs IR	0.228571
	TGM2 NIR vs IR	0.771429
	MMP11 NIR vs IR	0.228571
	MMP2 NIR vs IR	0.771429
	MMP14 NIR vs IR	0.228571
S3K	Lox NIR vs IR	0.002165
	TGM2 NIR vs IR	0.002165
	MMP11 NIR vs IR	0.002165
	MMP2 NIR vs IR	0.002165
	MMP14 NIR vs IR	0.002165
S3M	No FBS FRC WT vs No FBS FRC KO	0.001
	No FBS NIR vs TGFβ NIR	0.0107
	No FBS NIR vs OSCC-CM NIR	0.412
	No FBS NIR vs OSCC-CM/GW NIR	0.9697
	OSCC-CM NIR vs OSCC-CM/GW NIR	0.1989
	No FBS IR vs TGFβ IR	0.1646
	No FBS IR vs OSCC-CM IR	0.3244
	No FBS IR vs OSCC-CM/GW IR	0.1568
	OSCC-CM IR vs OSCC-CM/GW IR	0.9764
	No FBS NIR vs IR	0.0008
	TGFβ NIR vs IR	0.0234
	OSCC-CM NIR vs IR	0.0013
	OSCC-CM/GW NIR vs IR	0.5057
S3N	No FBS FRC WT vs No FBS FRC KO	0.0036
	No FBS NIR vs TGFβ NIR	0.0037
	No FBS NIR vs OSCC-CM NIR	0.4403
	No FBS NIR vs OSCC-CM/GW NIR	0.3818
	OSCC-CM NIR vs OSCC-CM/GW NIR	0.9996
	No FBS IR vs TGFβ IR	0.8587

	No FBS IR vs OSCC-CM IR	0.9069
	No FBS IR vs OSCC-CM/GW IR	0.7934
	OSCC-CM IR vs OSCC-CM/GW IR	0.9945
	No FBS NIR vs IR	0.9142
	TGFβ NIR vs IR	0.1639
	OSCC-CM NIR vs IR	>0.9999
	OSCC-CM/GW NIR vs IR	>0.9999
S3O	No FBS FRC WT vs No FBS FRC KO	0.0009
	No FBS NIR vs TGFβ NIR	<0.0001
	No FBS NIR vs OSCC-CM NIR	0.0321
	No FBS NIR vs OSCC-CM/GW NIR	0.0049
	OSCC-CM NIR vs OSCC-CM/GW NIR	0.9044
	No FBS IR vs TGFβ IR	0.0026
	No FBS IR vs OSCC-CM IR	0.893
	No FBS IR vs OSCC-CM/GW IR	0.9759
	OSCC-CM IR vs OSCC-CM/GW IR	0.677
	No FBS NIR vs IR	0.0004
	TGFβ NIR vs IR	0.0992
	OSCC-CM NIR vs IR	0.1569
	OSCC-CM/GW NIR vs IR	0.9977
S4B	shC vs shT	0.3265
	shC vs KO	0.426
	shT vs KO	0.0472
S4D	WT vs KO	0.023
S4E	GP38 FRC fraction vs OSCC fraction	0.0019
	ITGA7 FRC fraction vs OSCC fraction	<0.0001
	Cdh1 FRC fraction vs OSCC fraction	<0.0001
S4G	shC NIR vs shT NIR	0.5201
	shC NIR vs KO NIR	0.7117
	shC NIR vs shC IR	0.5254
	shC NIR vs shT IR	0.9989
	shC NIR vs KO IR	0.4217
S4H	shC NIR vs shT NIR	0.8761
	shC NIR vs KO NIR	0.9872
	shC NIR vs shC IR	0.9998
	shC NIR vs shT IR	0.999
	shC NIR vs KO IR	0.9805
S4I	shC NIR vs shT NIR	0.9036
	shC NIR vs KO NIR	0.44

	shC NIR vs shC IR	<0.0001
	shC NIR vs shT IR	0.9994
	shC NIR vs KO IR	0.3586
S4J	shC NIR vs shT NIR	>0.9999
	shC NIR vs KO NIR	>0.9999
	shC NIR vs shC IR	0.0001
	shC NIR vs shT IR	0.1102
	shC NIR vs KO IR	0.8838
S4K	shC NIR vs shT NIR	>0.9999
	shC NIR vs KO NIR	0.9998
	shC NIR vs shC IR	0.032
	shC NIR vs shT IR	>0.9999
	shC NIR vs KO IR	>0.9999
S4L	shC NIR vs shT NIR	0.9815
	shC NIR vs KO NIR	0.9897
	shC NIR vs shC IR	0.0002
	shC NIR vs shT IR	>0.9999
	shC NIR vs KO IR	>0.9999
S4M	shC NIR vs shT NIR	0.9979
	shC NIR vs KO NIR	0.9998
	shC NIR vs shC IR	<0.0001
	shC NIR vs shT IR	>0.9999
	shC NIR vs KO IR	>0.9999
S4N	shC NIR vs shT NIR	0.9998
	shC NIR vs KO NIR	>0.9999
	shC NIR vs shC IR	<0.0001
	shC NIR vs shT IR	0.9703
	shC NIR vs KO IR	0.9995
S4O	shC NIR vs shT NIR	>0.9999
	shC NIR vs KO NIR	>0.9999
	shC NIR vs shC IR	<0.0001
	shC NIR vs shT IR	0.2368
	shC NIR vs KO IR	0.1799
S4T (cdh1 oscc mono)	NIR vs IR	0.9997
S4Q (Tgfb1 Oscc mono)	NIR vsIR	0.0096
S4R	NIR vs IR	0.022
S4S	NIR vs IR	0.008
S4P	NIR vs IR	0.0032
S4V	shC NIR vs shT NIR	0.6117

	shC NIR vs KO NIR	0.3908
	shC NIR vs shC IR	>0.9999
	shC NIR vs shT IR	0.0703
	shC NIR vs KO IR	0.2481
S4U	shC NIR vs shT NIR	>0.9999
	shC NIR vs KO NIR	>0.9999
	shC NIR vs shC IR	0.0181
	shC NIR vs shT IR	0.8314
	shC NIR vs KO IR	0.9309
S4W	shC NIR vs shT NIR	0.7472
	shC NIR vs KO NIR	0.9522
	shC NIR vs shC IR	>0.9999
	shC NIR vs shT IR	0.0015
	shC NIR vs KO IR	0.0032
S6C	NIR vs 5x2Gy	0.1216
S6E	NIR vs 5x2Gy	0.0253
S6F	NIR vs NIR + MP5	0.9995
	NIR vs IR	0.8065
	NIR vs IR + MP5	0.6839
	IR vs IR+ MP5	0.9958
