# Supplementary figures and images for: Tenascin-C orchestrates radiotherapy-induced head and neck tumor regression
Source: EMBO Mol Med. 2026 Mar 31;18(5):1707–43. doi: 10.1038/s44321-026-00406-8 (PMC13179387; doi:10.1038/s44321-026-00406-8)

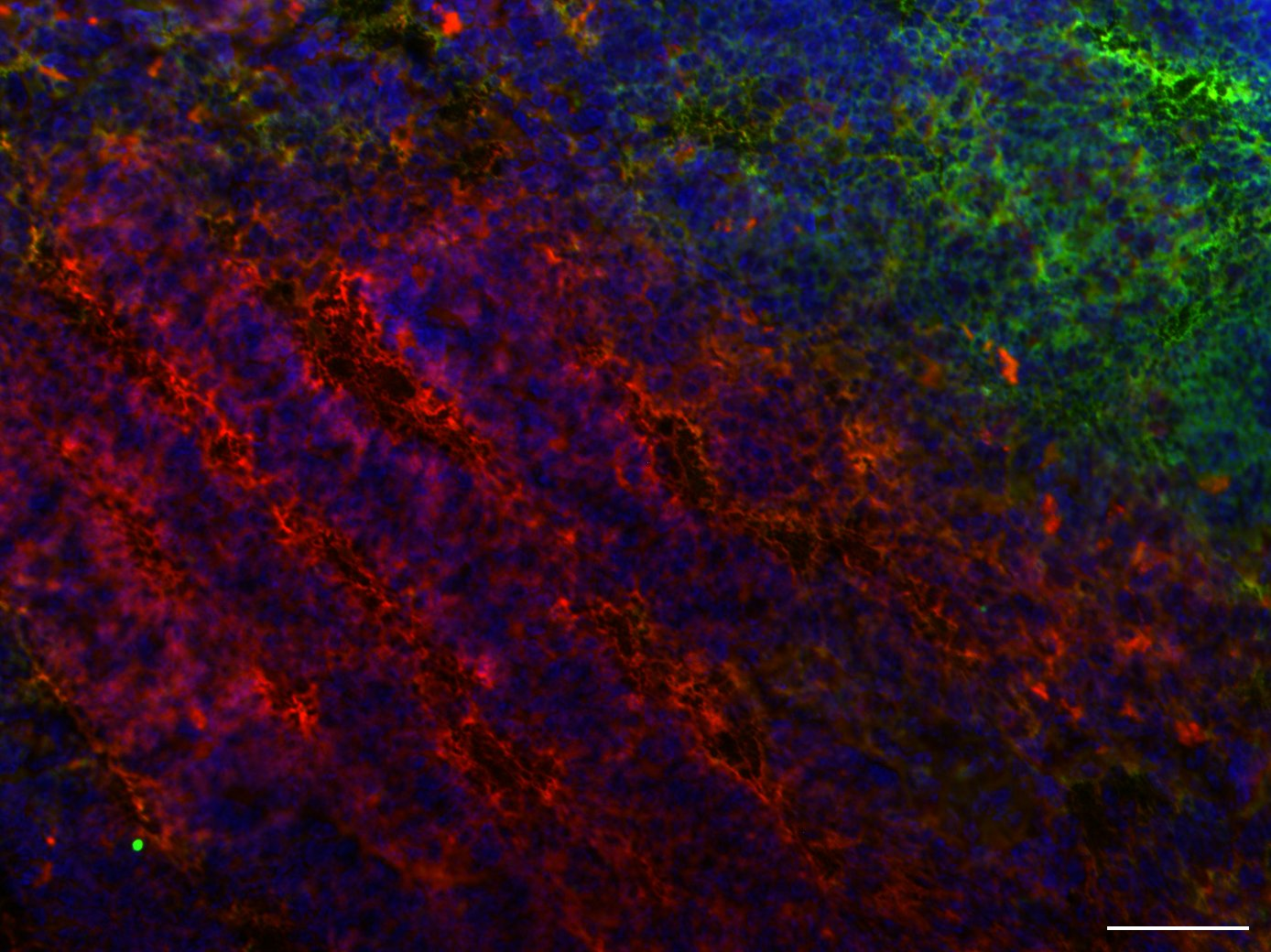

Supplement: Supplementary file 3 — Source data Fig. 1 [file 44321_2026_406_MOESM3_ESM.zip › Figure 1/Fig1J/LN WT CD3 B220.tif]

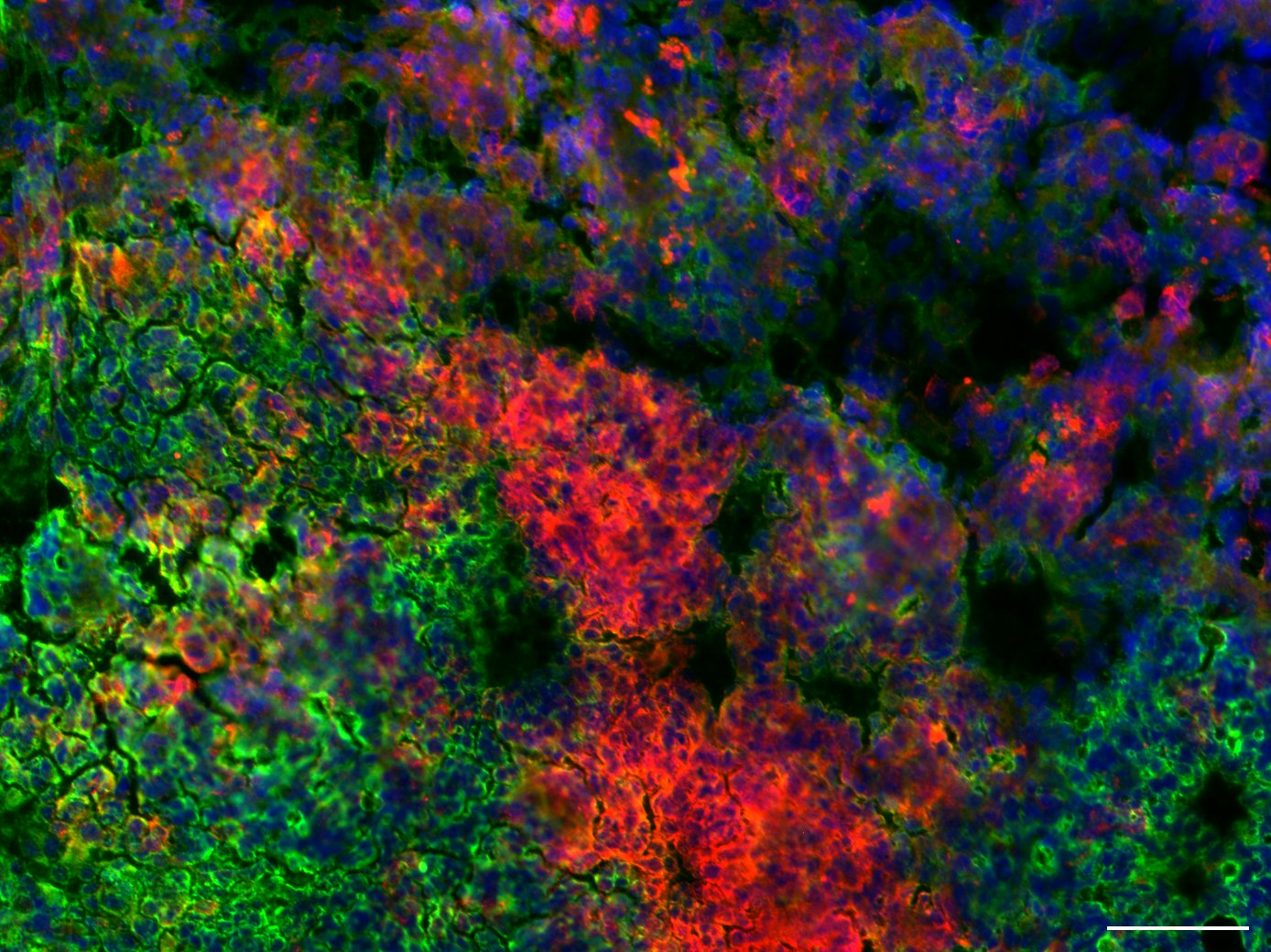

Supplement: Supplementary file 3 — Source data Fig. 1 [file 44321_2026_406_MOESM3_ESM.zip › Figure 1/Fig1J/LNKO CD3 B220.tif]

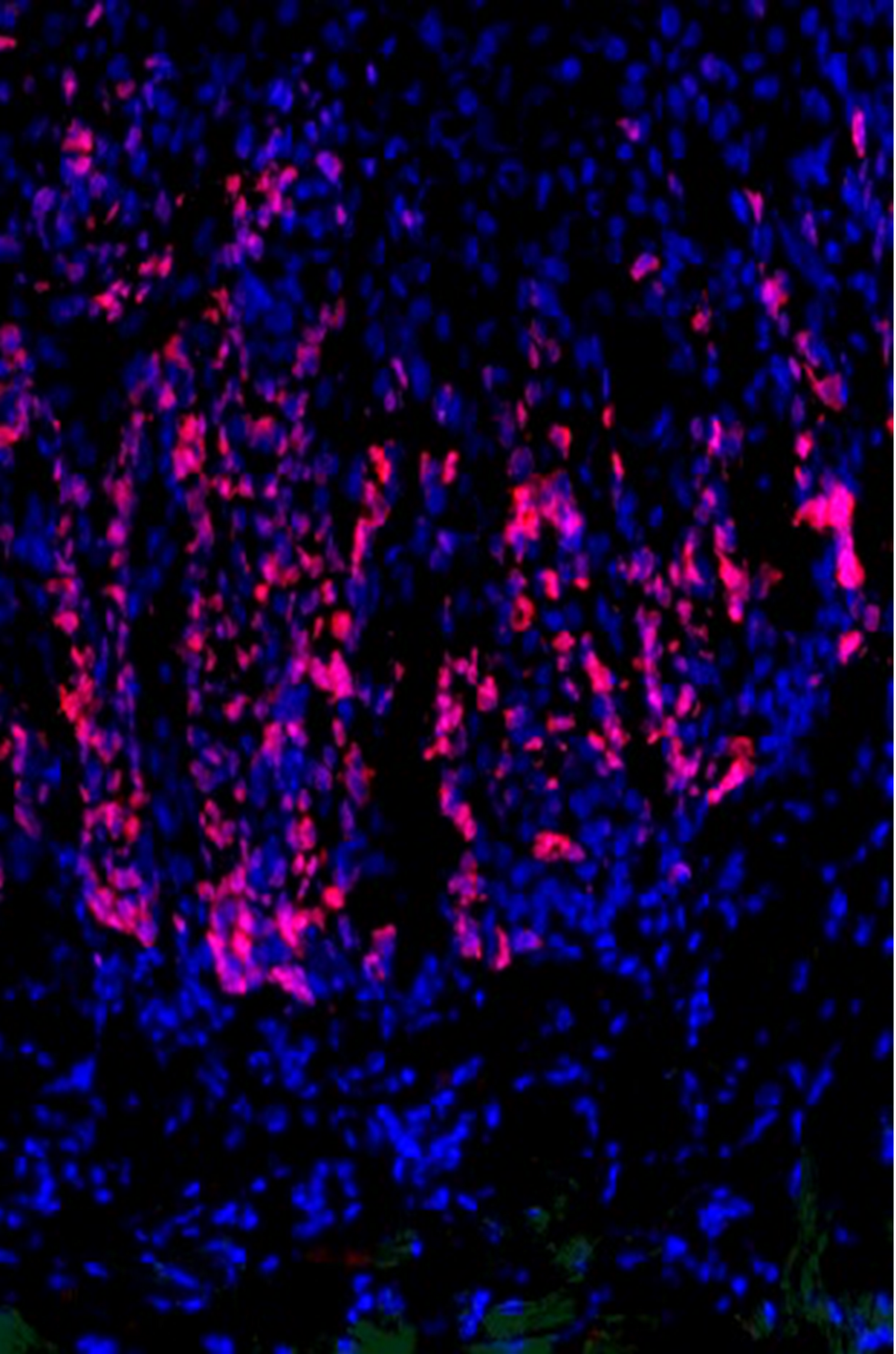

Supplement: Supplementary file 3 — Source data Fig. 1 [file 44321_2026_406_MOESM3_ESM.zip › Figure 1/fig 1B/3- Fig 1B KO_NIR.TIF]

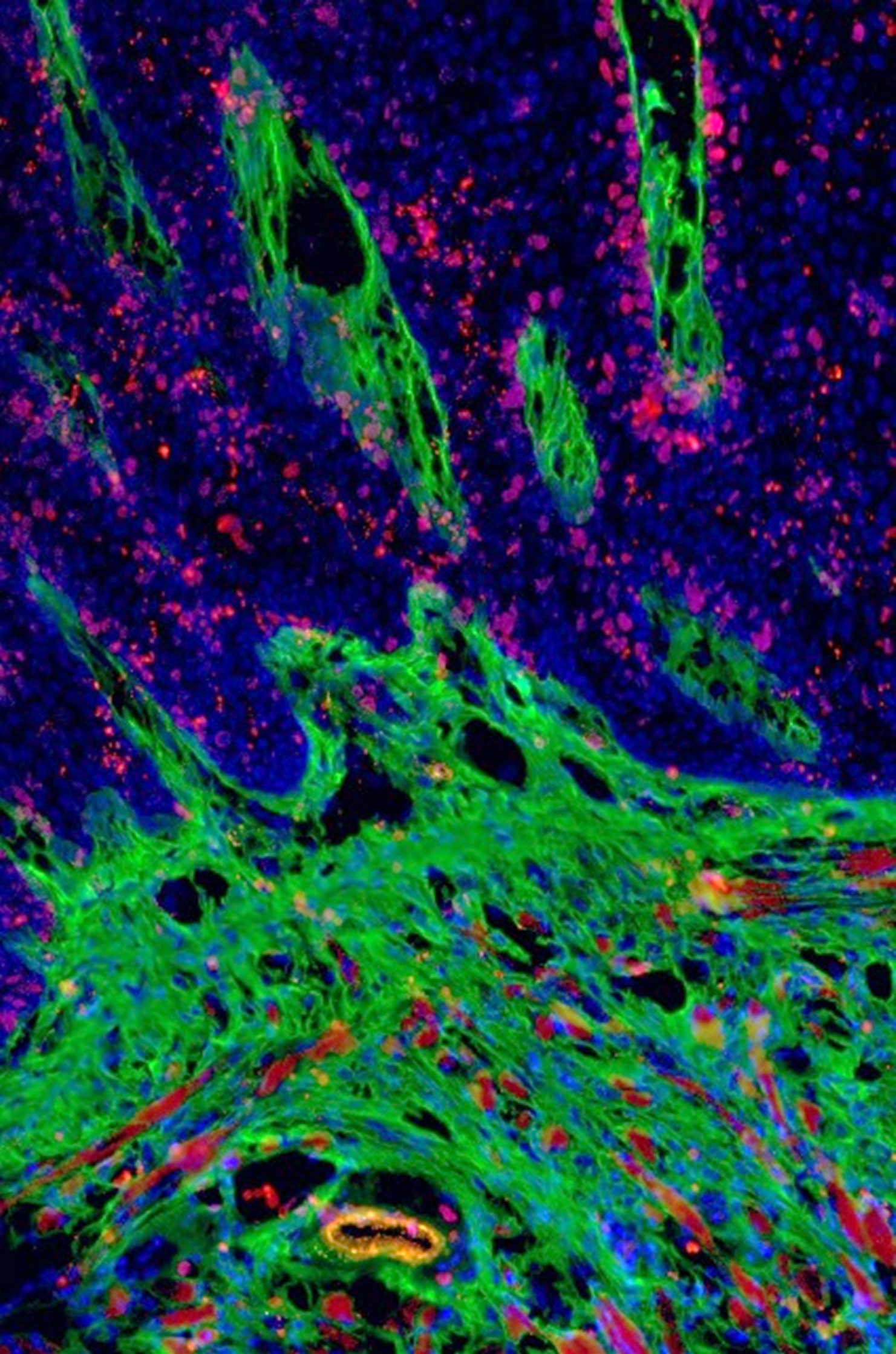

Supplement: Supplementary file 3 — Source data Fig. 1 [file 44321_2026_406_MOESM3_ESM.zip › Figure 1/fig 1B/2- Fig 1B WT_IR.TIF]

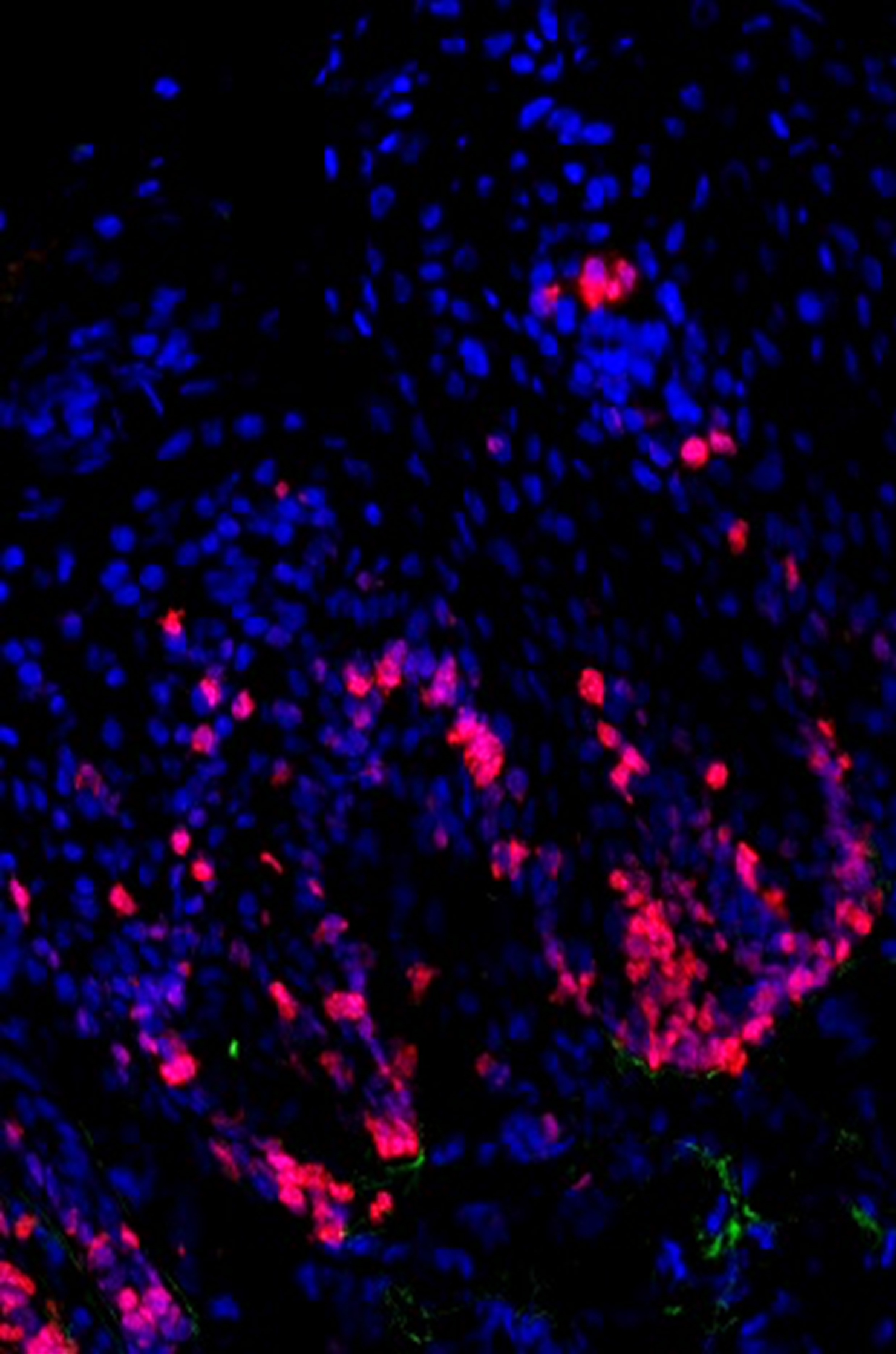

Supplement: Supplementary file 3 — Source data Fig. 1 [file 44321_2026_406_MOESM3_ESM.zip › Figure 1/fig 1B/4- Fig 1B KO_IR.TIF]

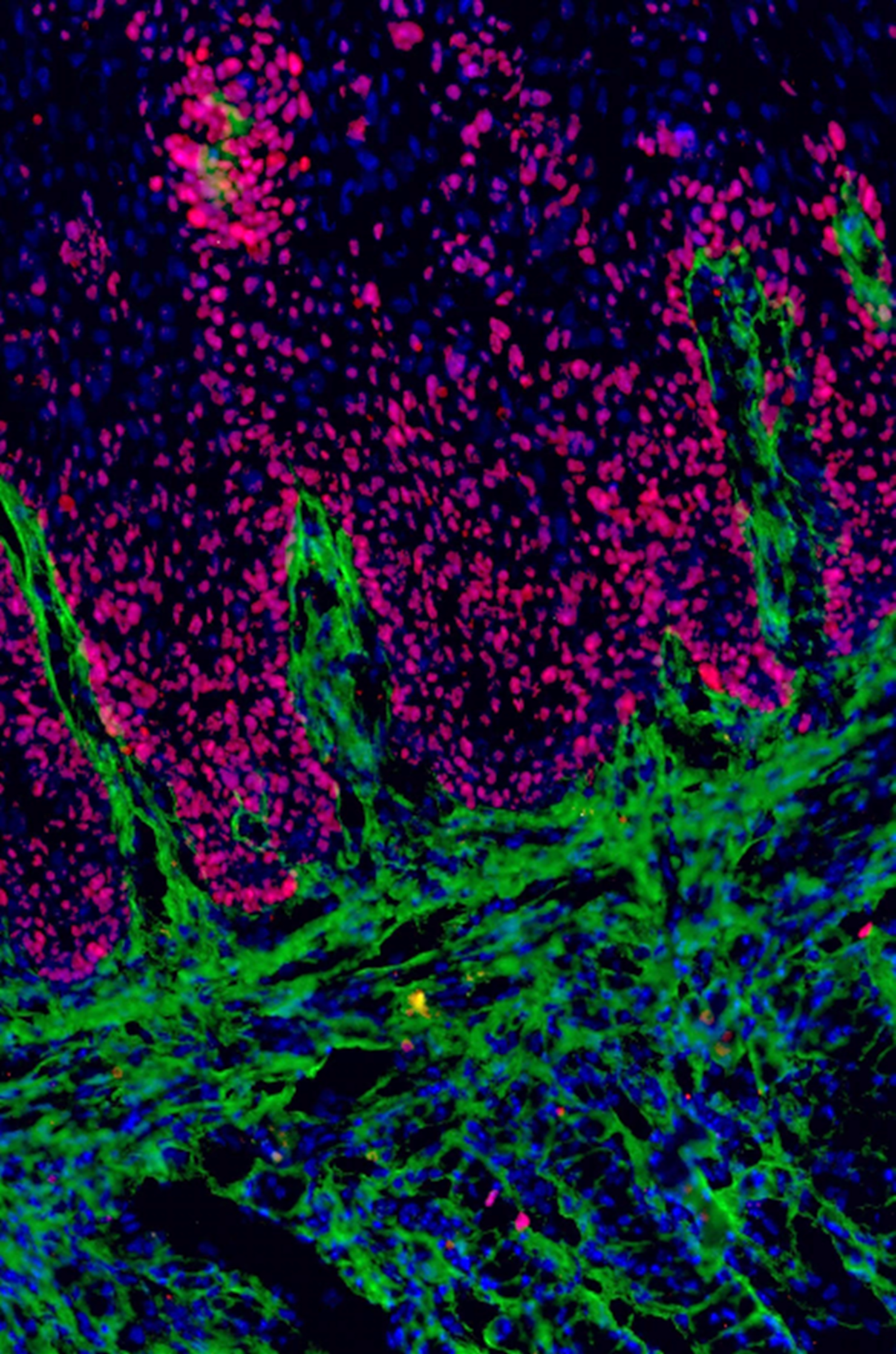

Supplement: Supplementary file 3 — Source data Fig. 1 [file 44321_2026_406_MOESM3_ESM.zip › Figure 1/fig 1B/1- Fig 1B WT_NIR.TIF]

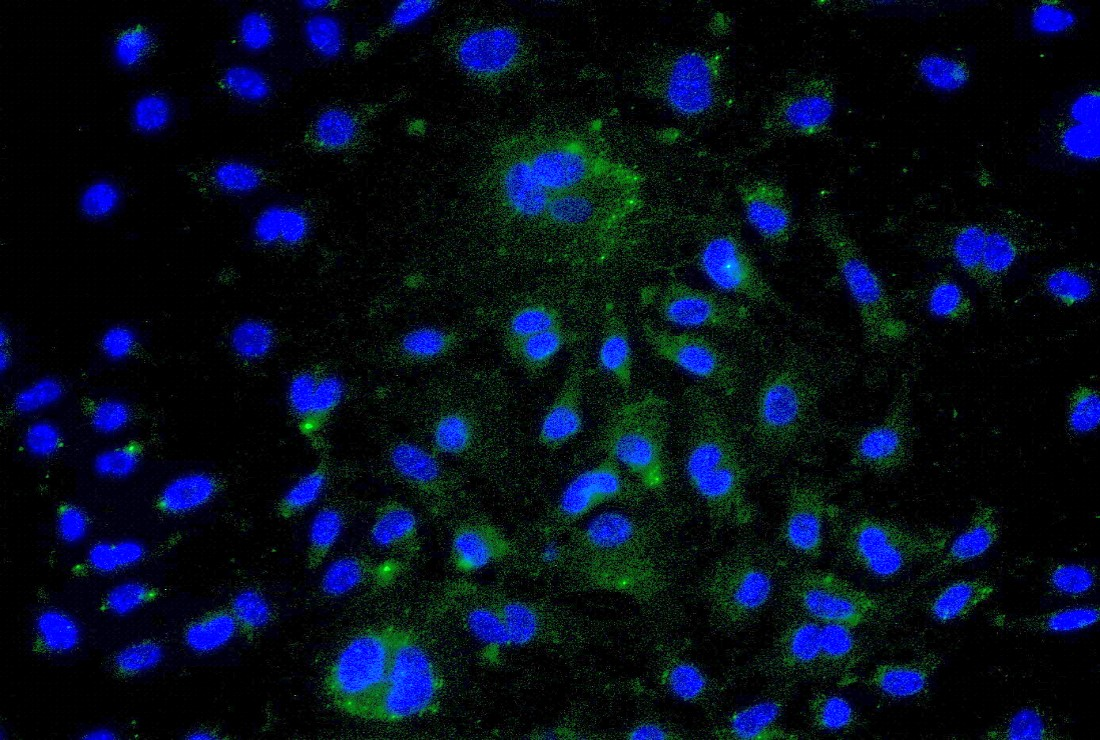

Supplement: Supplementary file 4 — Source data Fig. 2 [file 44321_2026_406_MOESM4_ESM.zip › Figure 2/2A/CCL21 TNC KO.tif]

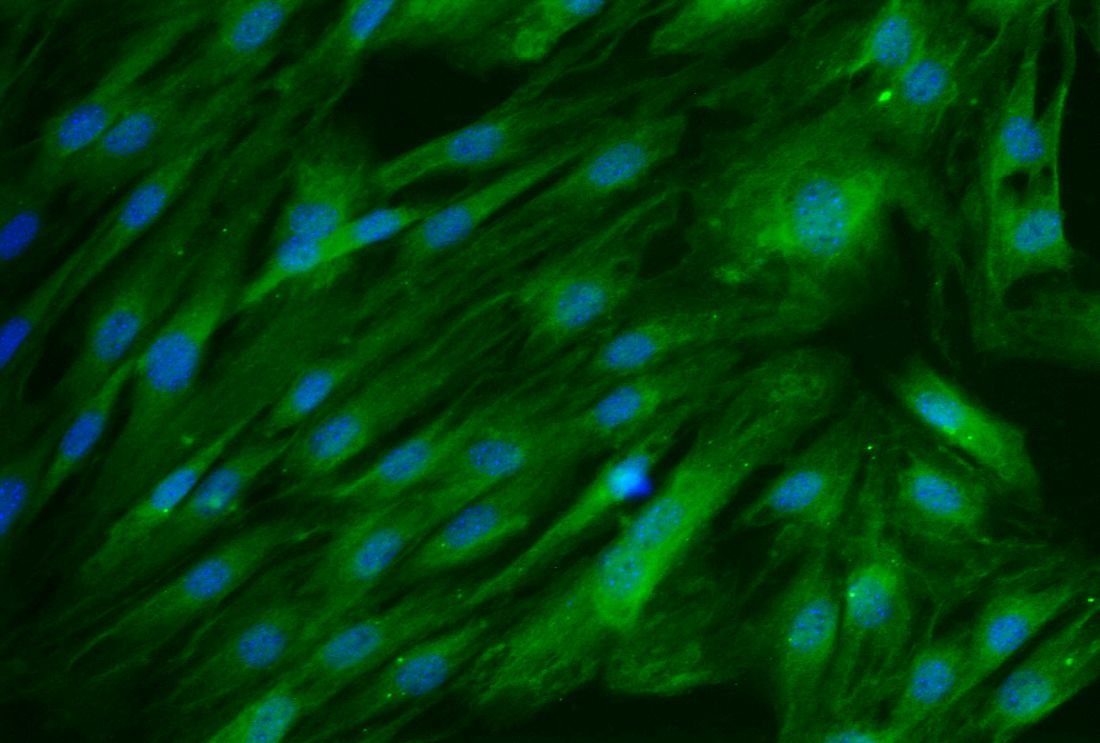

Supplement: Supplementary file 4 — Source data Fig. 2 [file 44321_2026_406_MOESM4_ESM.zip › Figure 2/2A/CCL21 TNC WT.tif]

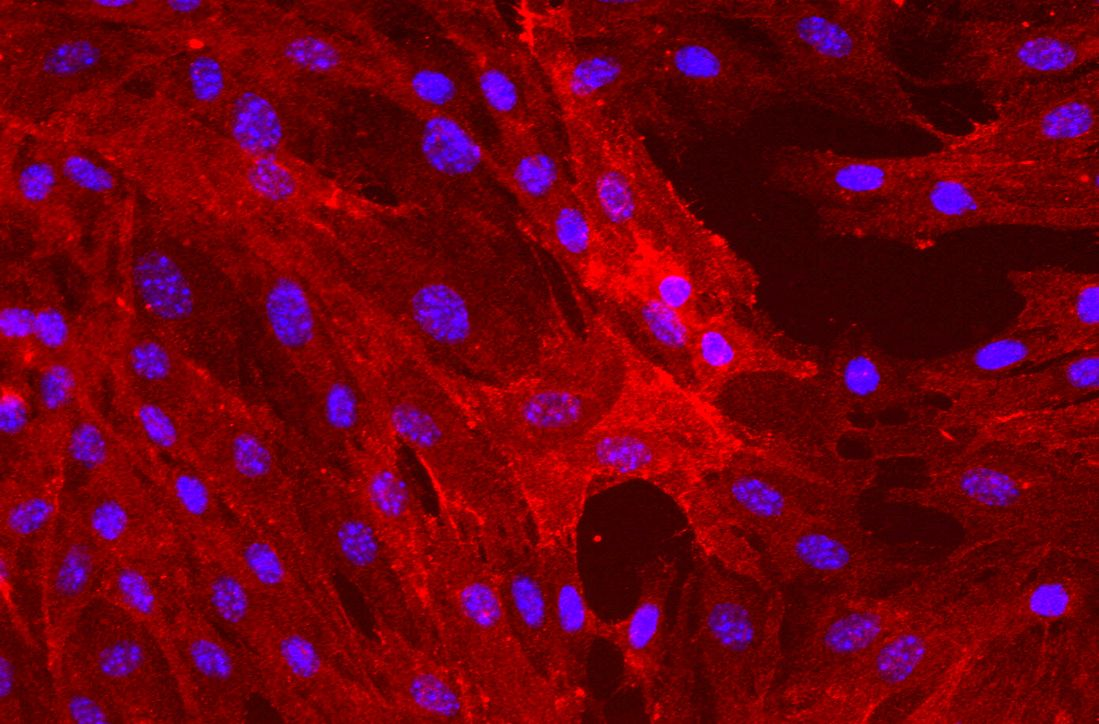

Supplement: Supplementary file 4 — Source data Fig. 2 [file 44321_2026_406_MOESM4_ESM.zip › Figure 2/2A/gp38 TNC WT.tif]

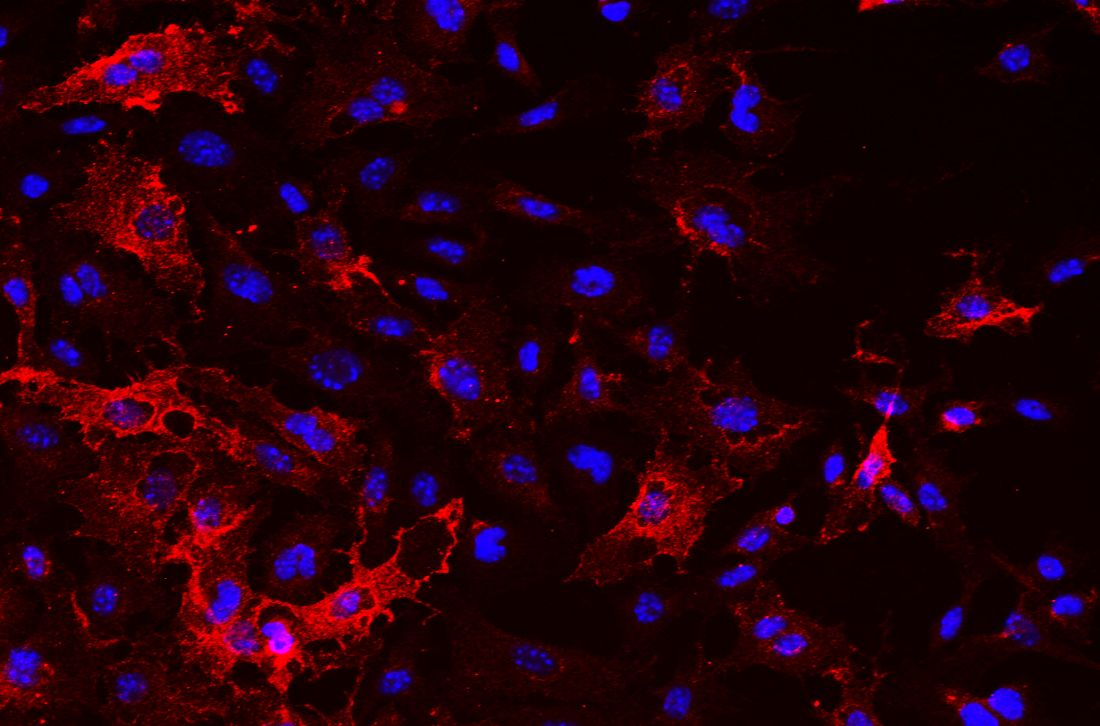

Supplement: Supplementary file 4 — Source data Fig. 2 [file 44321_2026_406_MOESM4_ESM.zip › Figure 2/2A/gp38 TNC KO.tif]

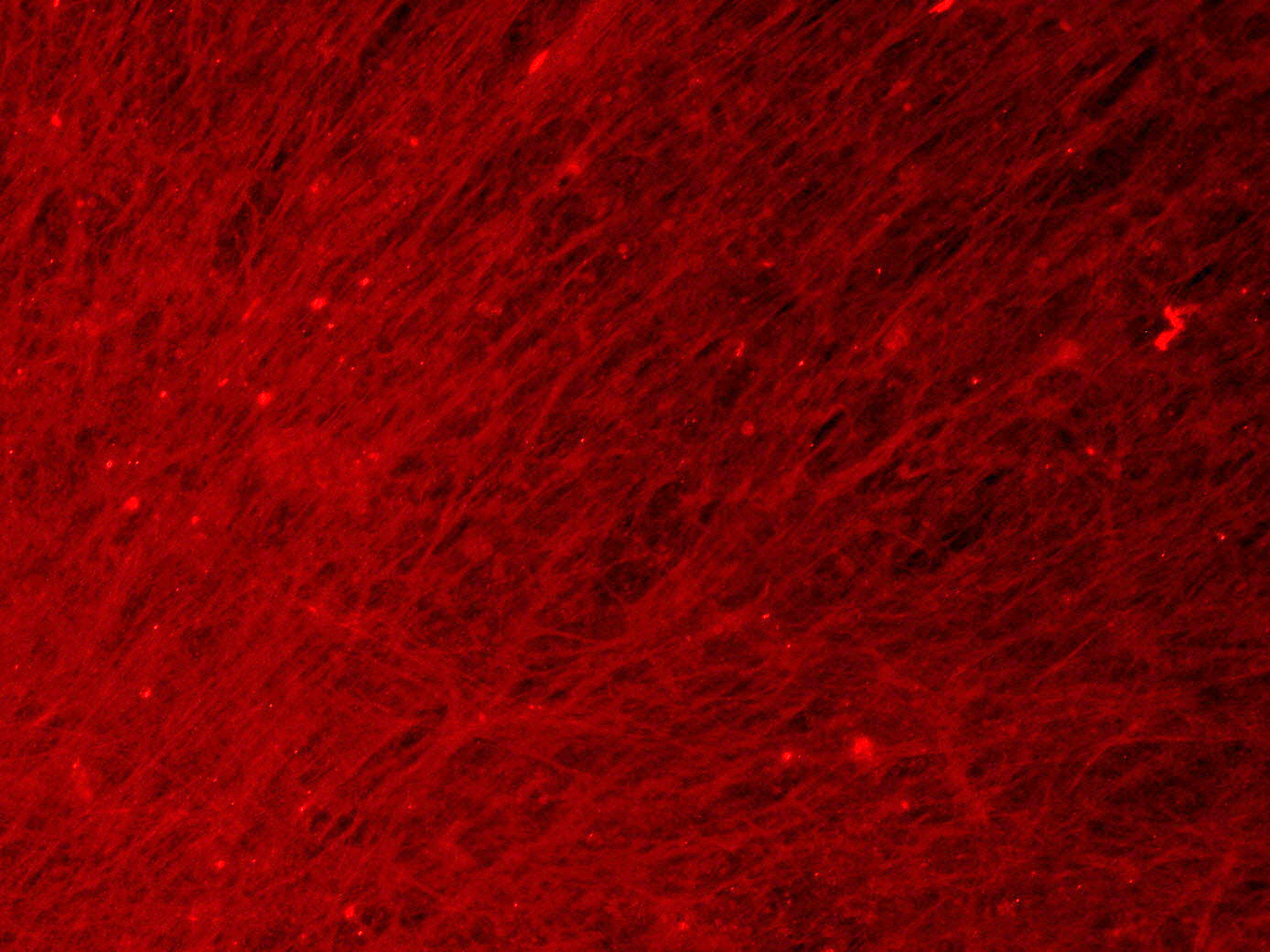

Supplement: Supplementary file 4 — Source data Fig. 2 [file 44321_2026_406_MOESM4_ESM.zip › Figure 2/fig 2E/CDM-FRCWT-FN.TIF]

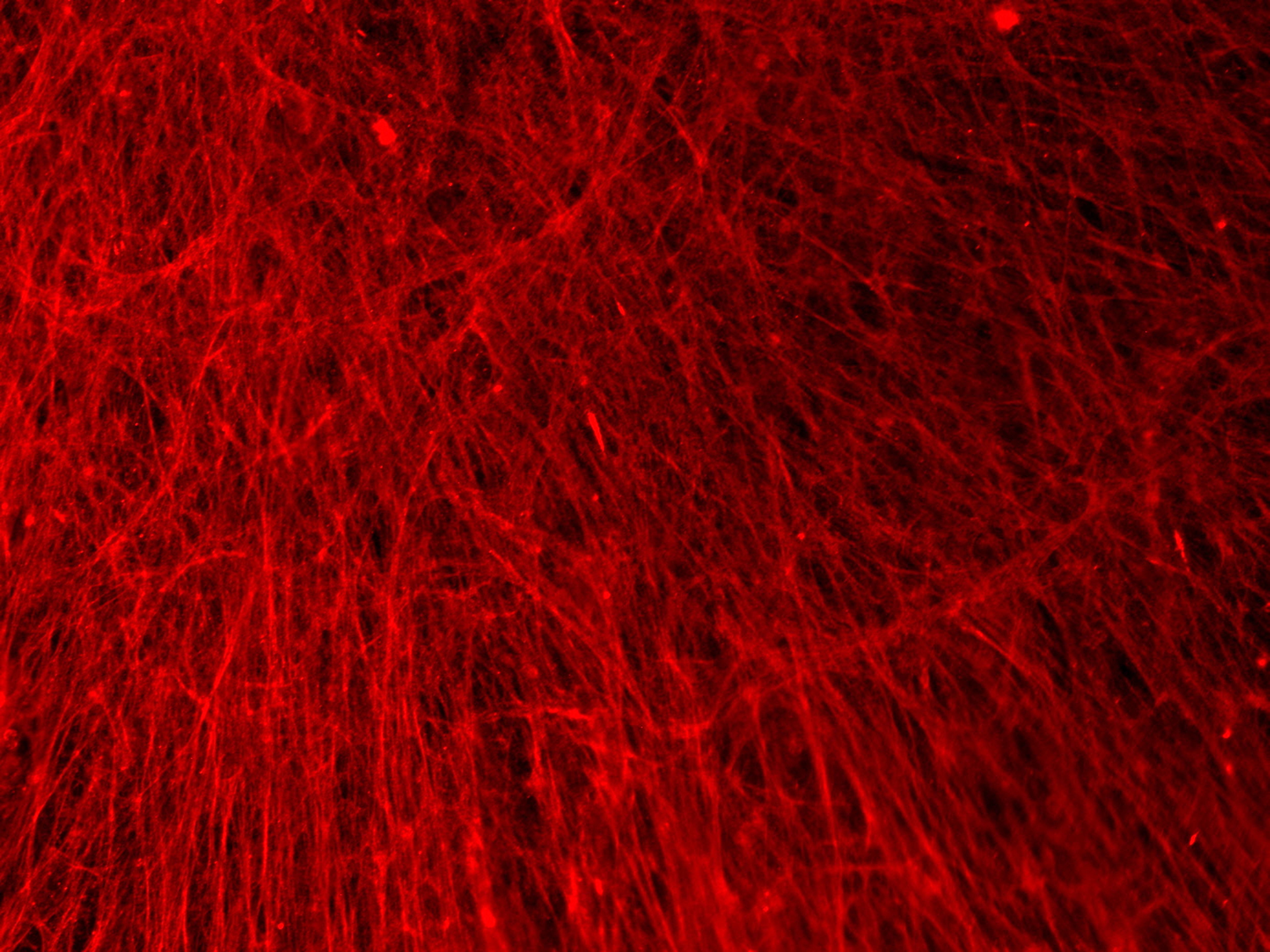

Supplement: Supplementary file 4 — Source data Fig. 2 [file 44321_2026_406_MOESM4_ESM.zip › Figure 2/fig 2E/CDM-FRCWT-ColIV.TIF]

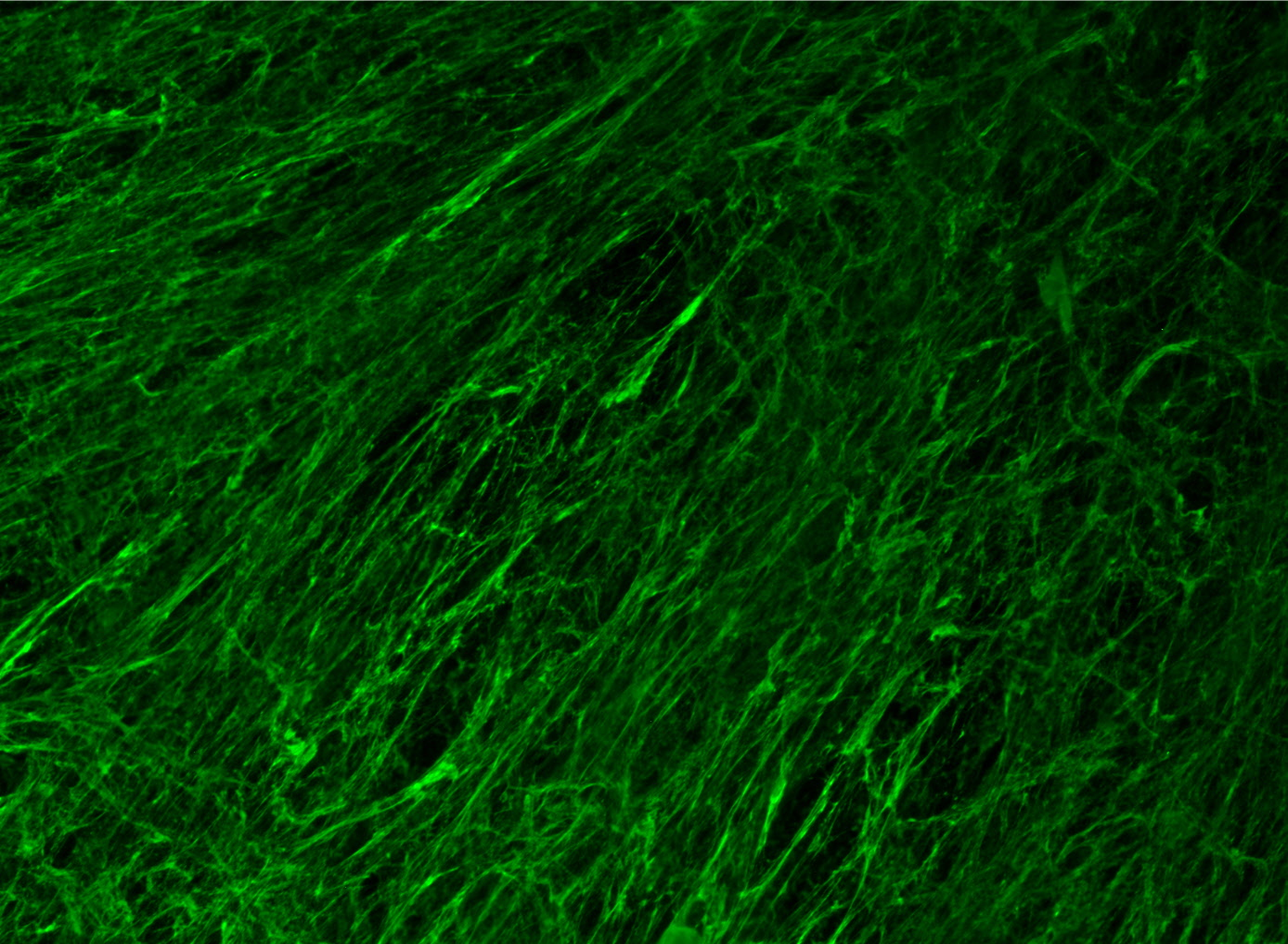

Supplement: Supplementary file 4 — Source data Fig. 2 [file 44321_2026_406_MOESM4_ESM.zip › Figure 2/fig 2E/2- Fig 2E-KO_TNC+ColXII (2).TIF]

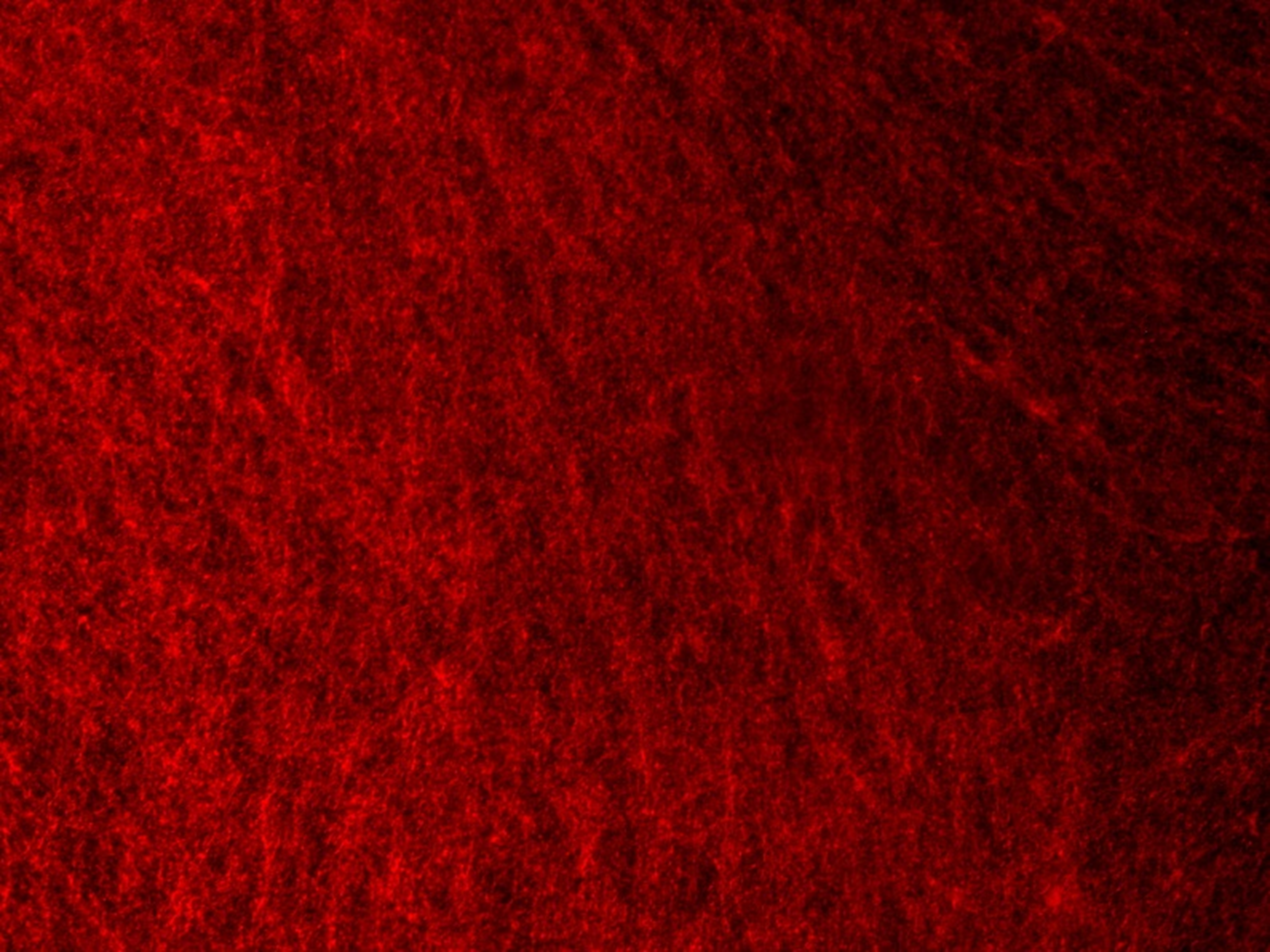

Supplement: Supplementary file 4 — Source data Fig. 2 [file 44321_2026_406_MOESM4_ESM.zip › Figure 2/fig 2E/CDM-FRCKO-FN.TIF]

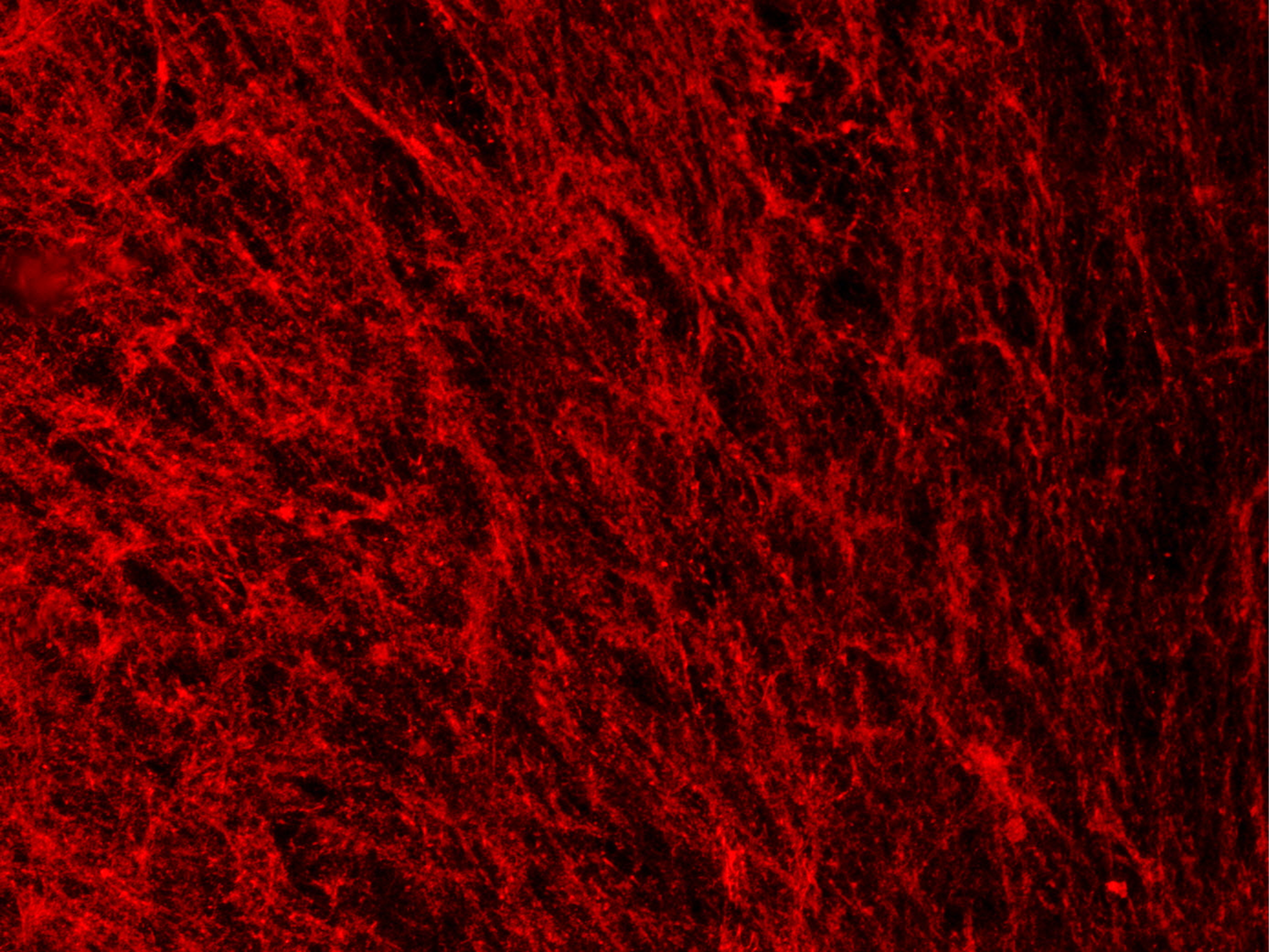

Supplement: Supplementary file 4 — Source data Fig. 2 [file 44321_2026_406_MOESM4_ESM.zip › Figure 2/fig 2E/CDM-FRCKO-ColIV.TIF]

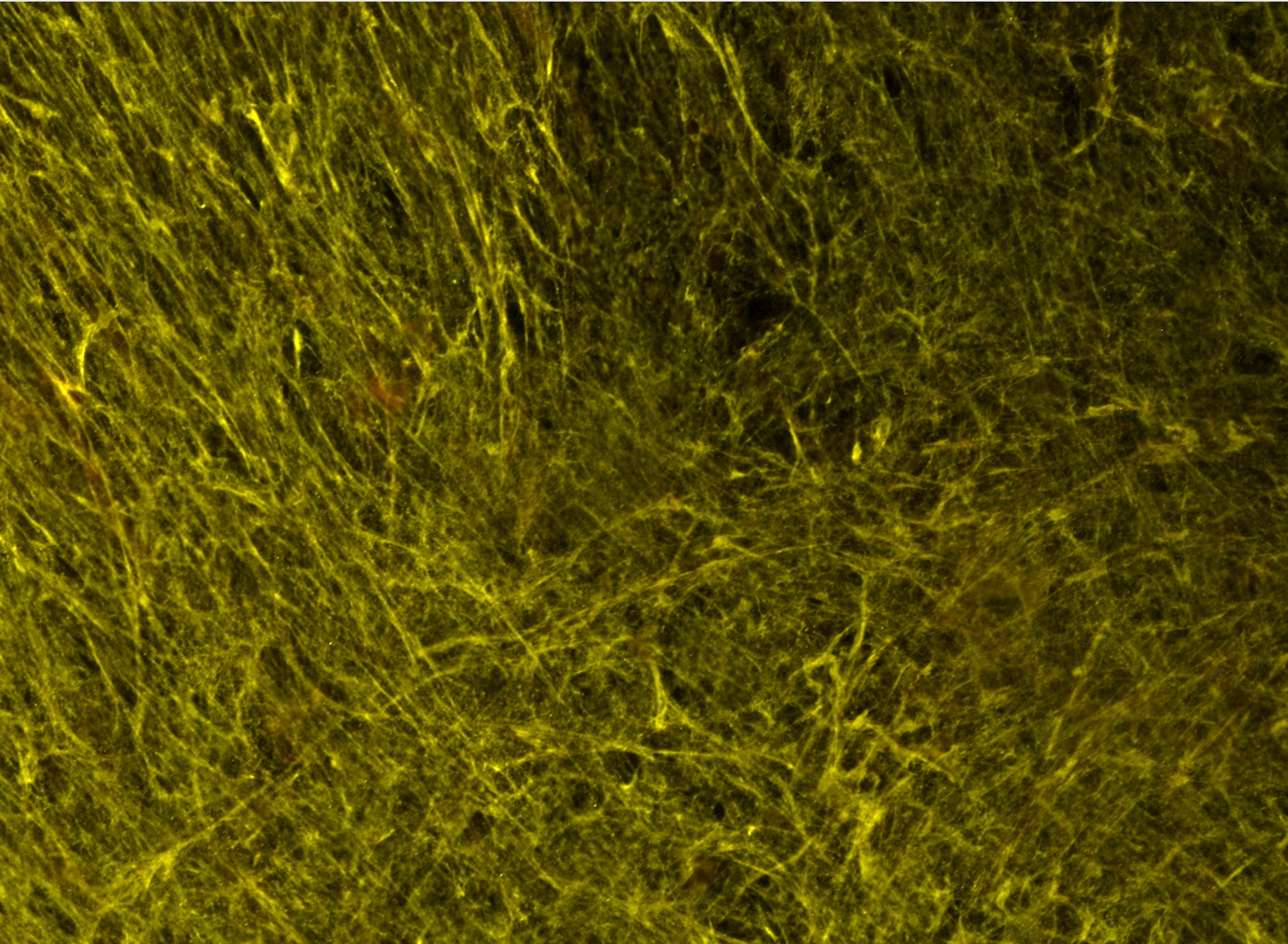

Supplement: Supplementary file 4 — Source data Fig. 2 [file 44321_2026_406_MOESM4_ESM.zip › Figure 2/fig 2E/1- Fig 2E-WT_TNC+ColXII (2).TIF]

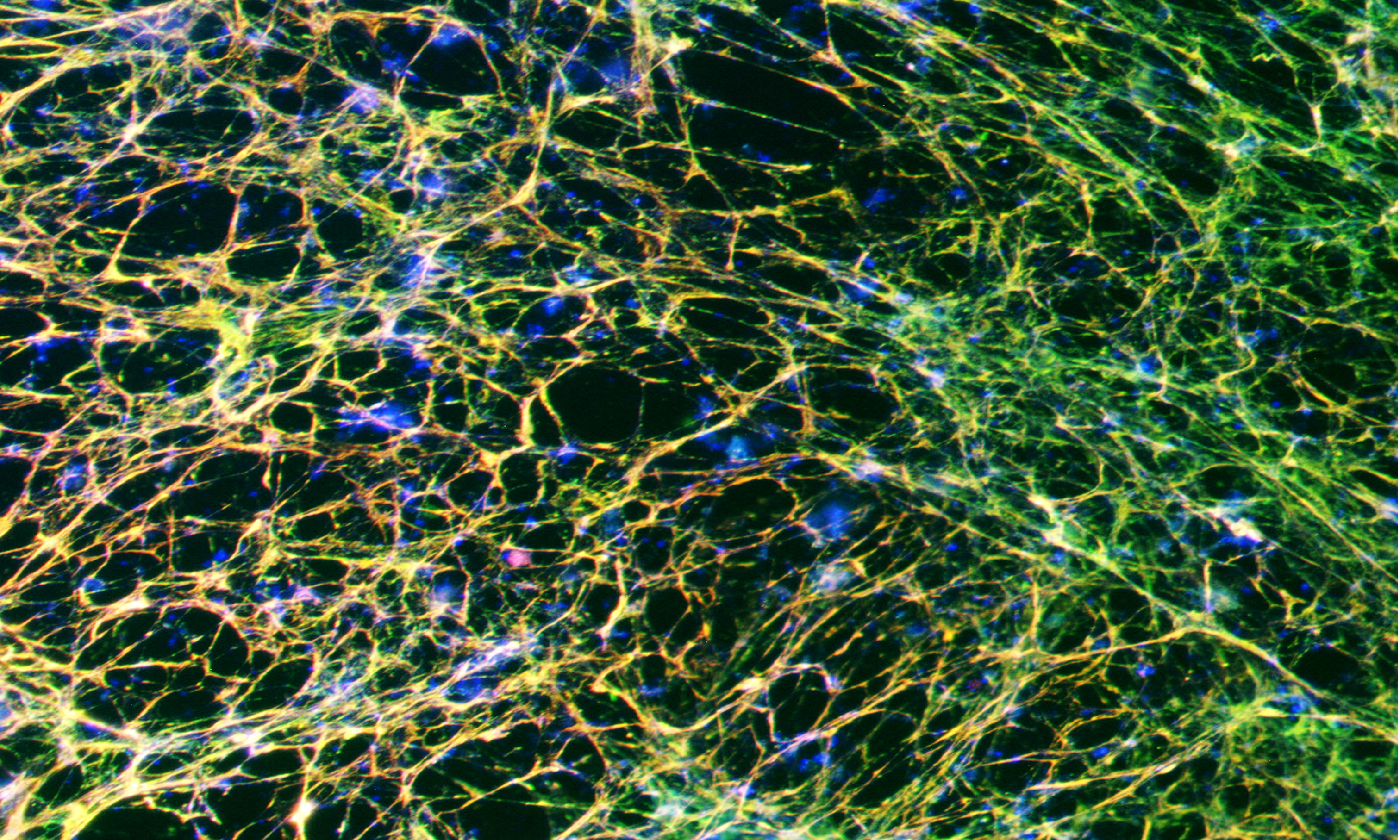

Supplement: Supplementary file 5 — Source data Fig. 3 [file 44321_2026_406_MOESM5_ESM.zip › Figure 3/fig 3E/2- Fig 3E_WT IR.TIF]

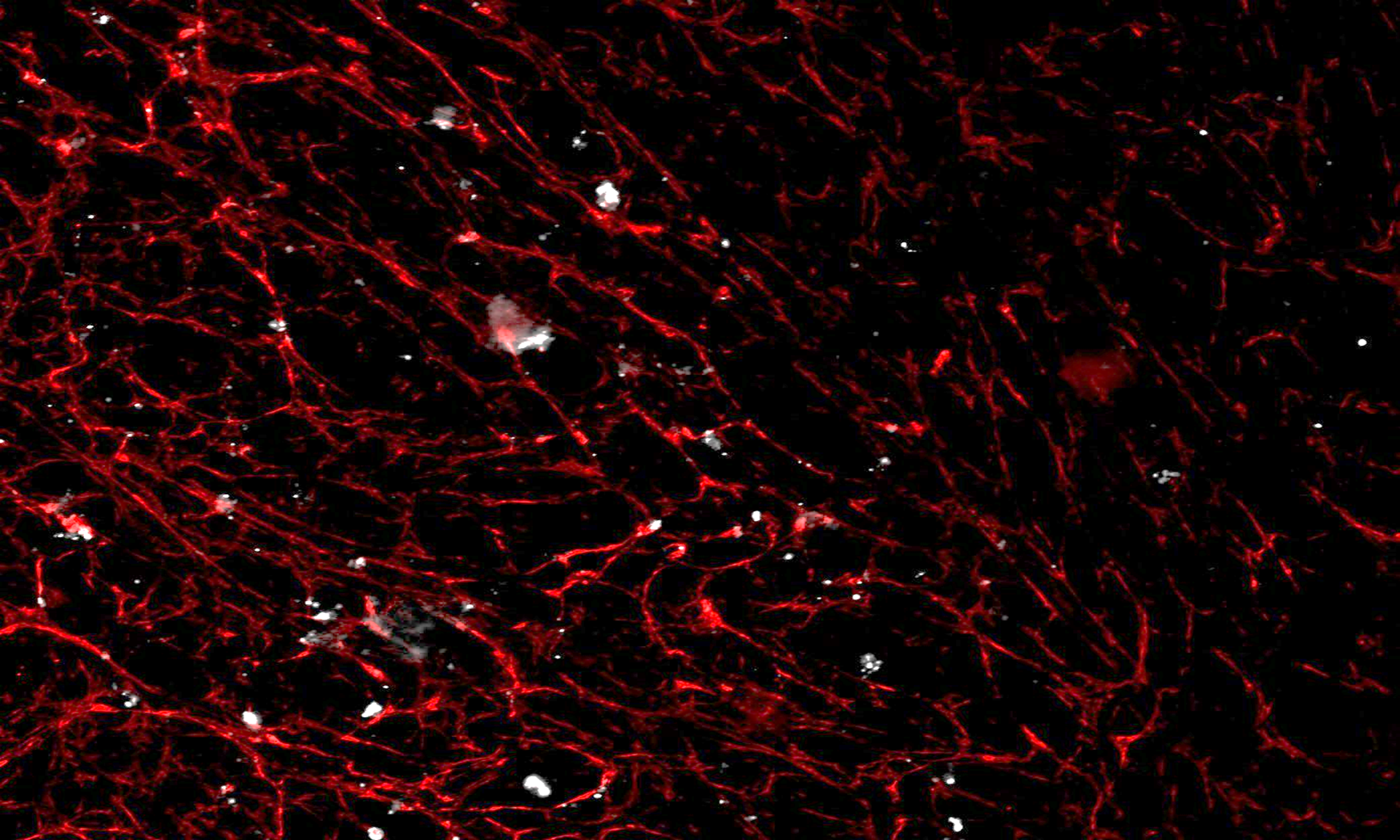

Supplement: Supplementary file 5 — Source data Fig. 3 [file 44321_2026_406_MOESM5_ESM.zip › Figure 3/fig 3E/3- Fig 3E_KO NIR.TIF]

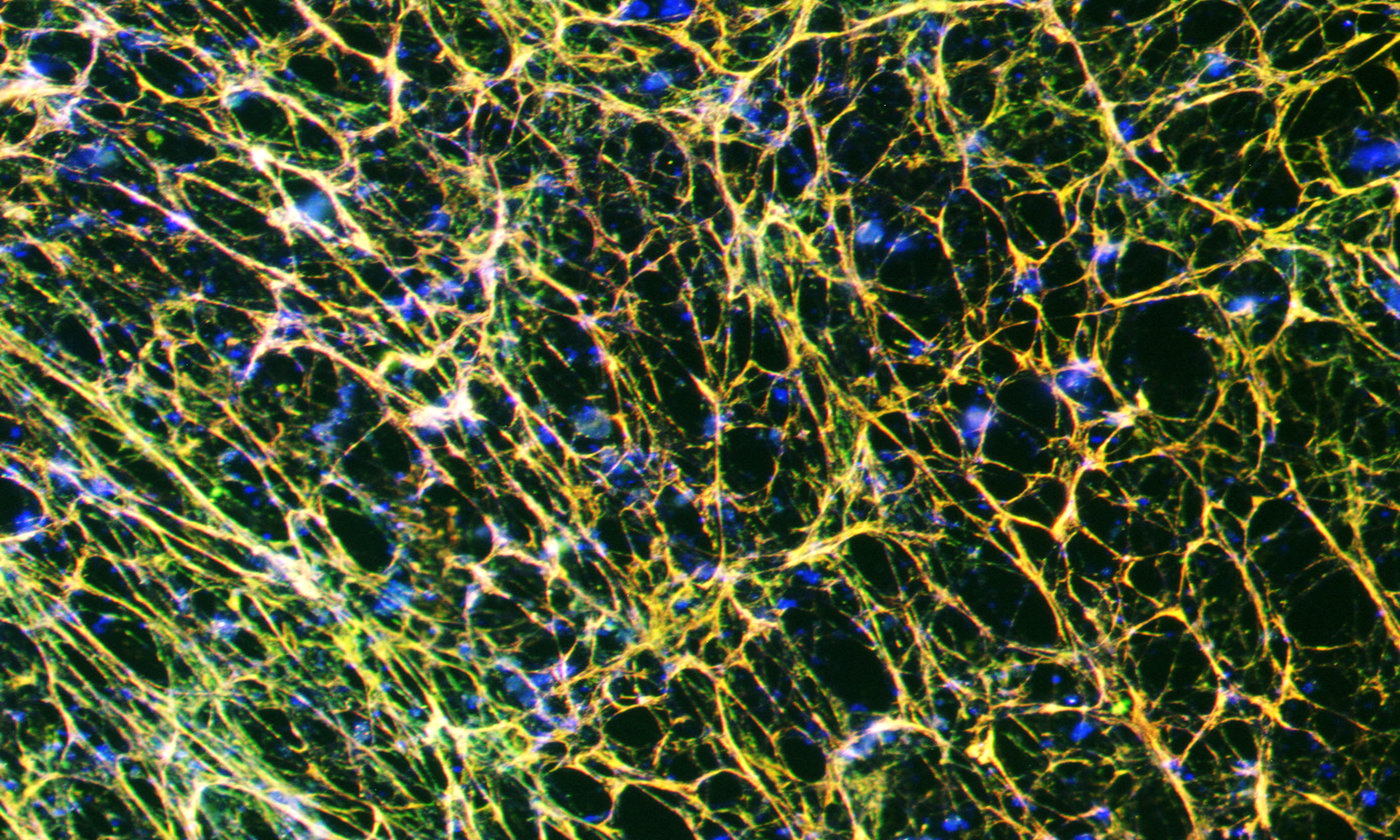

Supplement: Supplementary file 5 — Source data Fig. 3 [file 44321_2026_406_MOESM5_ESM.zip › Figure 3/fig 3E/1- Fig 3E_WT-NIR.TIF]

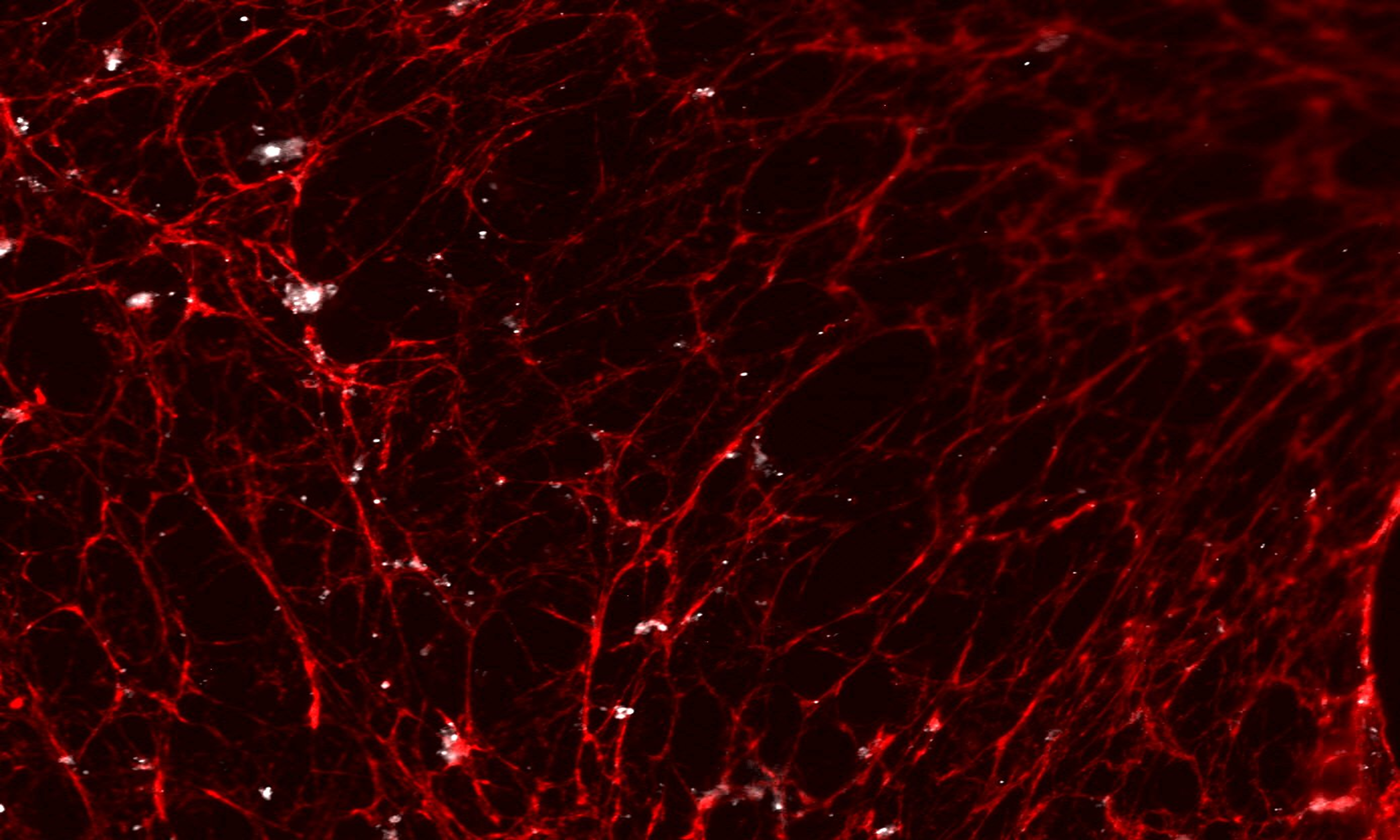

Supplement: Supplementary file 5 — Source data Fig. 3 [file 44321_2026_406_MOESM5_ESM.zip › Figure 3/fig 3E/4- Fig 3E_KO IR.TIF]

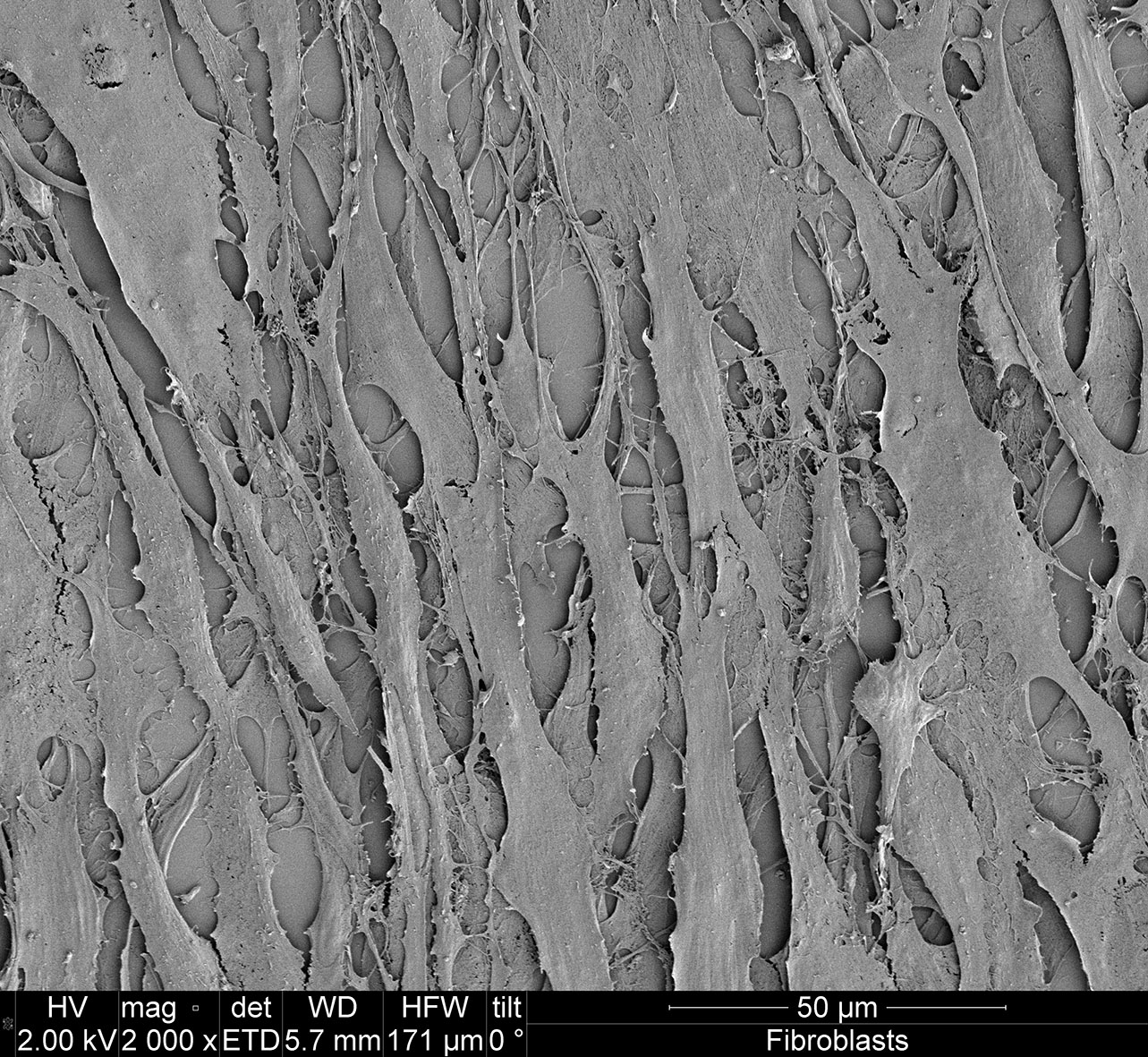

Supplement: Supplementary file 5 — Source data Fig. 3 [file 44321_2026_406_MOESM5_ESM.zip › Figure 3/3A/WT NIR.tif]

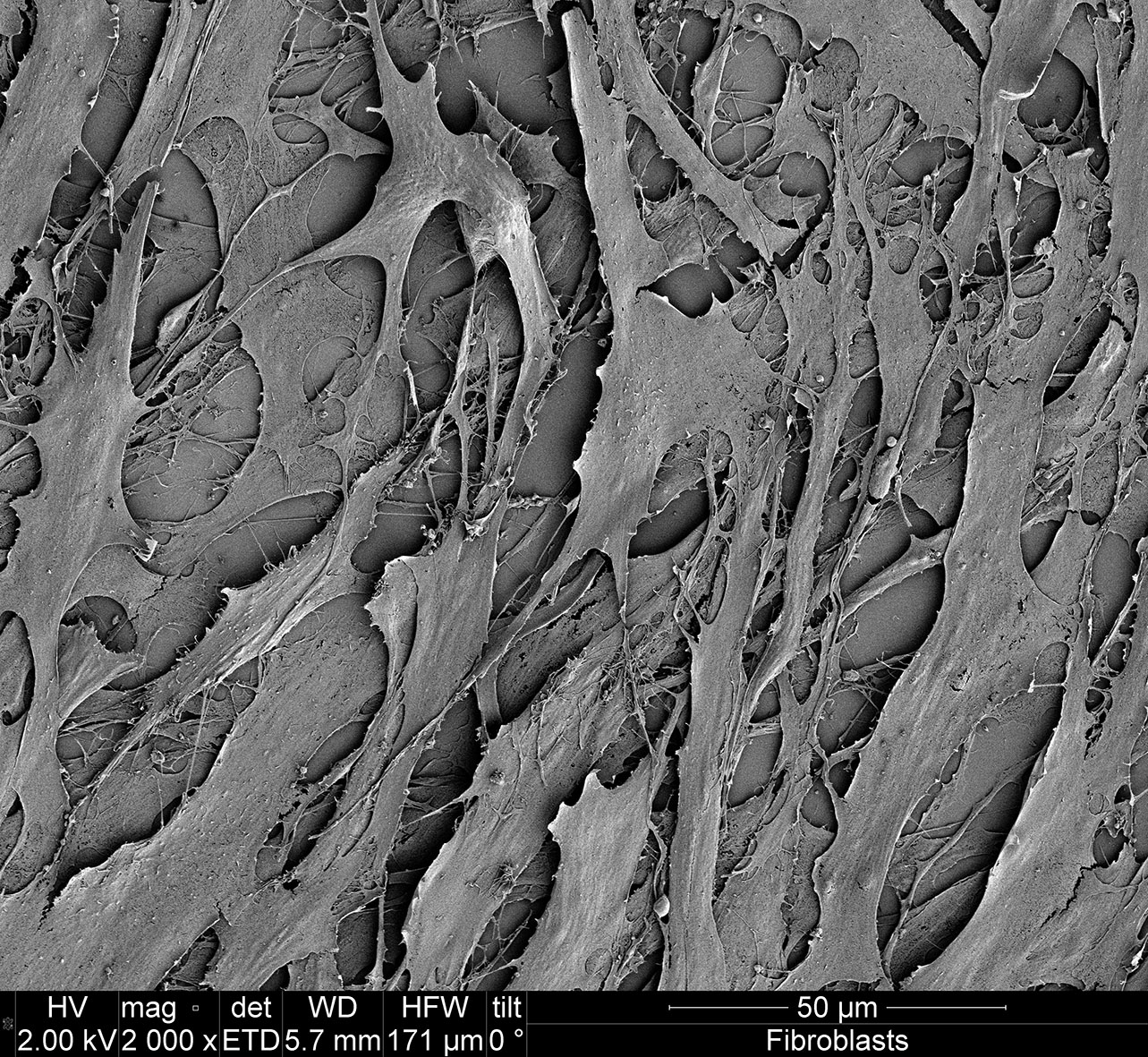

Supplement: Supplementary file 5 — Source data Fig. 3 [file 44321_2026_406_MOESM5_ESM.zip › Figure 3/3A/KO NIR.tif]

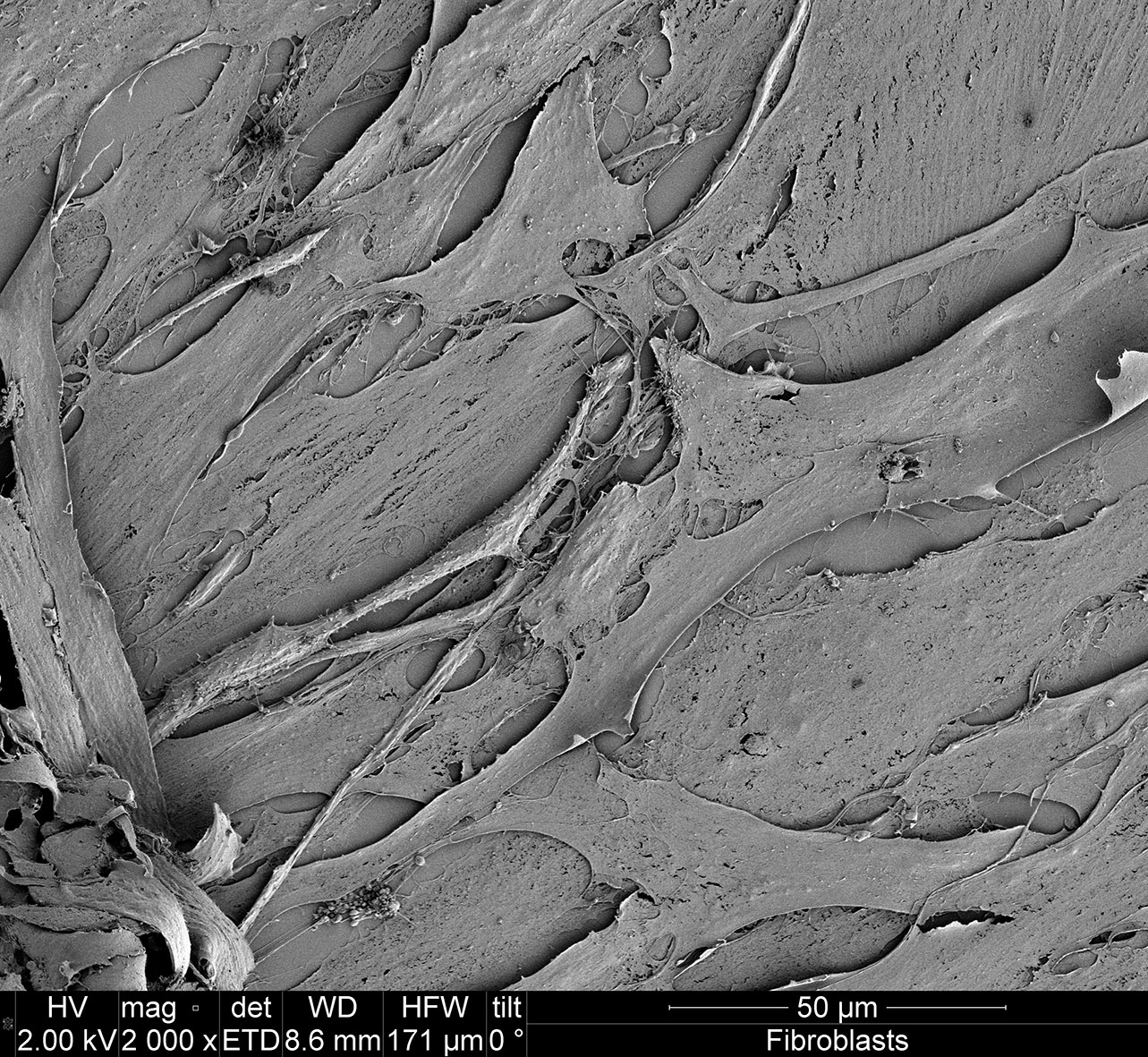

Supplement: Supplementary file 5 — Source data Fig. 3 [file 44321_2026_406_MOESM5_ESM.zip › Figure 3/3A/KO IR.tif]

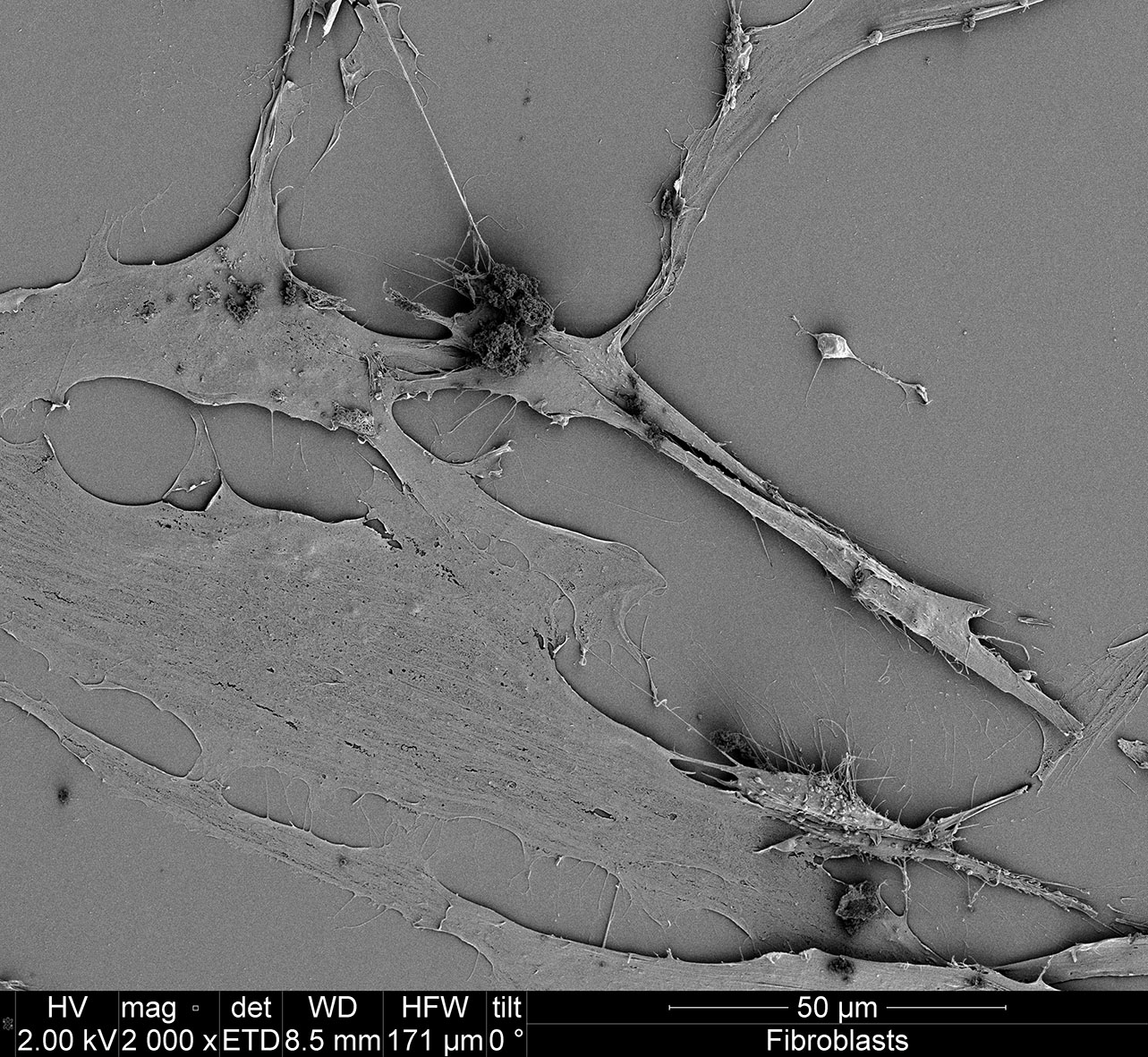

Supplement: Supplementary file 5 — Source data Fig. 3 [file 44321_2026_406_MOESM5_ESM.zip › Figure 3/3A/WT IR.tif]

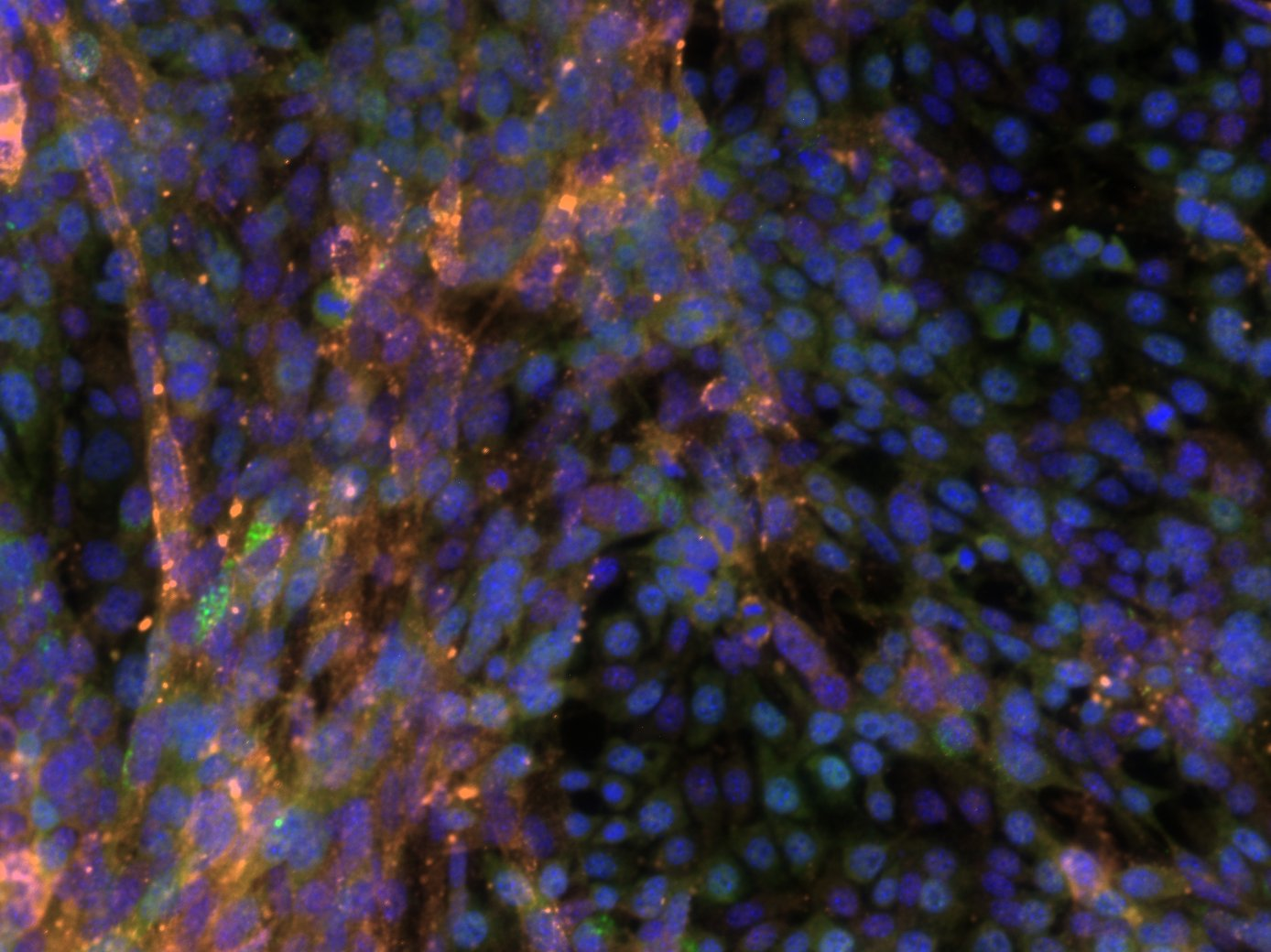

Supplement: Supplementary file 6 — Source data Fig. 4 [file 44321_2026_406_MOESM6_ESM.zip › Figure 4/Fig 4C/ko nir.tif]

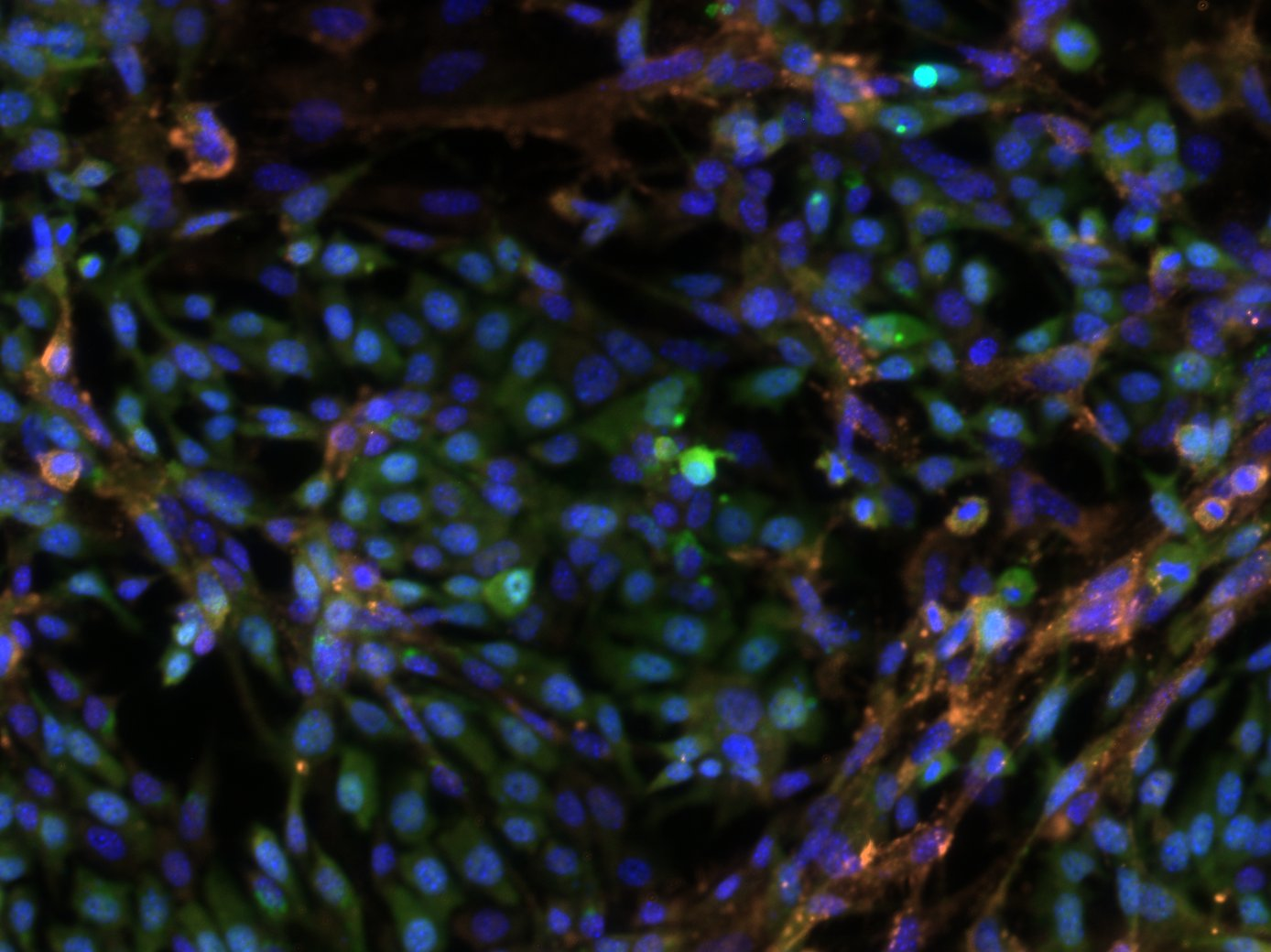

Supplement: Supplementary file 6 — Source data Fig. 4 [file 44321_2026_406_MOESM6_ESM.zip › Figure 4/Fig 4C/ko ir.tif]

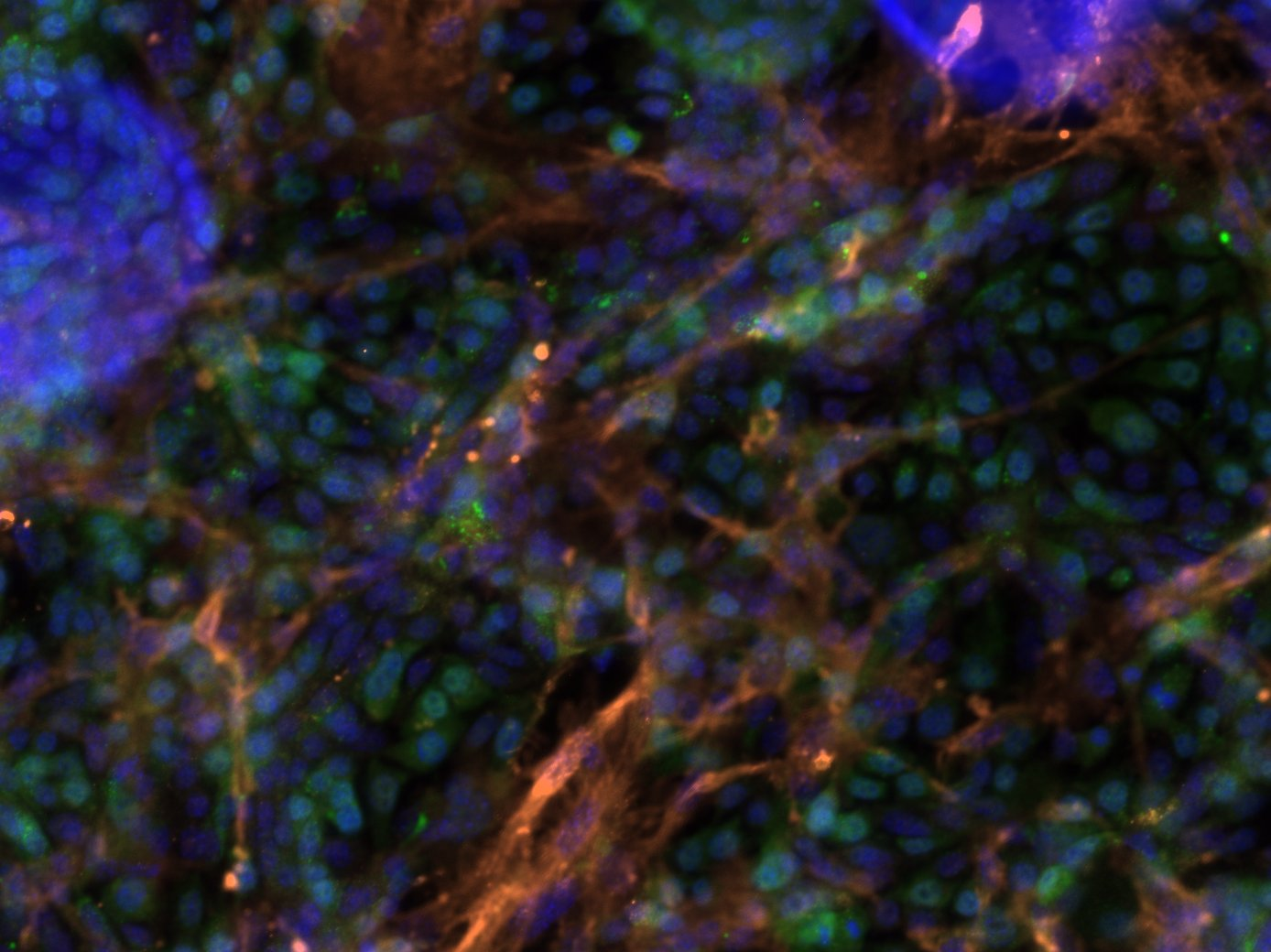

Supplement: Supplementary file 6 — Source data Fig. 4 [file 44321_2026_406_MOESM6_ESM.zip › Figure 4/Fig 4C/shtnc ir.tif]

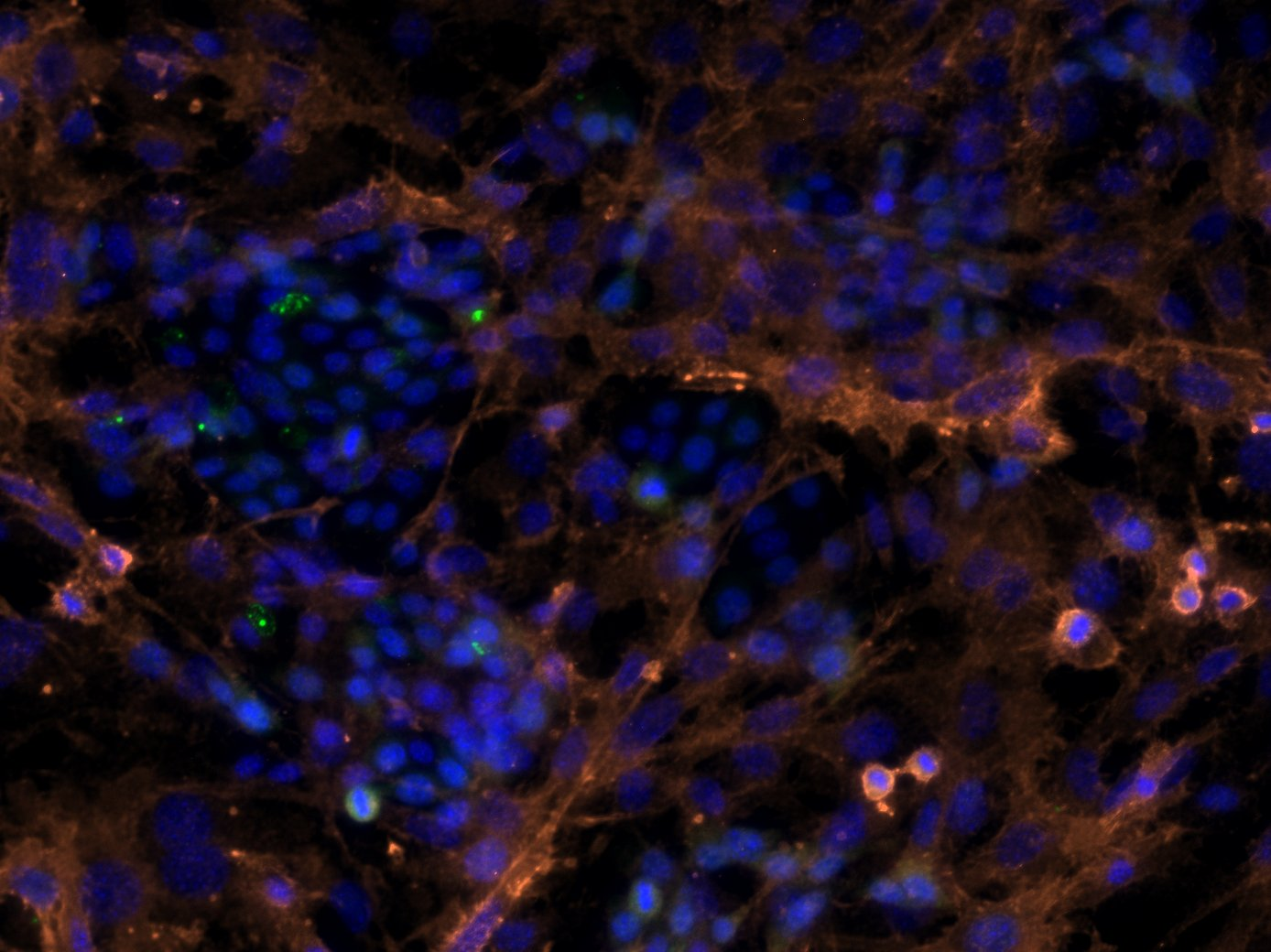

Supplement: Supplementary file 6 — Source data Fig. 4 [file 44321_2026_406_MOESM6_ESM.zip › Figure 4/Fig 4C/shC NIR.tif]

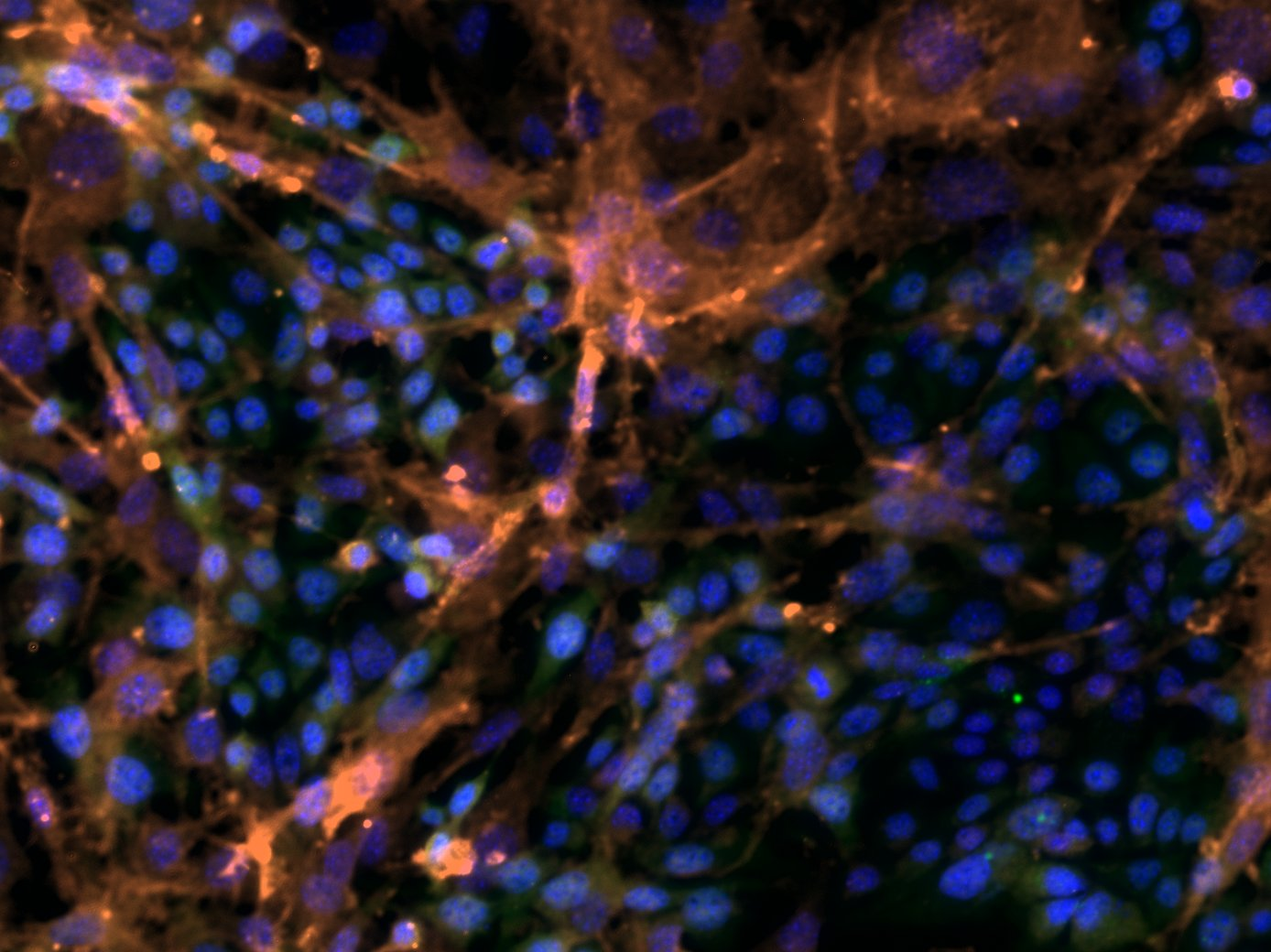

Supplement: Supplementary file 6 — Source data Fig. 4 [file 44321_2026_406_MOESM6_ESM.zip › Figure 4/Fig 4C/shtnc nir.tif]

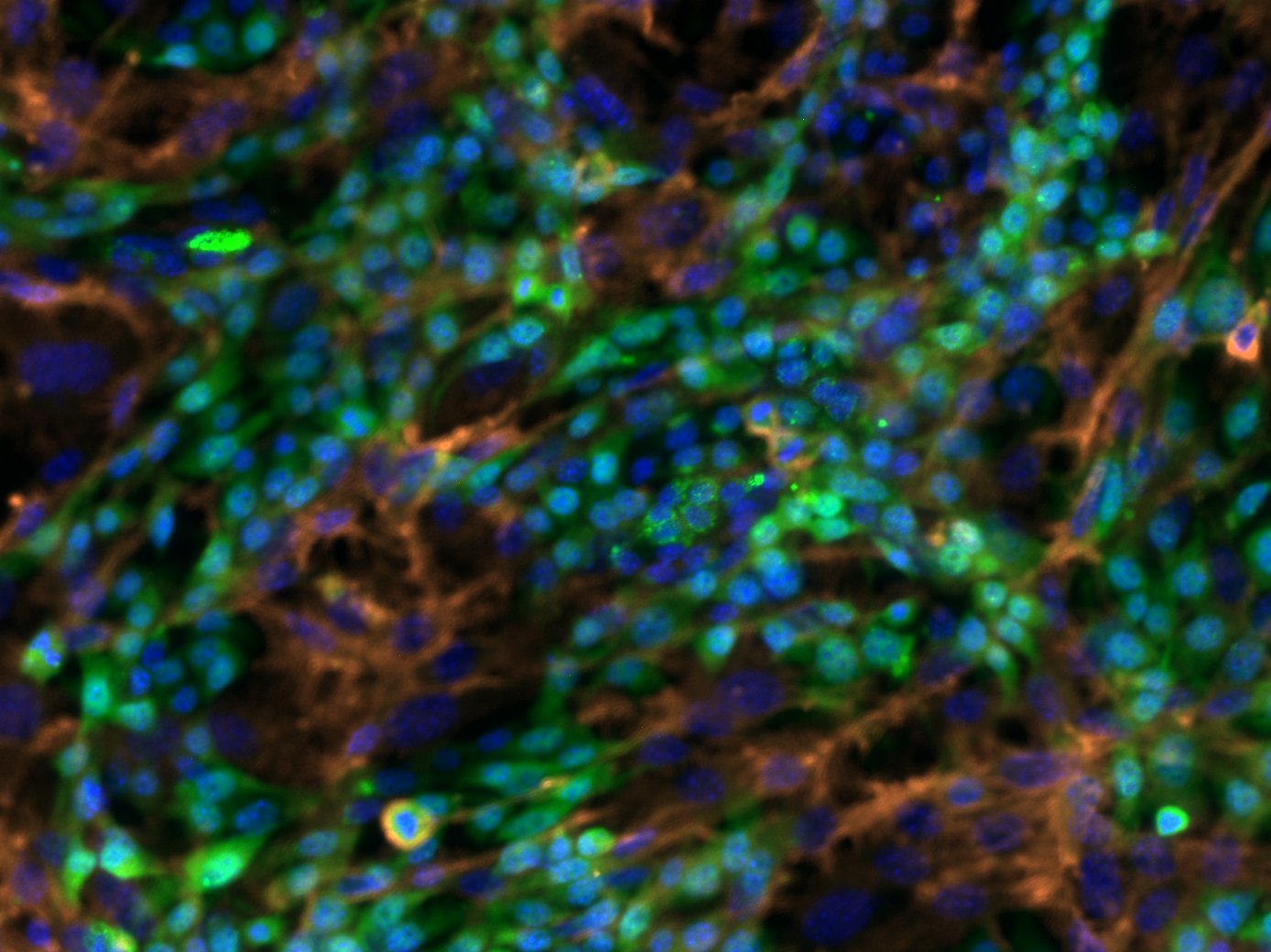

Supplement: Supplementary file 6 — Source data Fig. 4 [file 44321_2026_406_MOESM6_ESM.zip › Figure 4/Fig 4C/shc ir.tif]

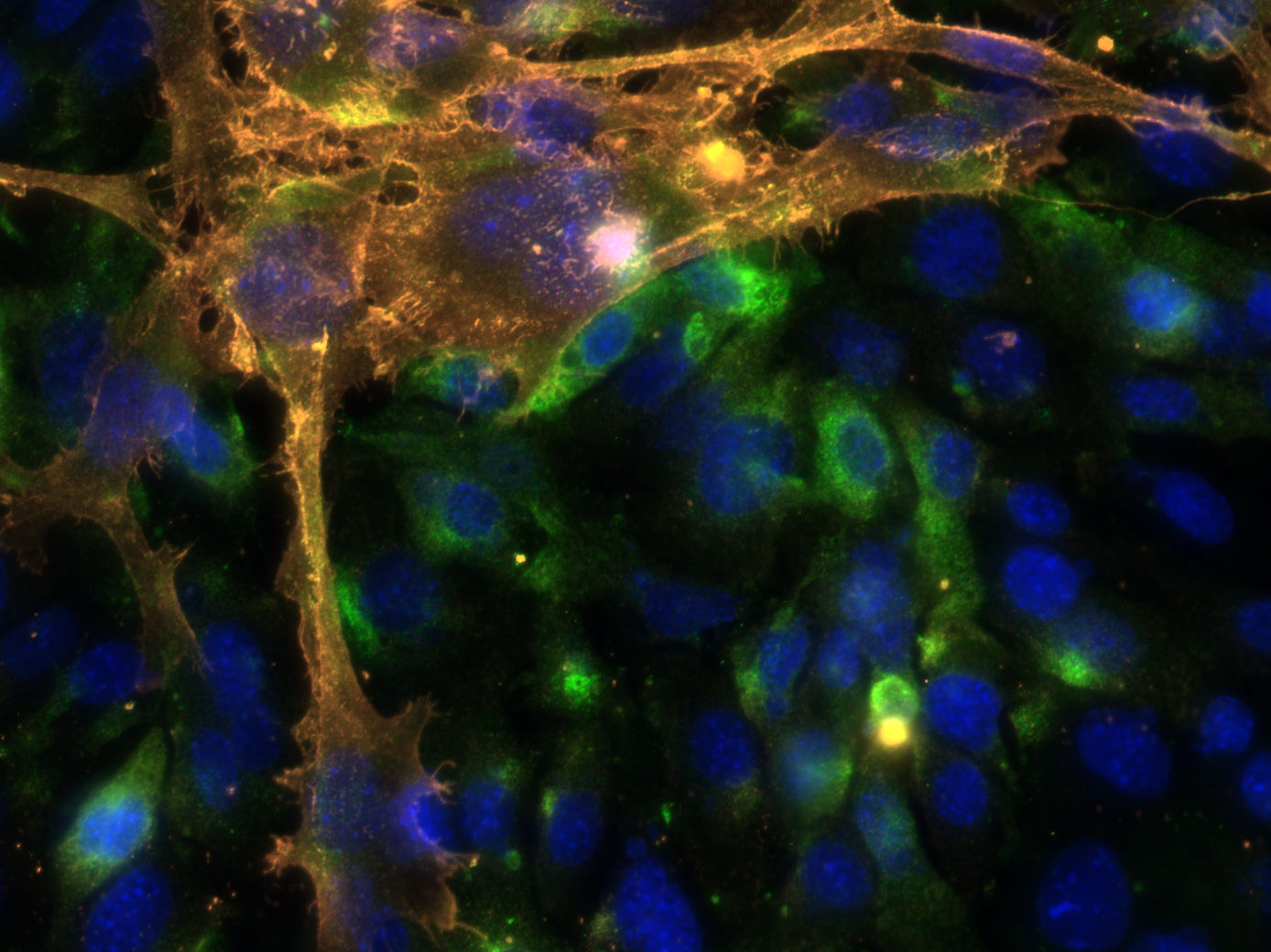

Supplement: Supplementary file 6 — Source data Fig. 4 [file 44321_2026_406_MOESM6_ESM.zip › Figure 4/Fig 4G/IR vim.tif]

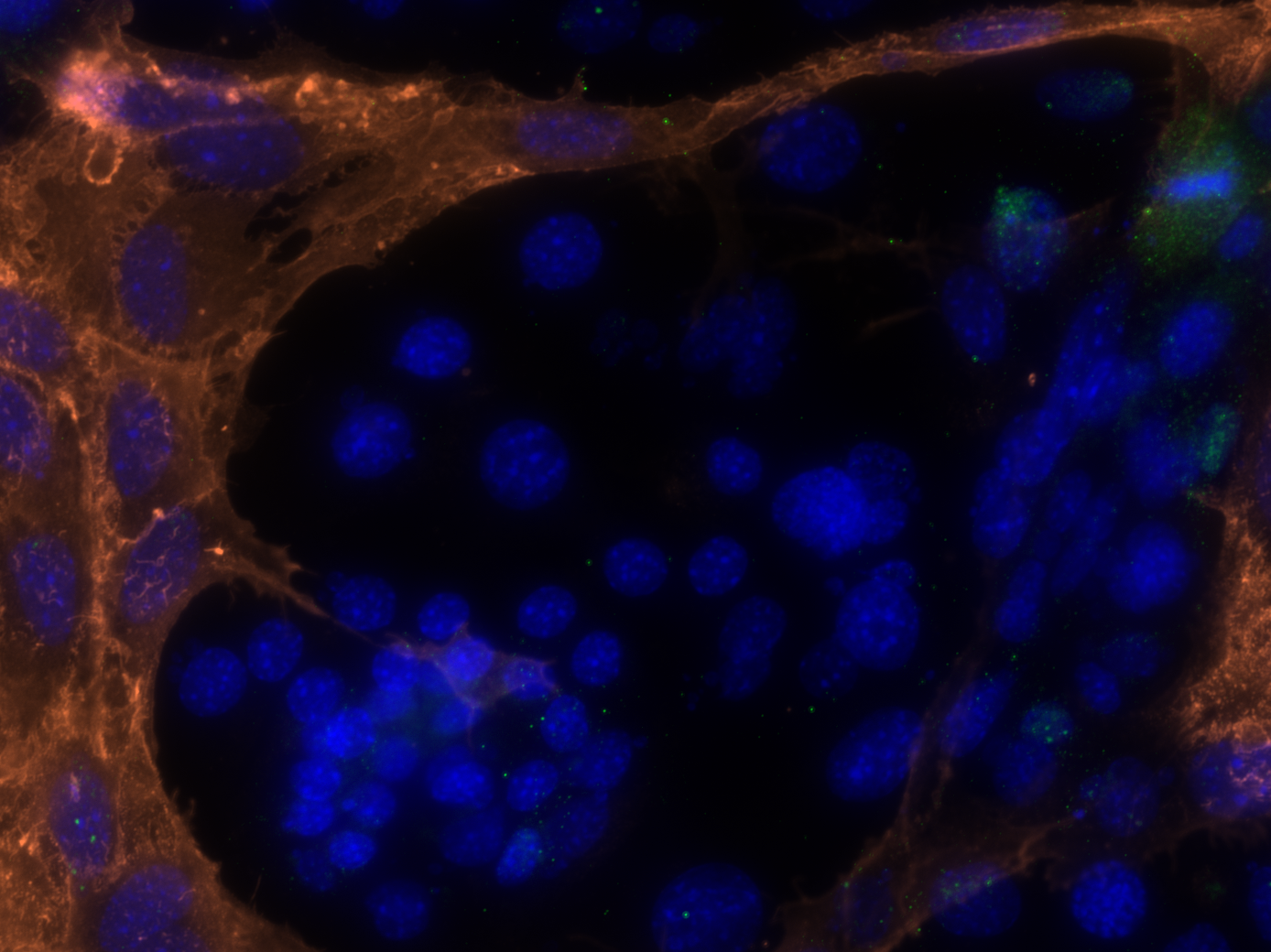

Supplement: Supplementary file 6 — Source data Fig. 4 [file 44321_2026_406_MOESM6_ESM.zip › Figure 4/Fig 4G/IR+ GW pSMAD.tif]

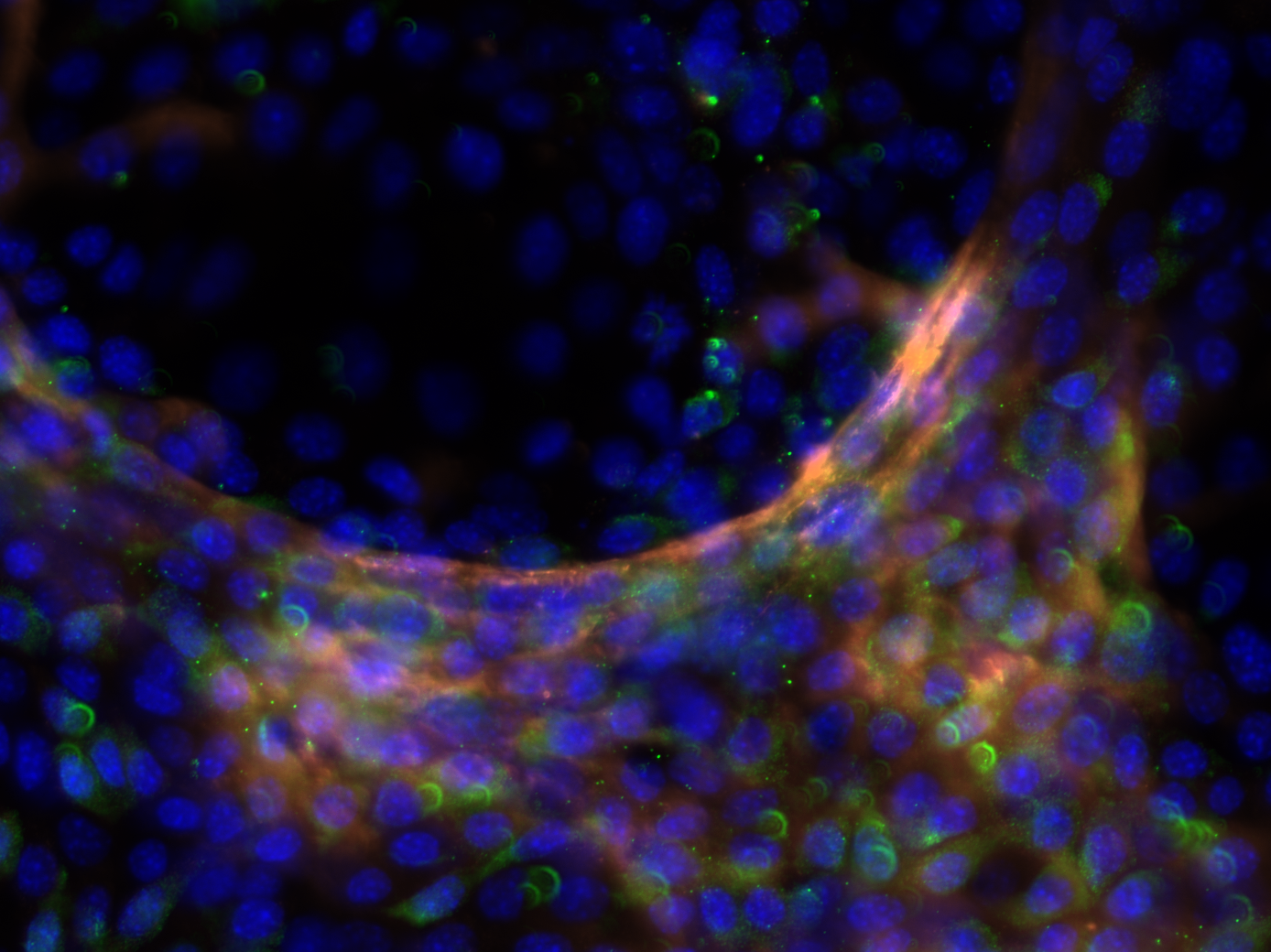

Supplement: Supplementary file 6 — Source data Fig. 4 [file 44321_2026_406_MOESM6_ESM.zip › Figure 4/Fig 4G/NIR vimentin.tif]

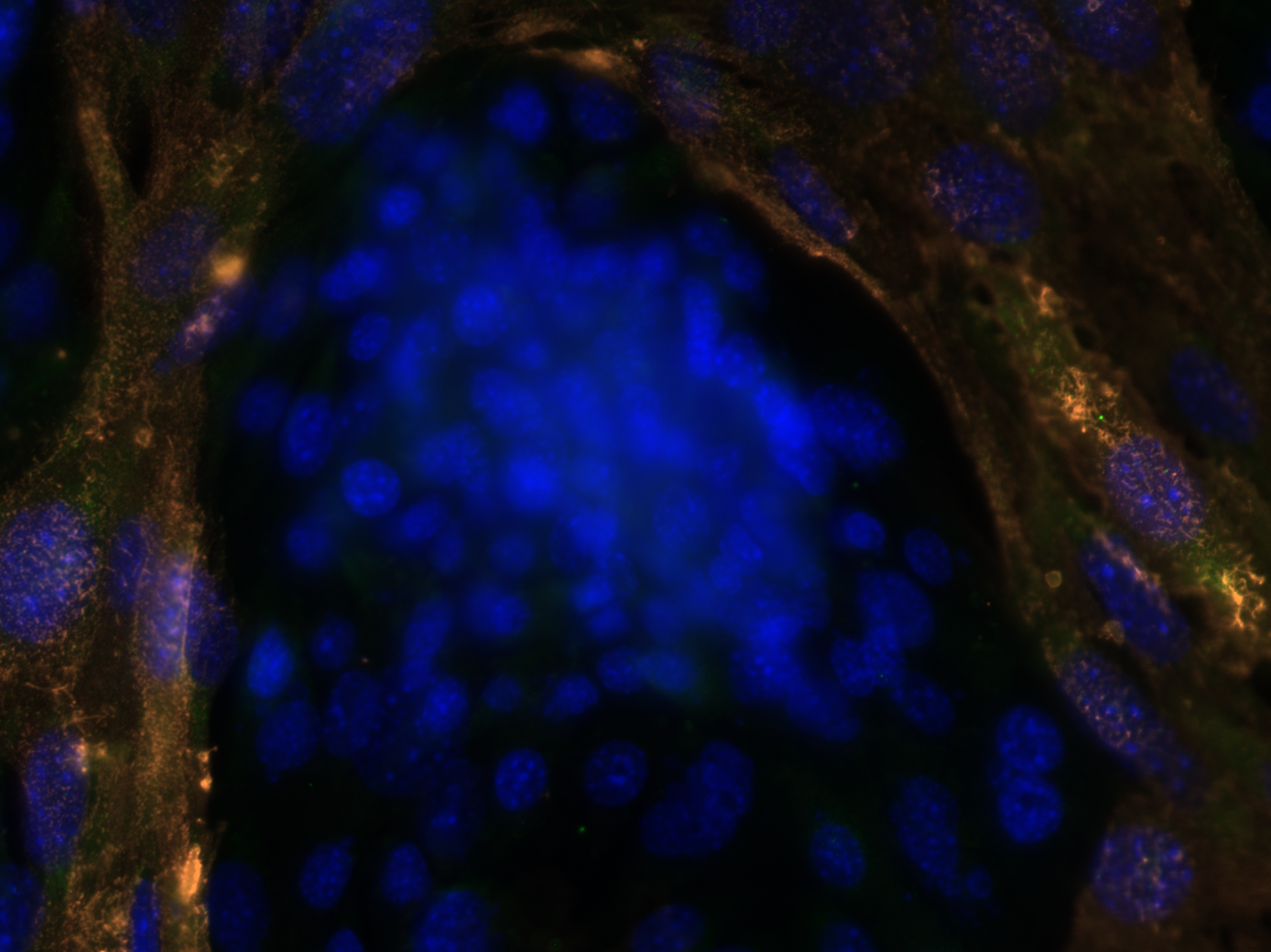

Supplement: Supplementary file 6 — Source data Fig. 4 [file 44321_2026_406_MOESM6_ESM.zip › Figure 4/Fig 4G/IR + GW vimentin.tif]

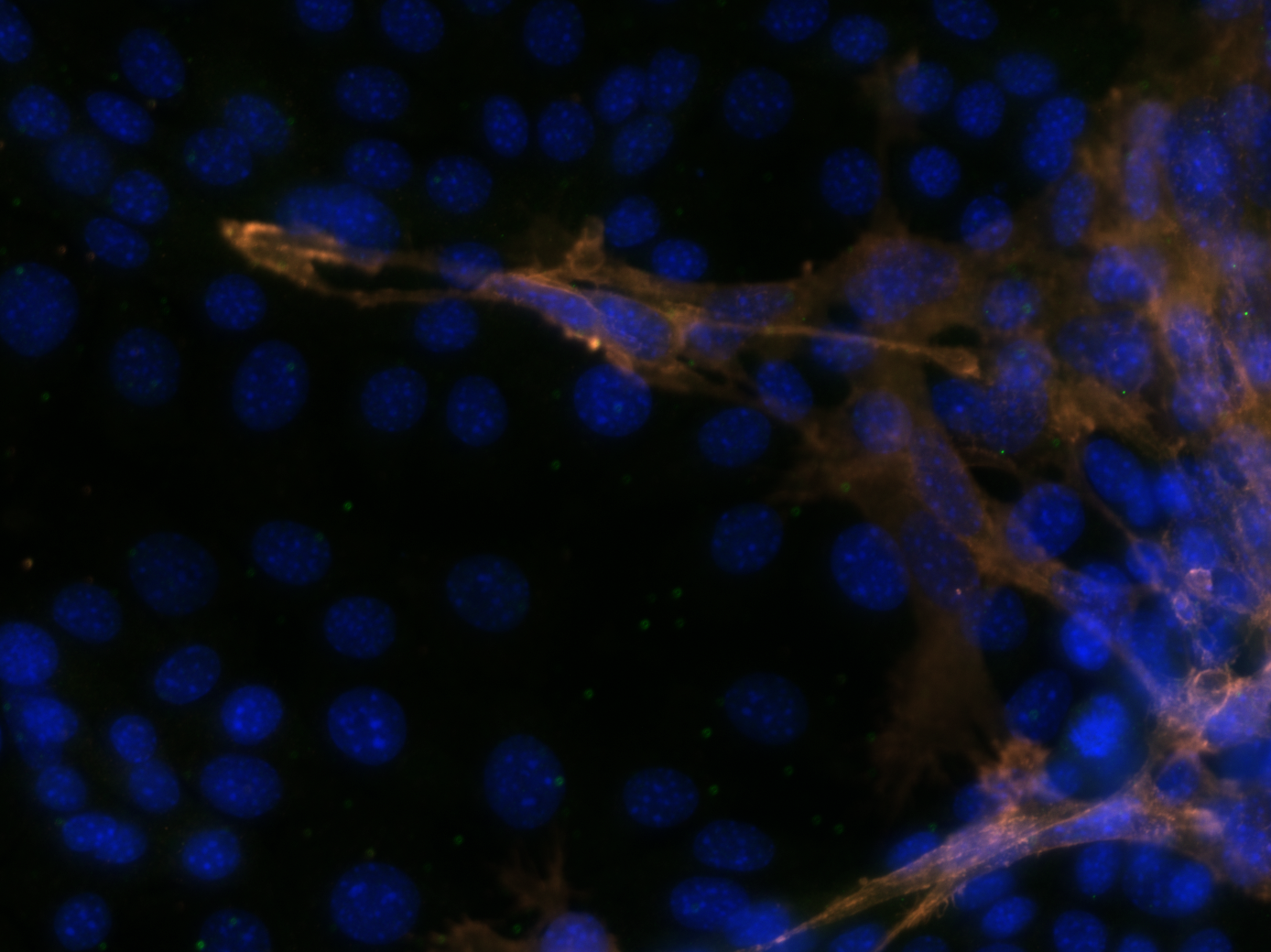

Supplement: Supplementary file 6 — Source data Fig. 4 [file 44321_2026_406_MOESM6_ESM.zip › Figure 4/Fig 4G/NIR pSMAD.tif]

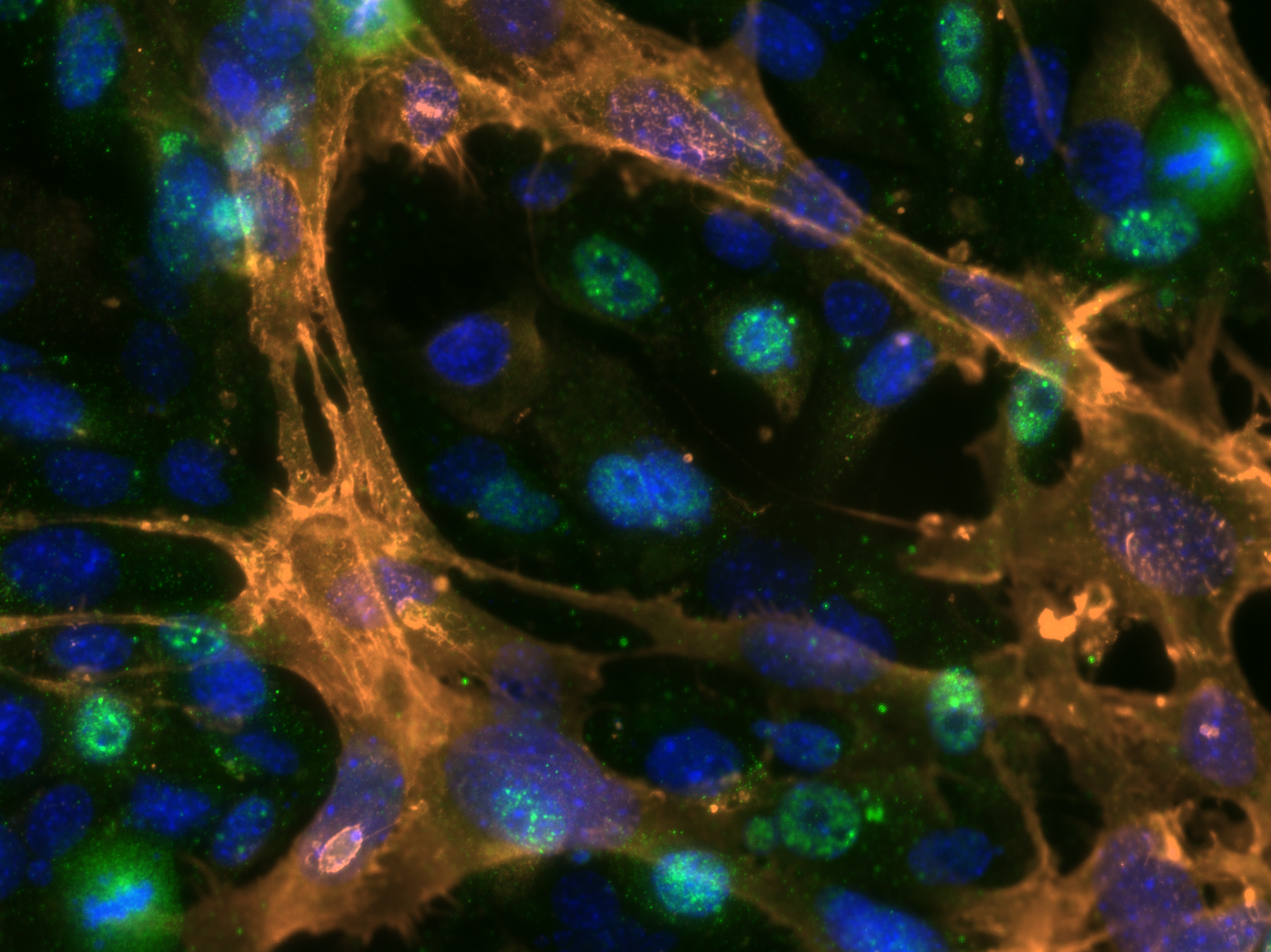

Supplement: Supplementary file 6 — Source data Fig. 4 [file 44321_2026_406_MOESM6_ESM.zip › Figure 4/Fig 4G/IR pSMAD.tif]

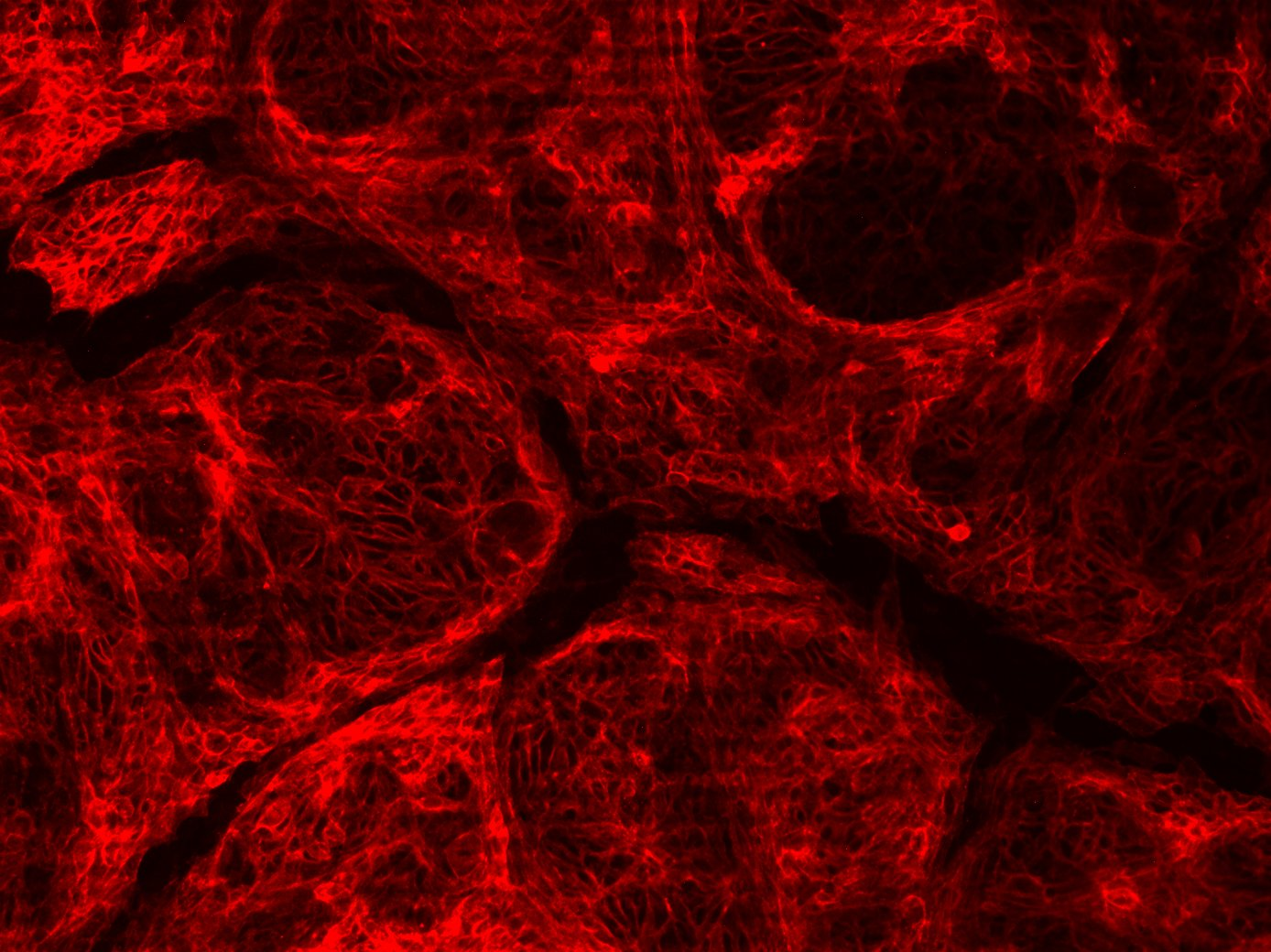

Supplement: Supplementary file 6 — Source data Fig. 4 [file 44321_2026_406_MOESM6_ESM.zip › Figure 4/Fig 4A/OSCC13-FRCWTShC bECad.tif]

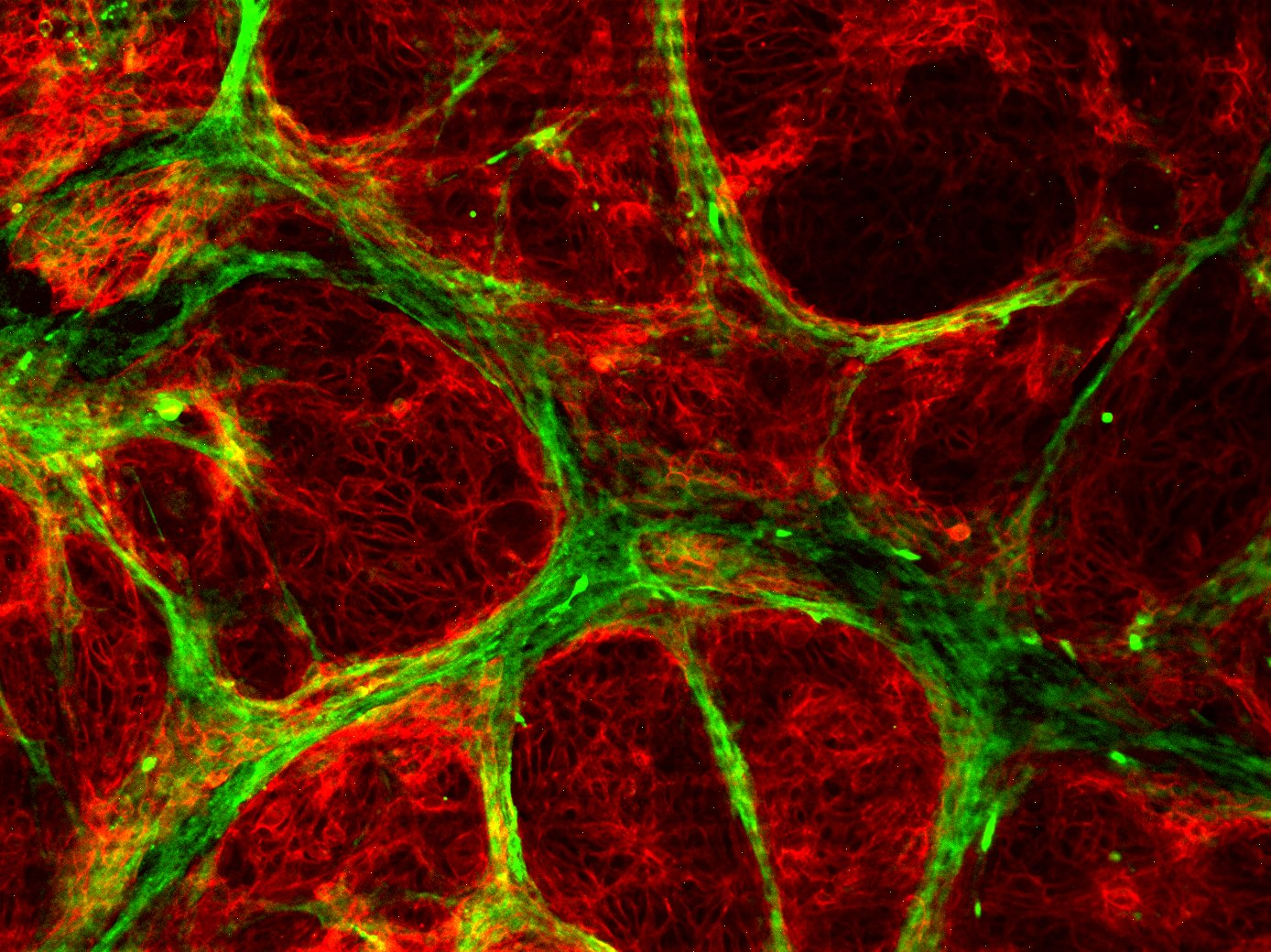

Supplement: Supplementary file 6 — Source data Fig. 4 [file 44321_2026_406_MOESM6_ESM.zip › Figure 4/Fig 4A/Composite_OSCC FRC shc ECad-ITGA7.tif]

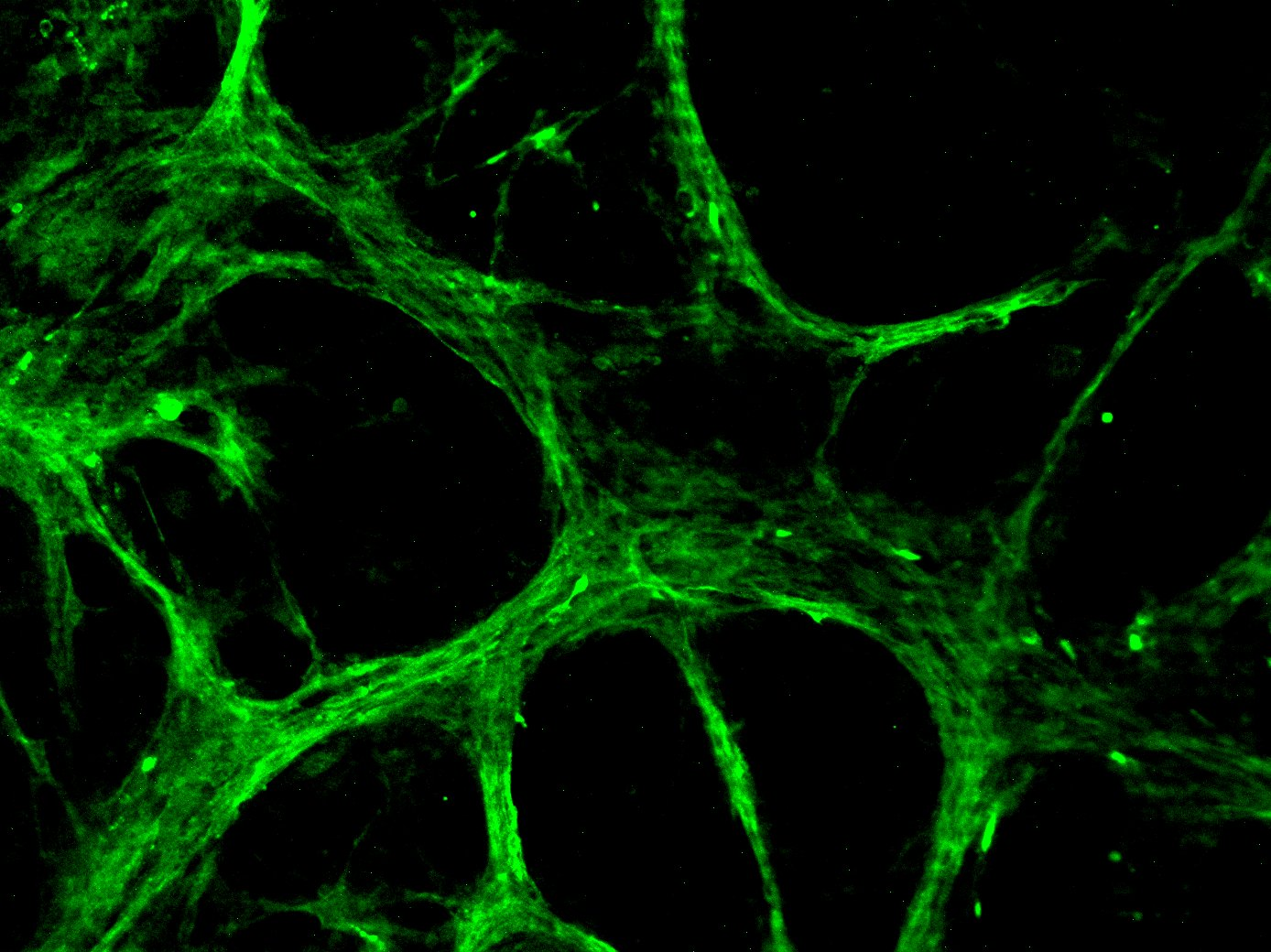

Supplement: Supplementary file 6 — Source data Fig. 4 [file 44321_2026_406_MOESM6_ESM.zip › Figure 4/Fig 4A/OSCC13-FRCWTShCITGA7.tif]

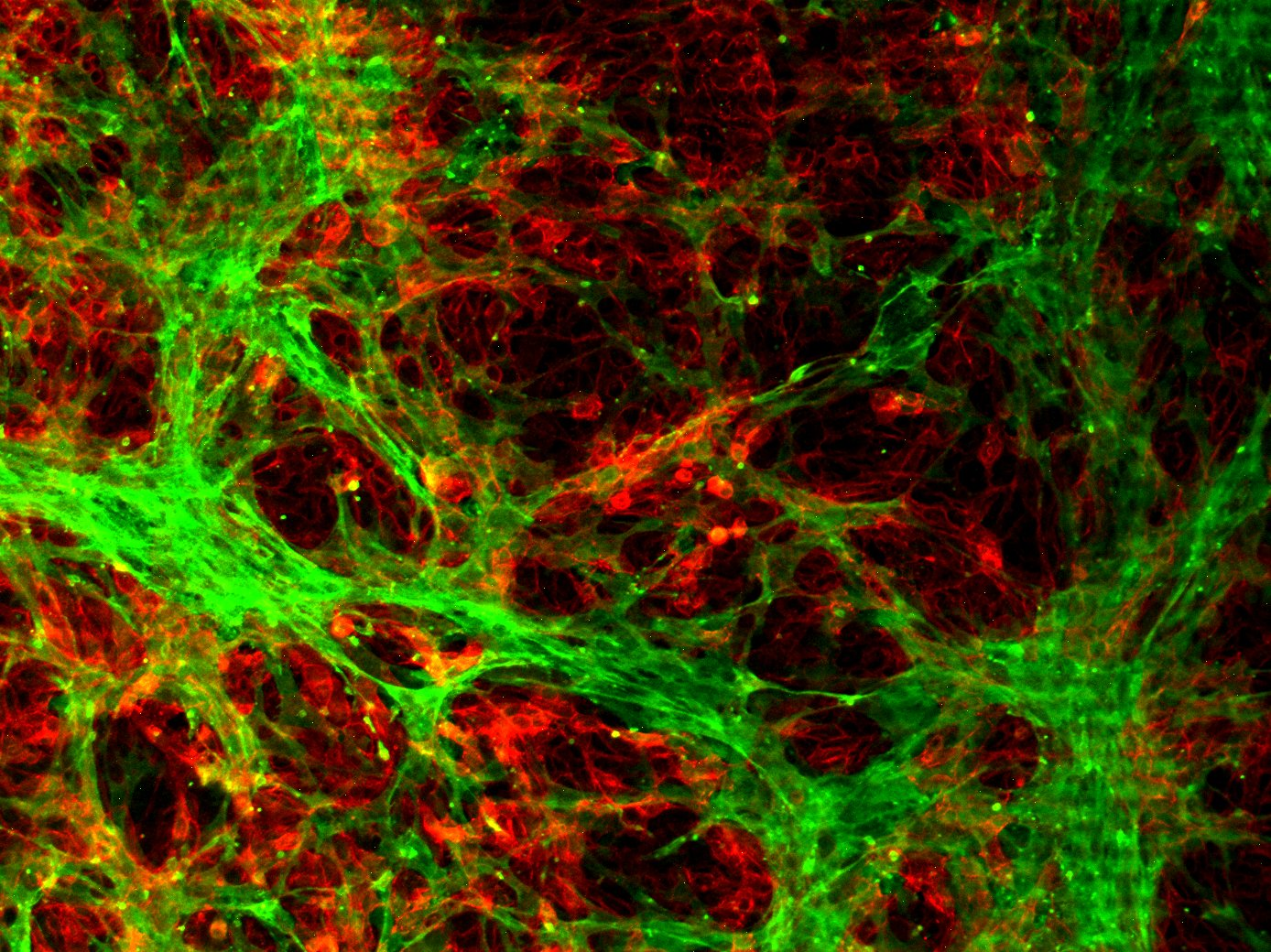

Supplement: Supplementary file 6 — Source data Fig. 4 [file 44321_2026_406_MOESM6_ESM.zip › Figure 4/Fig 4A/Composite_OSCC FRC KO ECad-ITGA7.tif]

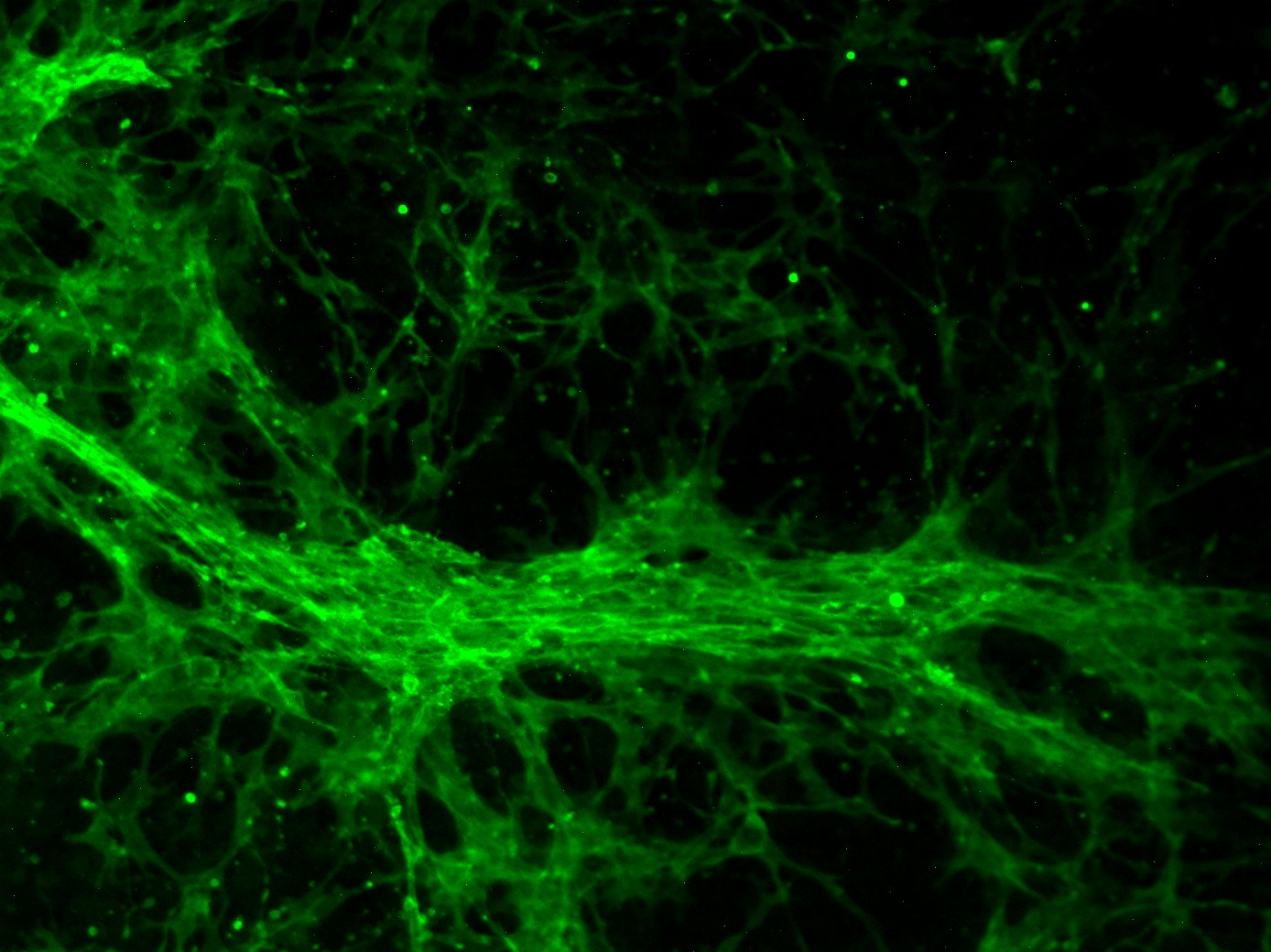

Supplement: Supplementary file 6 — Source data Fig. 4 [file 44321_2026_406_MOESM6_ESM.zip › Figure 4/Fig 4A/OSCC13-FRCKOITGA7.tif]

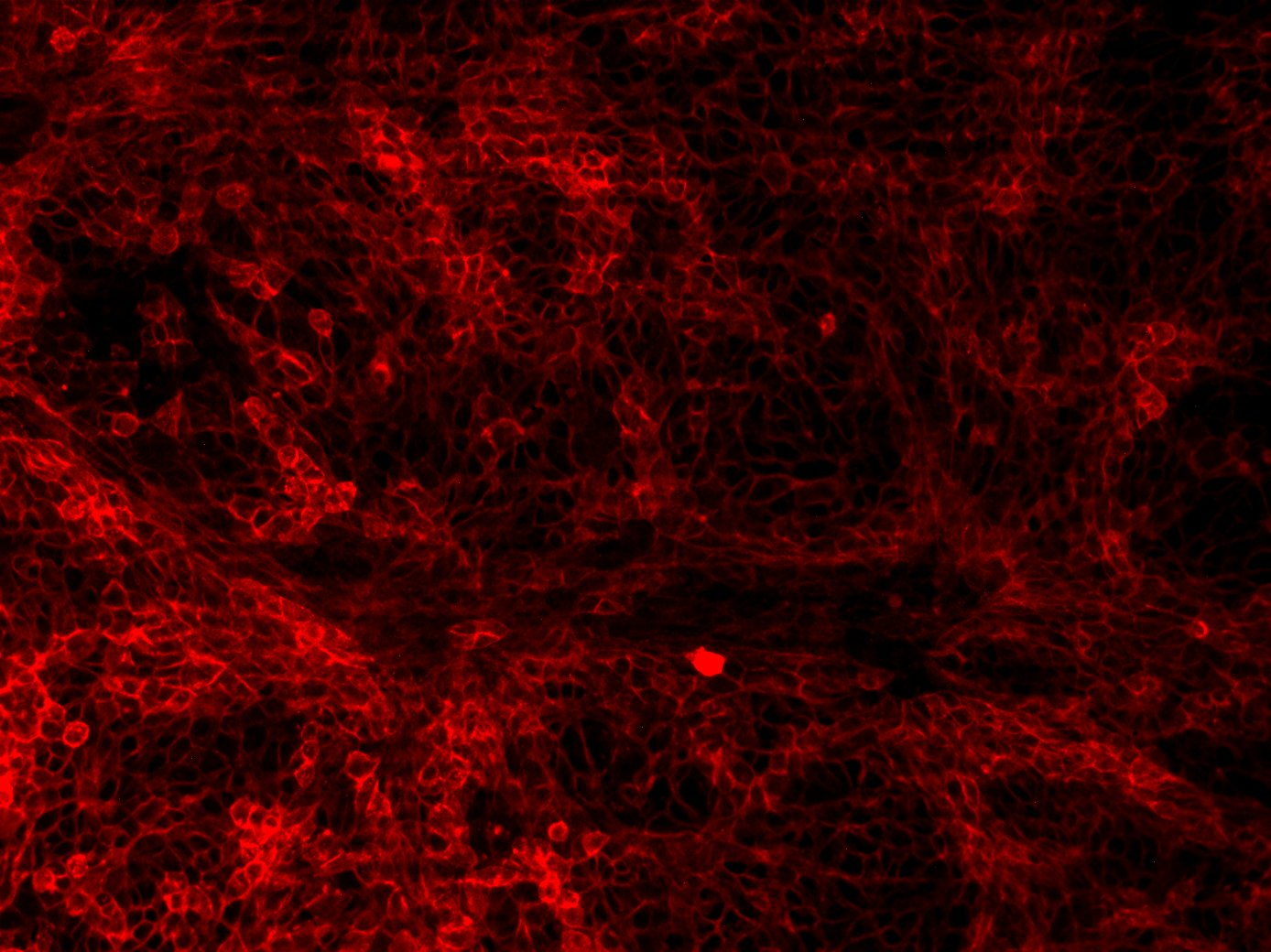

Supplement: Supplementary file 6 — Source data Fig. 4 [file 44321_2026_406_MOESM6_ESM.zip › Figure 4/Fig 4A/OSCC13-FRCKO ECad.tif]

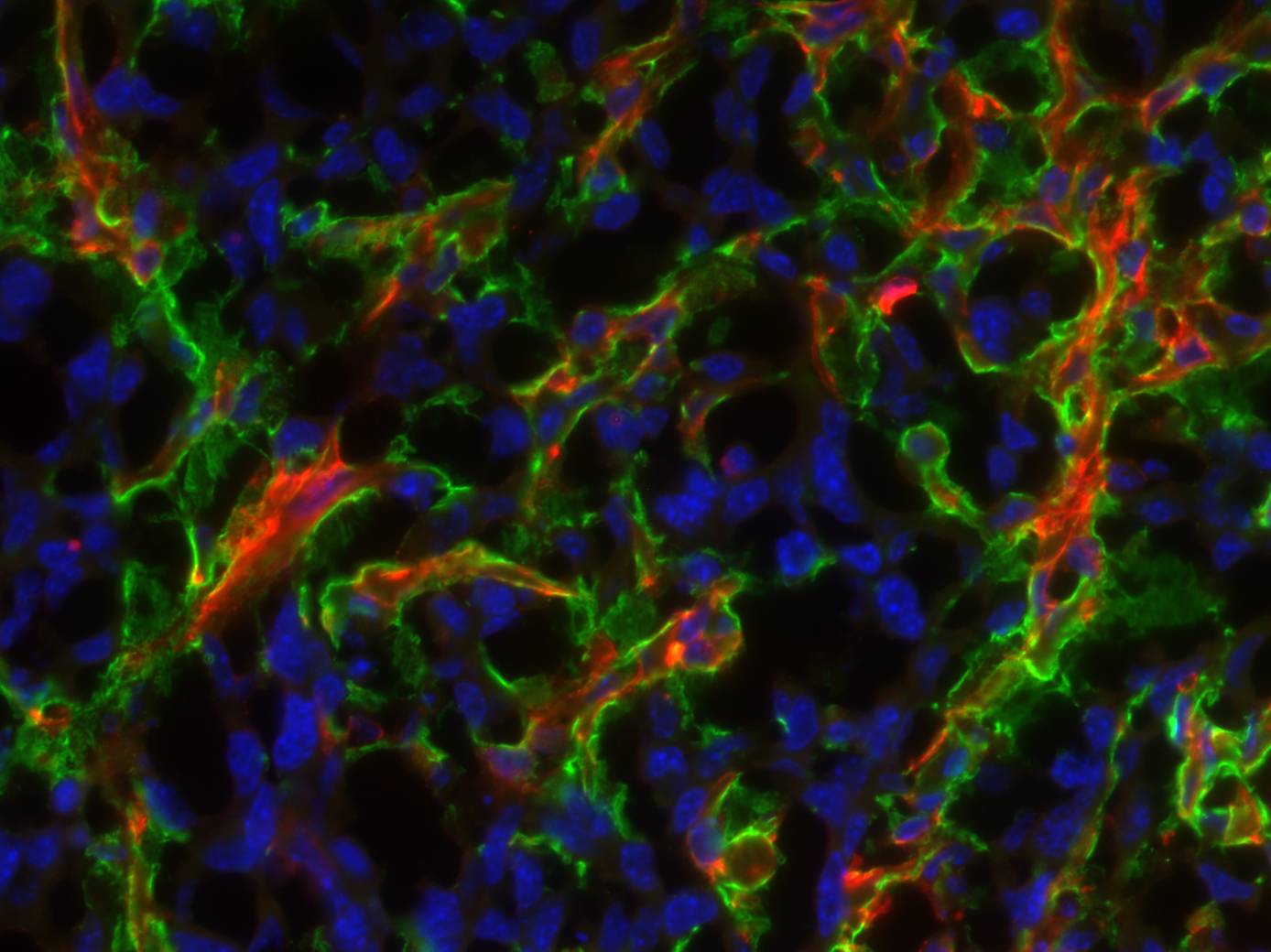

Supplement: Supplementary file 7 — Source data Fig. 5 [file 44321_2026_406_MOESM7_ESM.zip › Figure 5/Fig 5E/OSCC FRC WT GP38.tif]

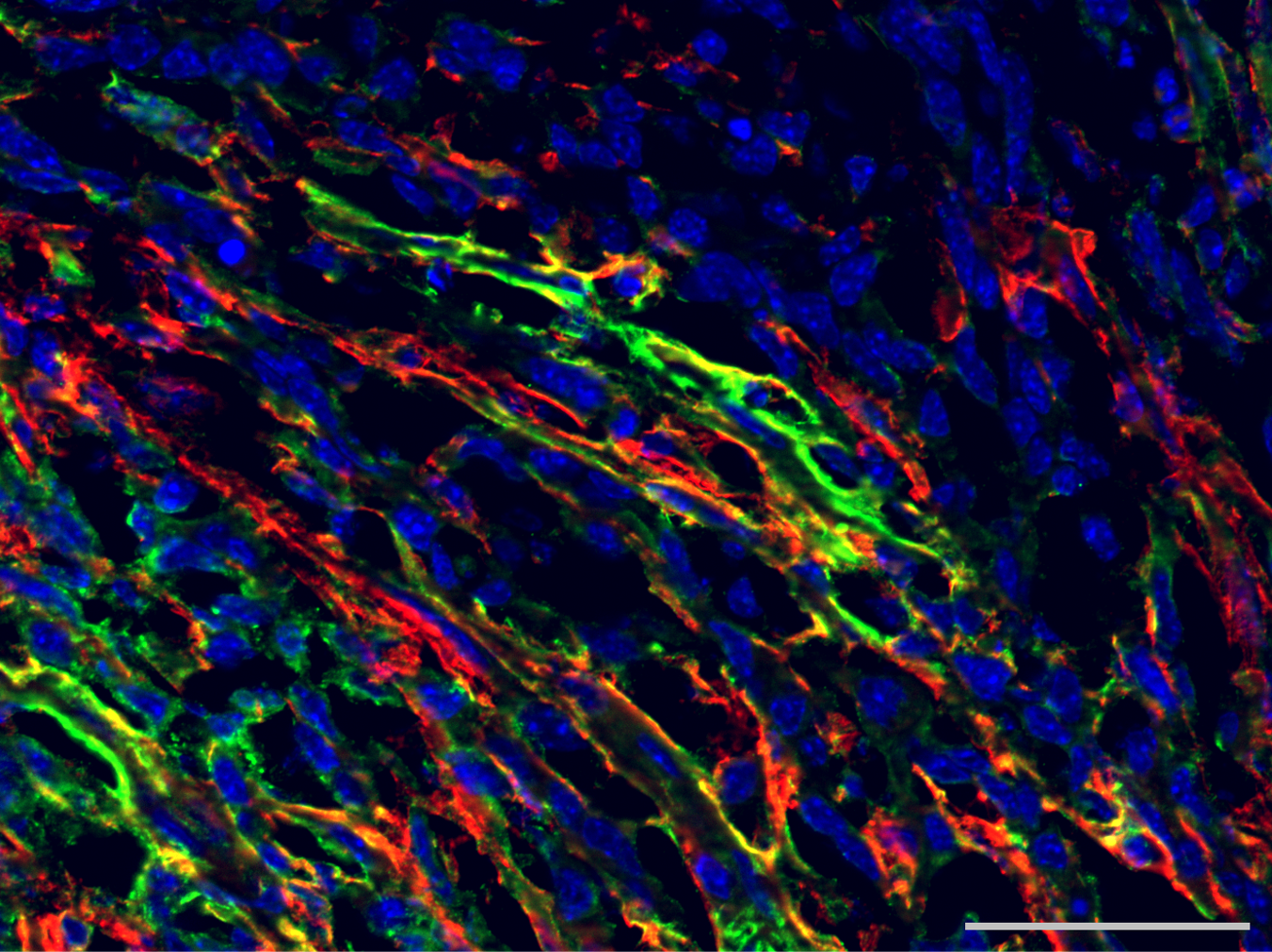

Supplement: Supplementary file 7 — Source data Fig. 5 [file 44321_2026_406_MOESM7_ESM.zip › Figure 5/Fig 5E/OSCC FRCWT LM.tif]

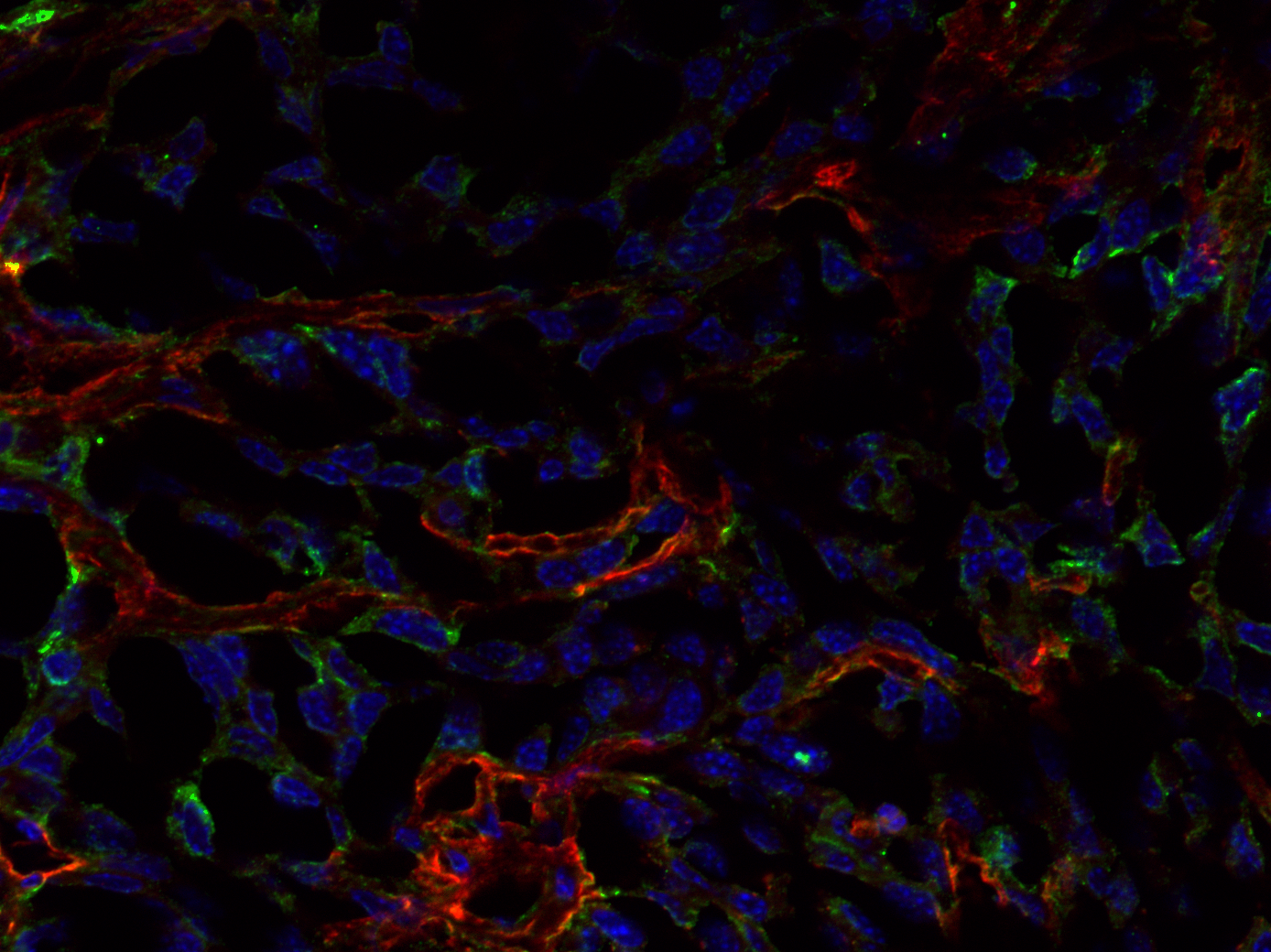

Supplement: Supplementary file 7 — Source data Fig. 5 [file 44321_2026_406_MOESM7_ESM.zip › Figure 5/Fig 5E/OSCC FRCKO LM.tif]

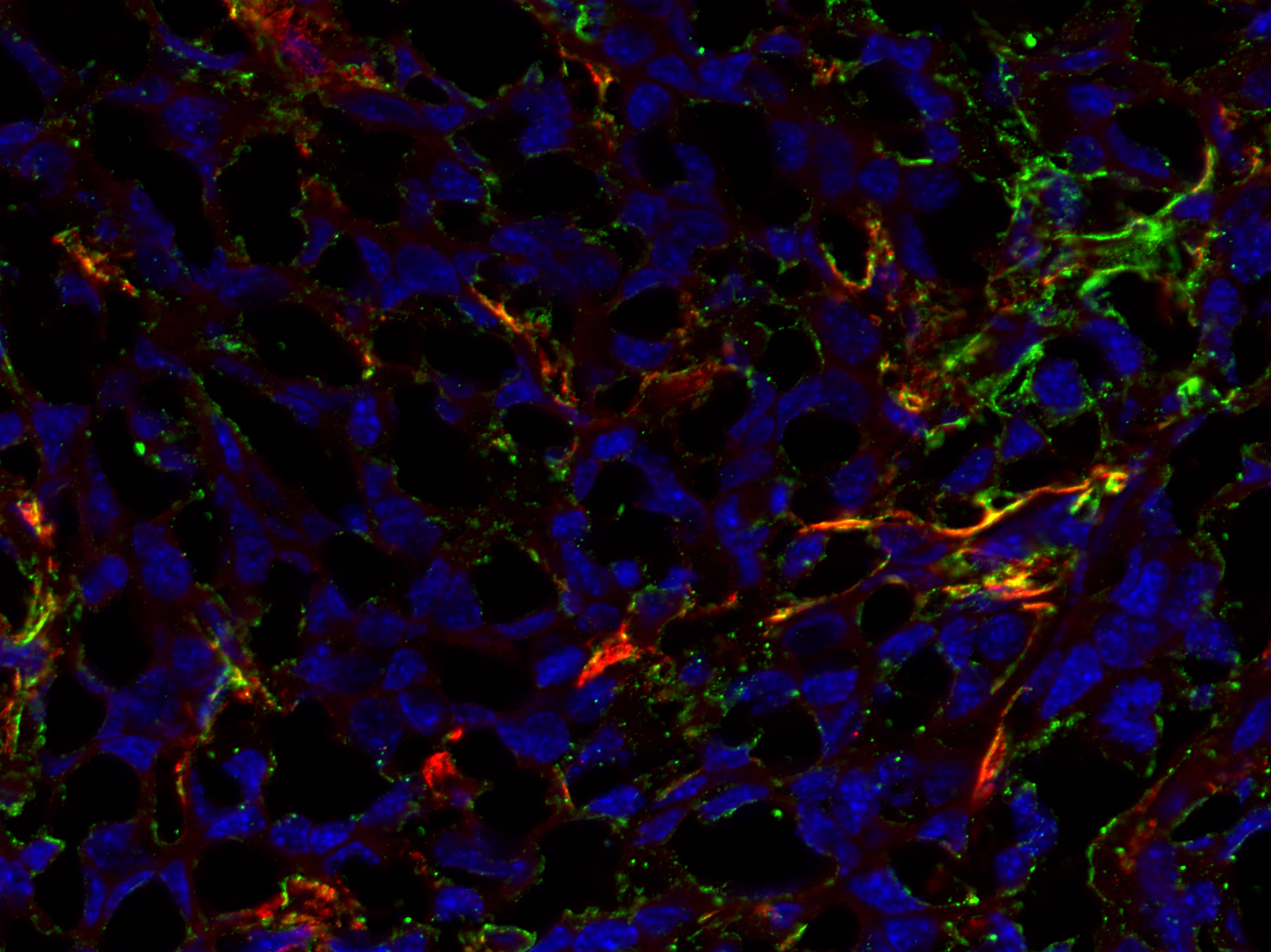

Supplement: Supplementary file 7 — Source data Fig. 5 [file 44321_2026_406_MOESM7_ESM.zip › Figure 5/Fig 5E/OSCC LM.tif]

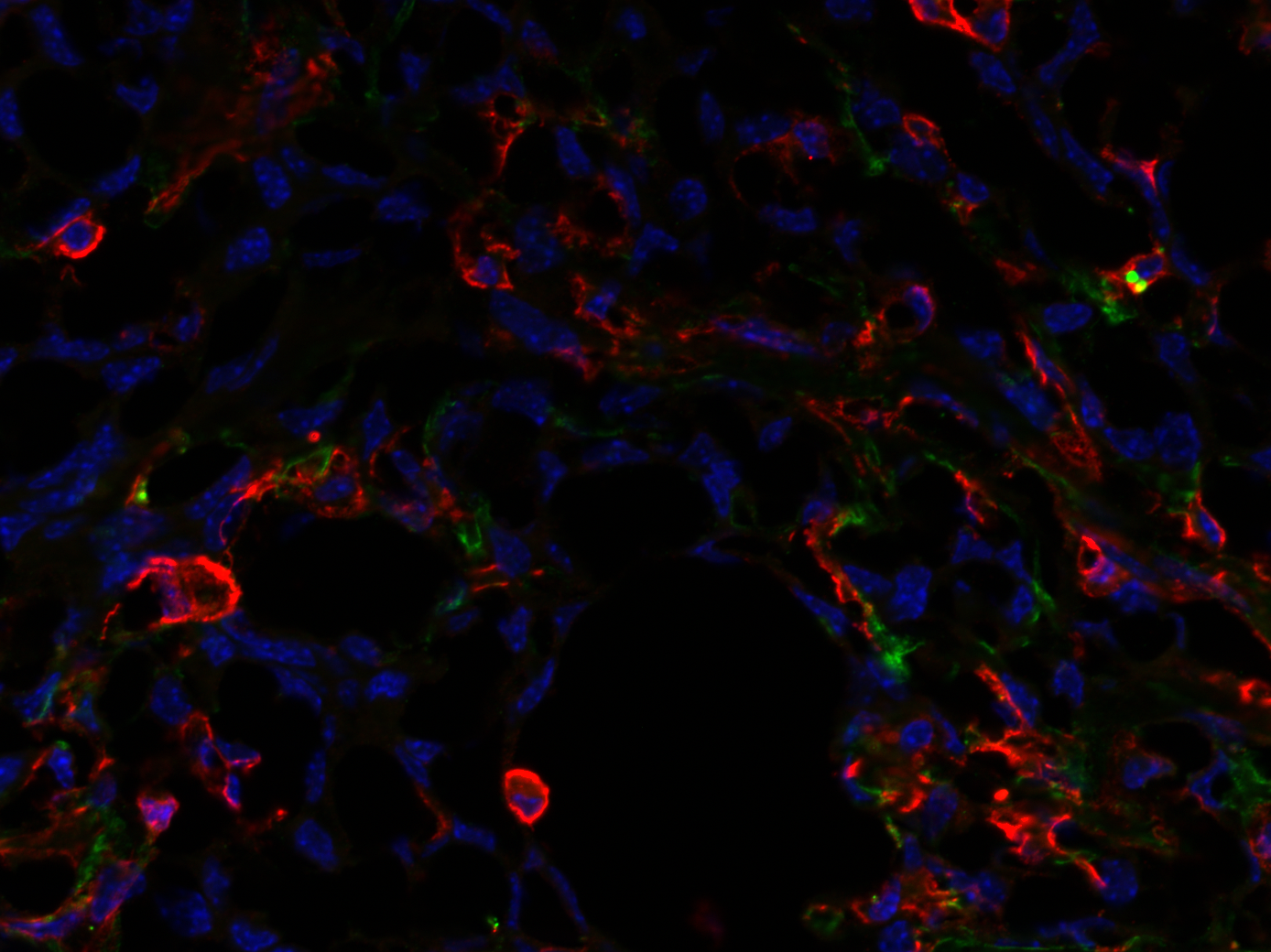

Supplement: Supplementary file 7 — Source data Fig. 5 [file 44321_2026_406_MOESM7_ESM.zip › Figure 5/Fig 5E/OSCC FRCKO GP38.tif]

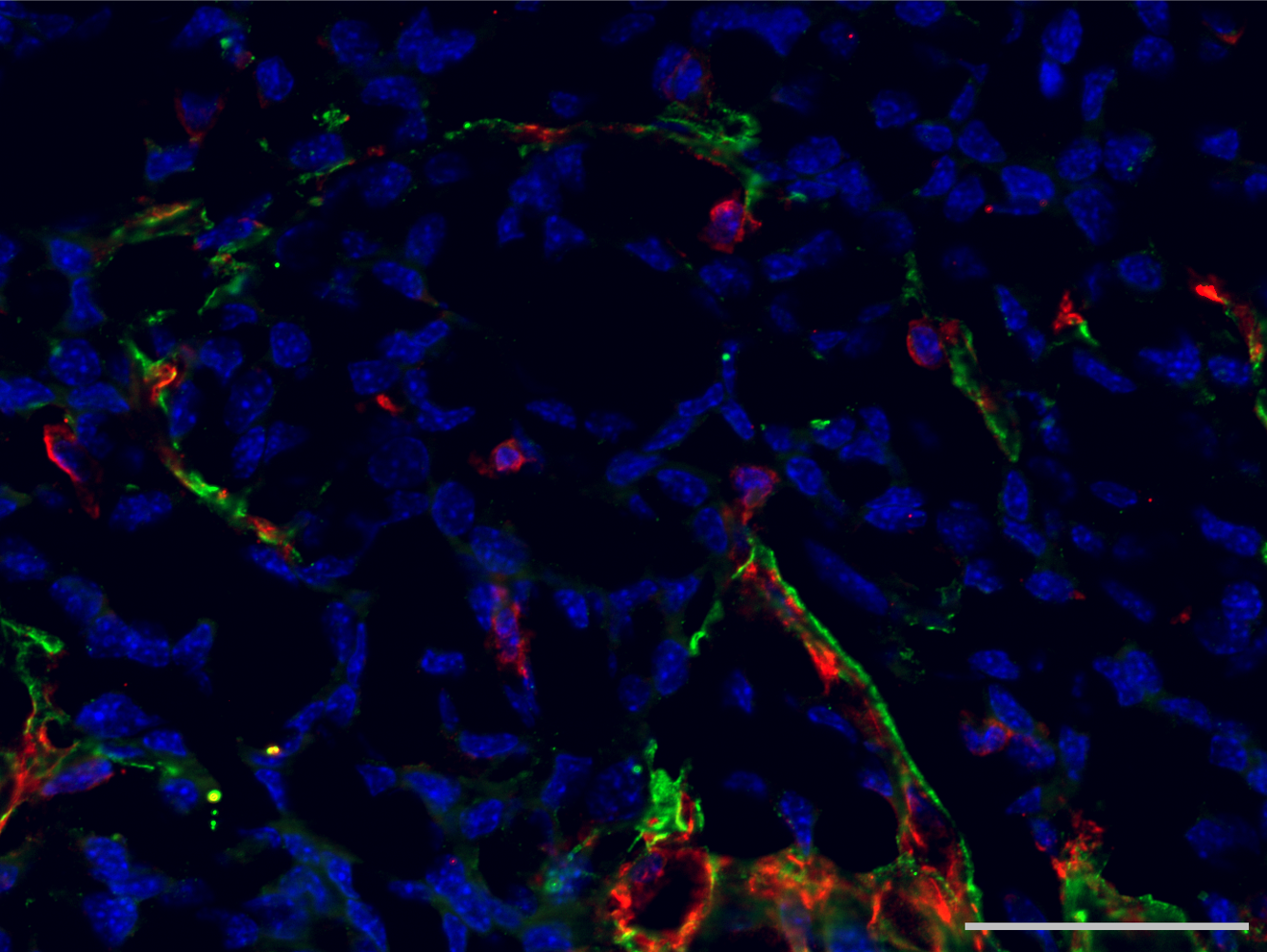

Supplement: Supplementary file 7 — Source data Fig. 5 [file 44321_2026_406_MOESM7_ESM.zip › Figure 5/Fig 5E/OSCC GP38.tif]

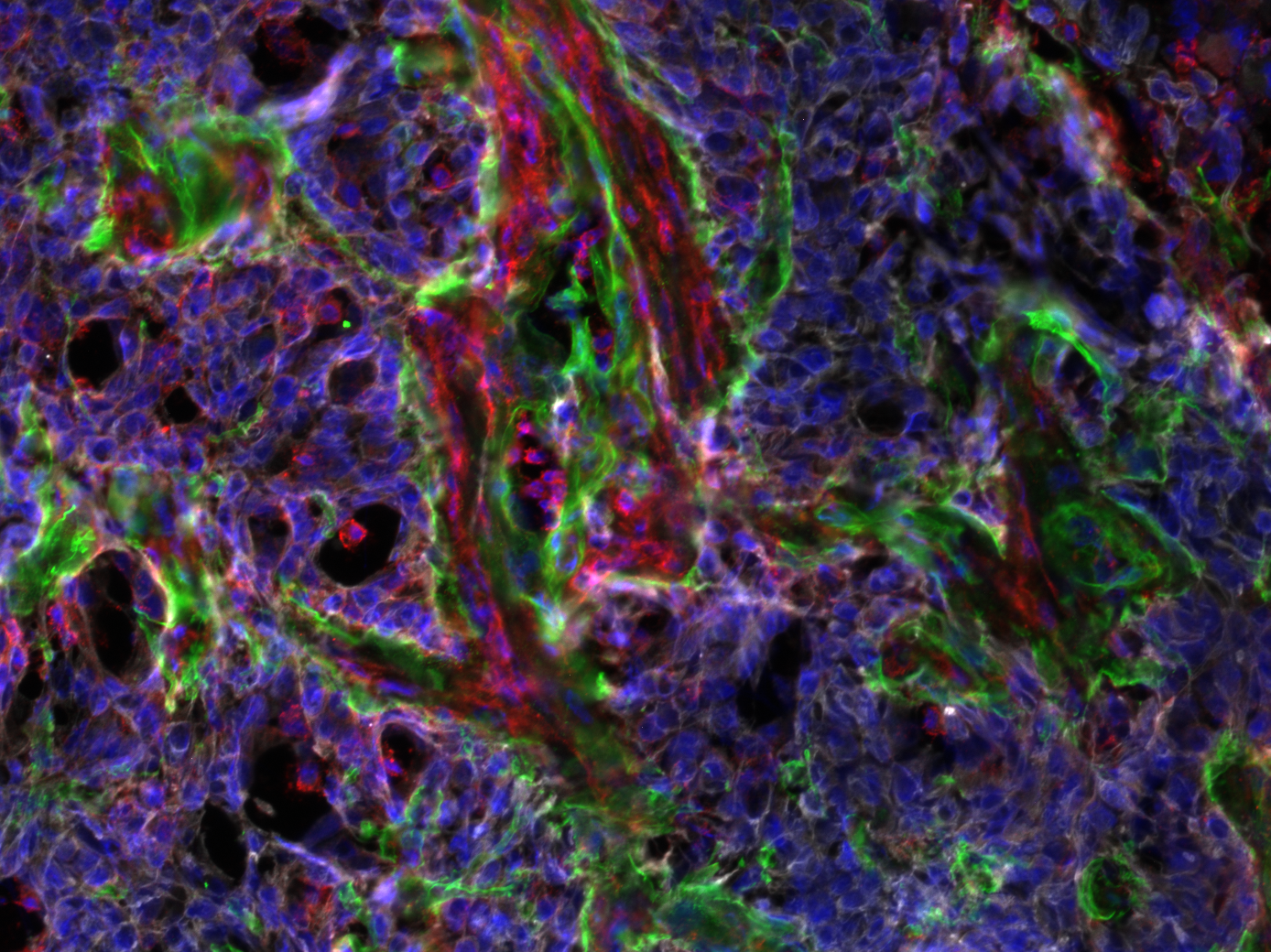

Supplement: Supplementary file 7 — Source data Fig. 5 [file 44321_2026_406_MOESM7_ESM.zip › Figure 5/Fig 5B/OSCC FRC CD45.tif]

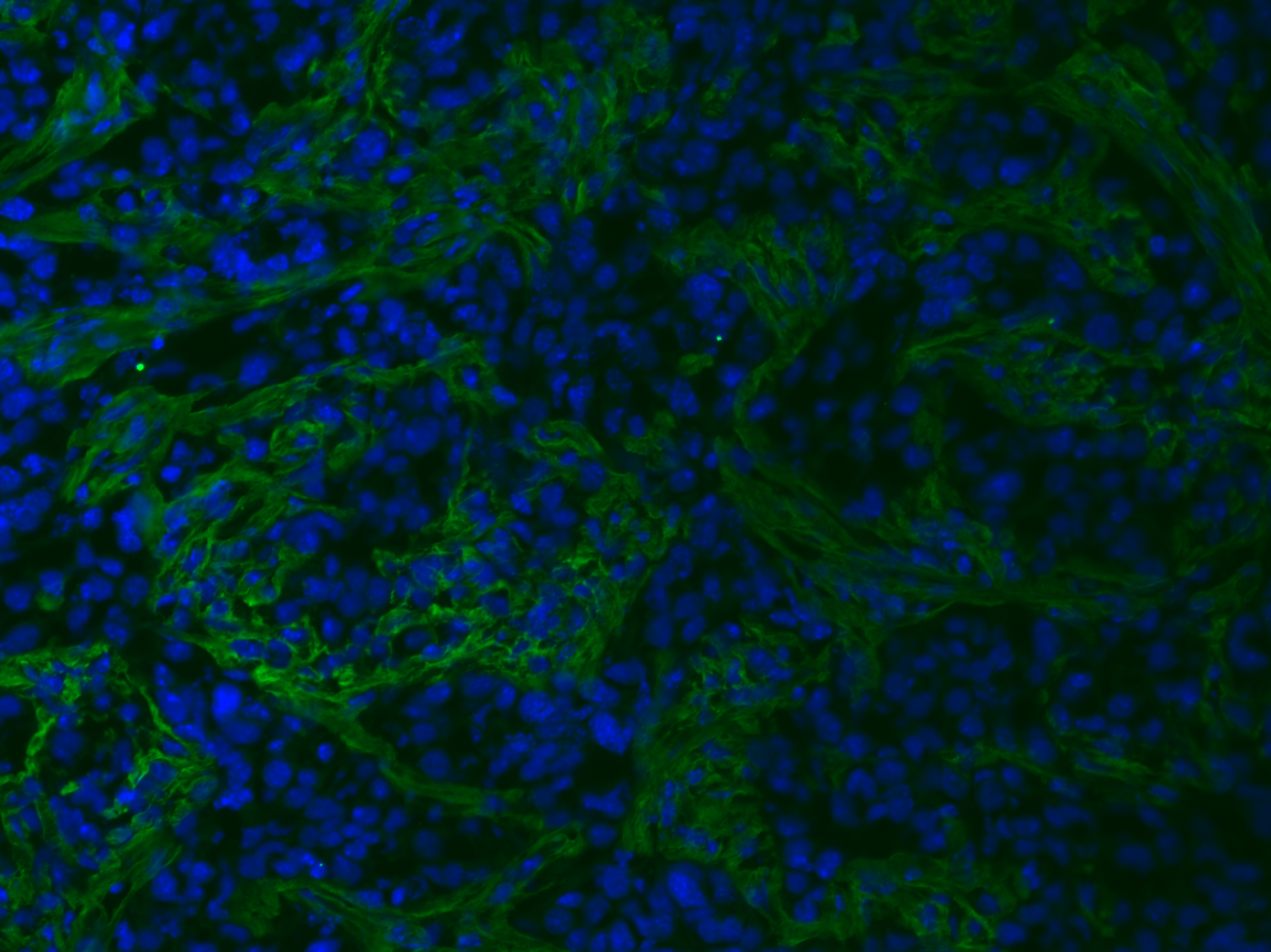

Supplement: Supplementary file 7 — Source data Fig. 5 [file 44321_2026_406_MOESM7_ESM.zip › Figure 5/Fig 5B/OSCC FRC ERTR7.tif]

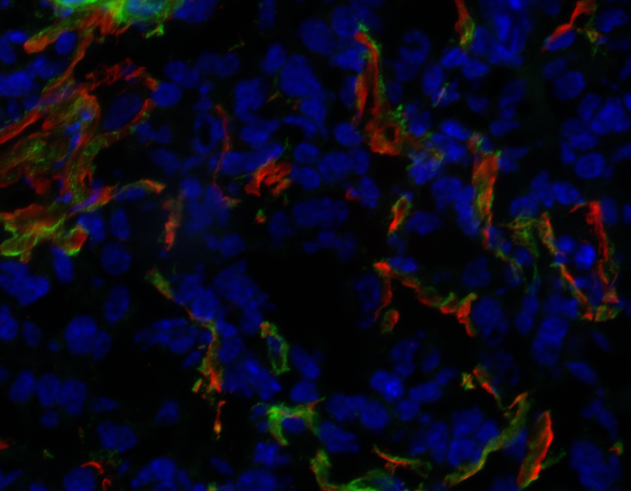

Supplement: Supplementary file 7 — Source data Fig. 5 [file 44321_2026_406_MOESM7_ESM.zip › Figure 5/Fig 5B/TNC LM OSCC.tif]

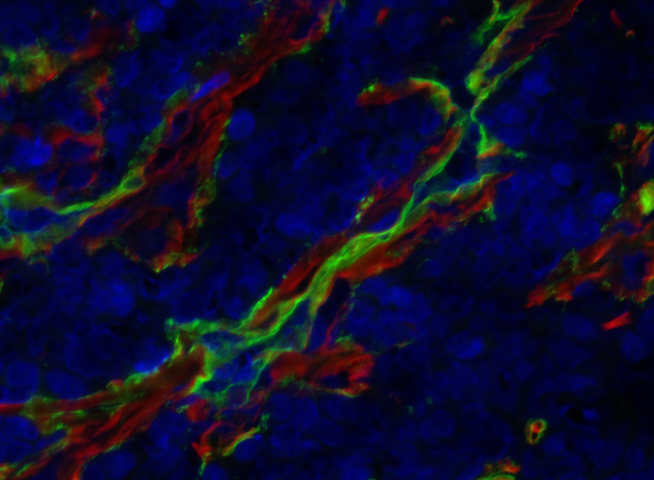

Supplement: Supplementary file 7 — Source data Fig. 5 [file 44321_2026_406_MOESM7_ESM.zip › Figure 5/Fig 5B/OSCC FRC LM TNC.tif]

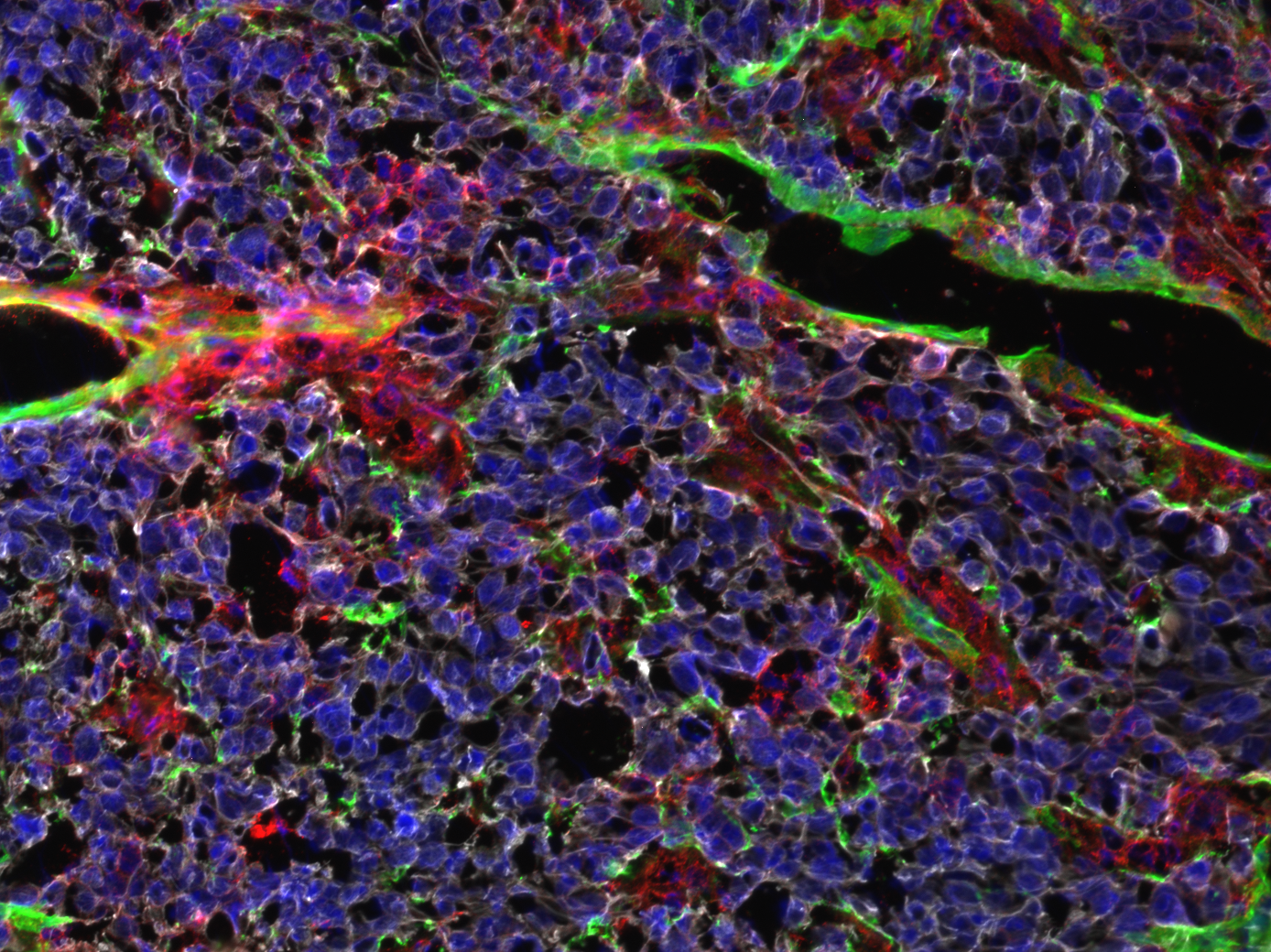

Supplement: Supplementary file 7 — Source data Fig. 5 [file 44321_2026_406_MOESM7_ESM.zip › Figure 5/Fig 5B/OSCC CD45.tif]

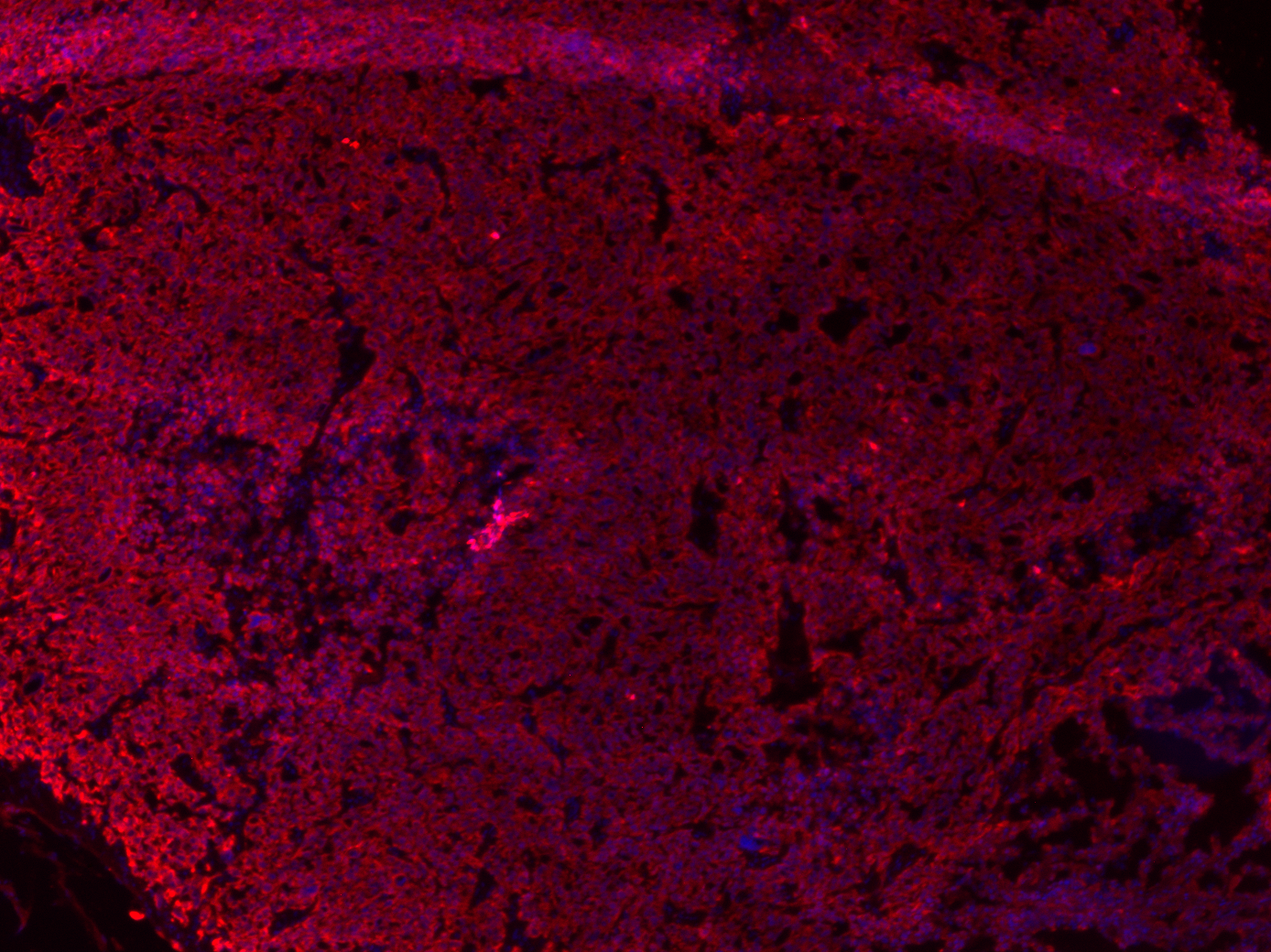

Supplement: Supplementary file 7 — Source data Fig. 5 [file 44321_2026_406_MOESM7_ESM.zip › Figure 5/Fig 5B/OSCC CK818.tif]

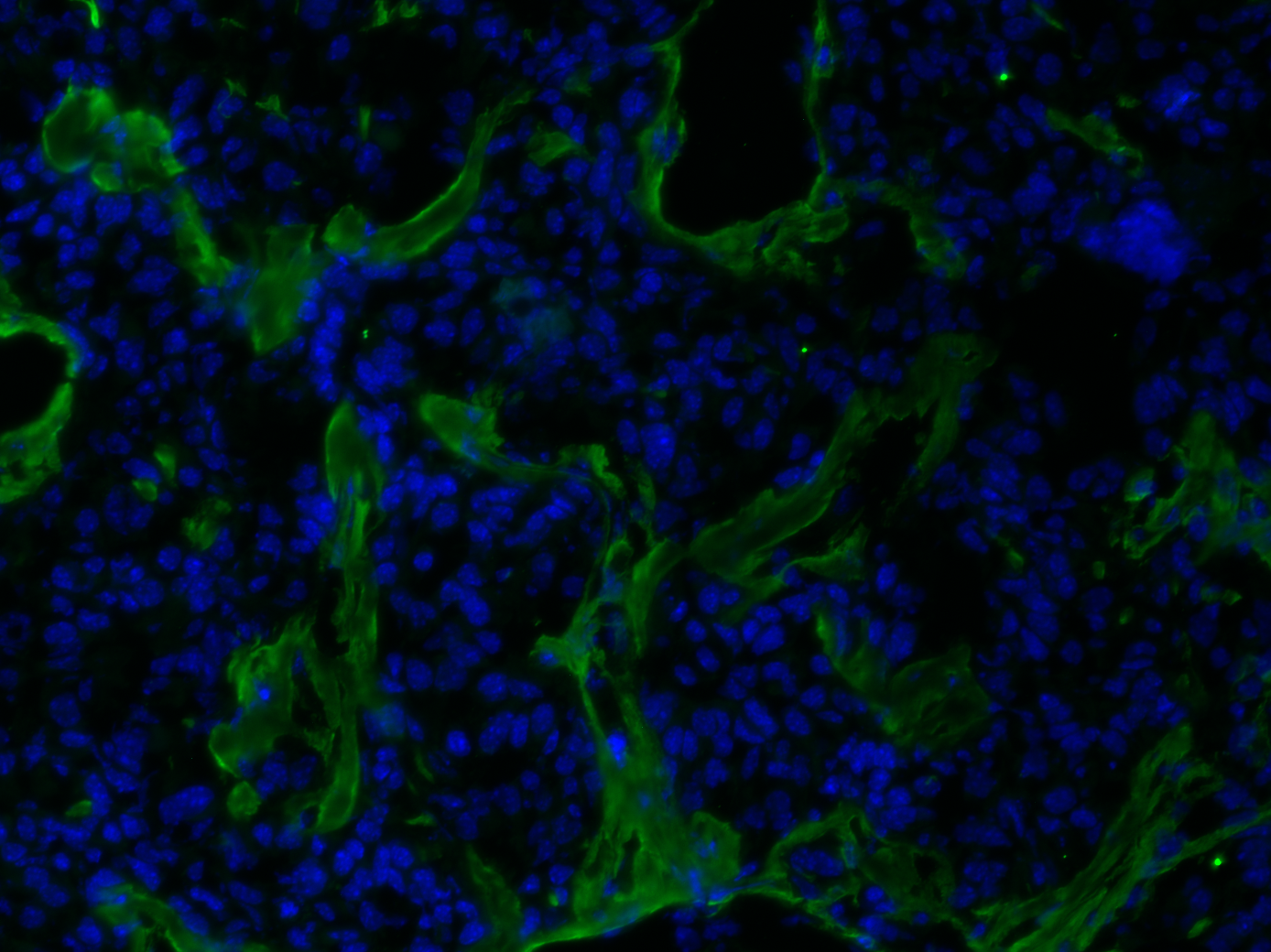

Supplement: Supplementary file 7 — Source data Fig. 5 [file 44321_2026_406_MOESM7_ESM.zip › Figure 5/Fig 5B/OSCC ERTR7.tif]

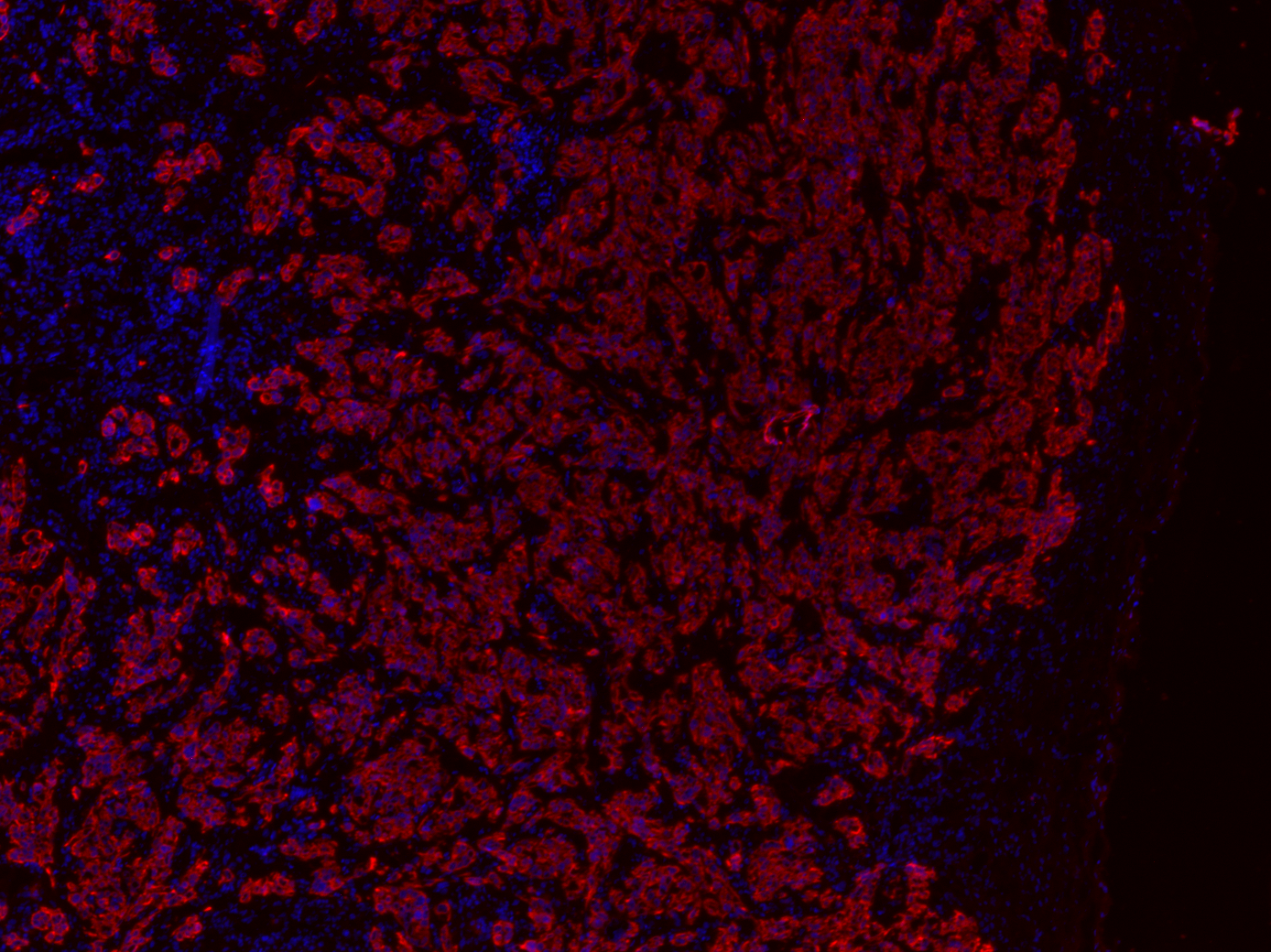

Supplement: Supplementary file 7 — Source data Fig. 5 [file 44321_2026_406_MOESM7_ESM.zip › Figure 5/Fig 5B/OSCC + FRC CK818.tif]

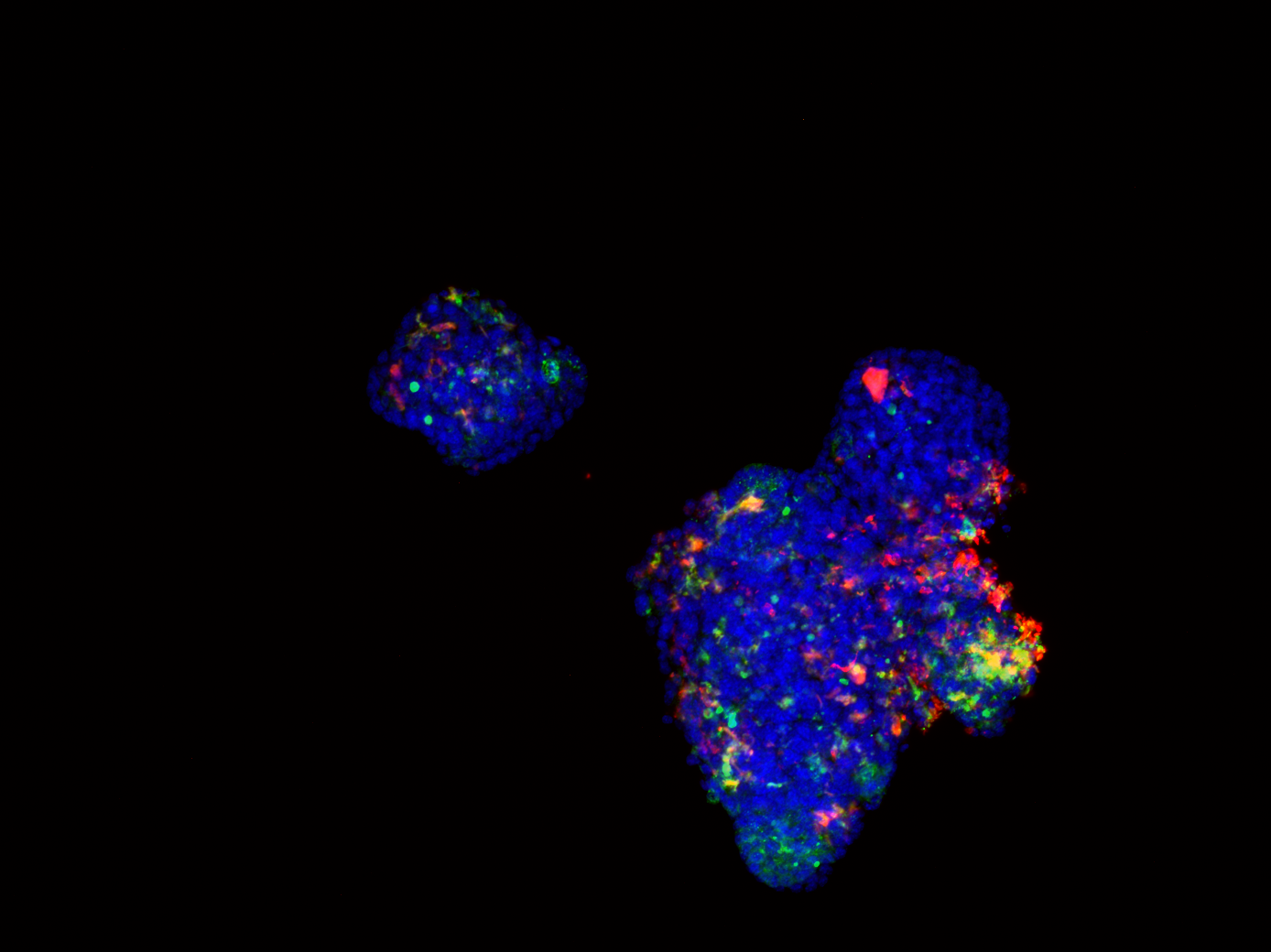

Supplement: Supplementary file 8 — Source data Fig. 6 [file 44321_2026_406_MOESM8_ESM.zip › Figure 6/Fig 6A/OSCC FRC MP5.tif]

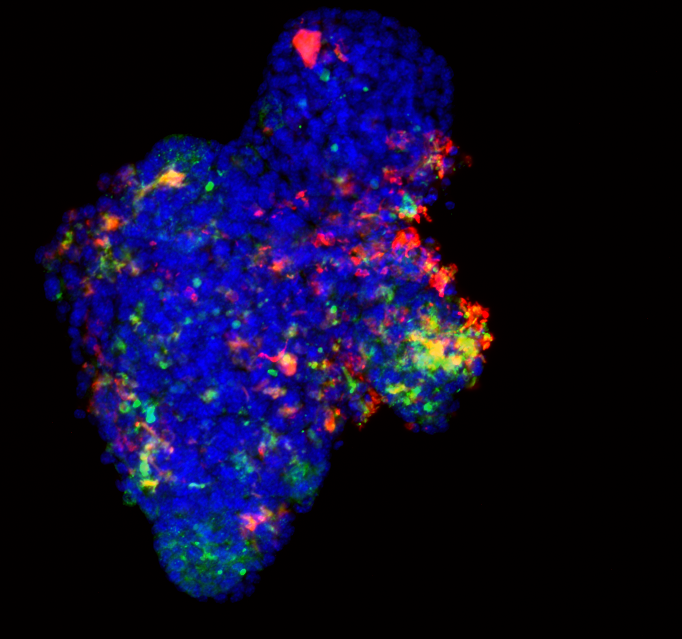

Supplement: Supplementary file 8 — Source data Fig. 6 [file 44321_2026_406_MOESM8_ESM.zip › Figure 6/Fig 6A/OSCC FRC MP5 crop.tif]

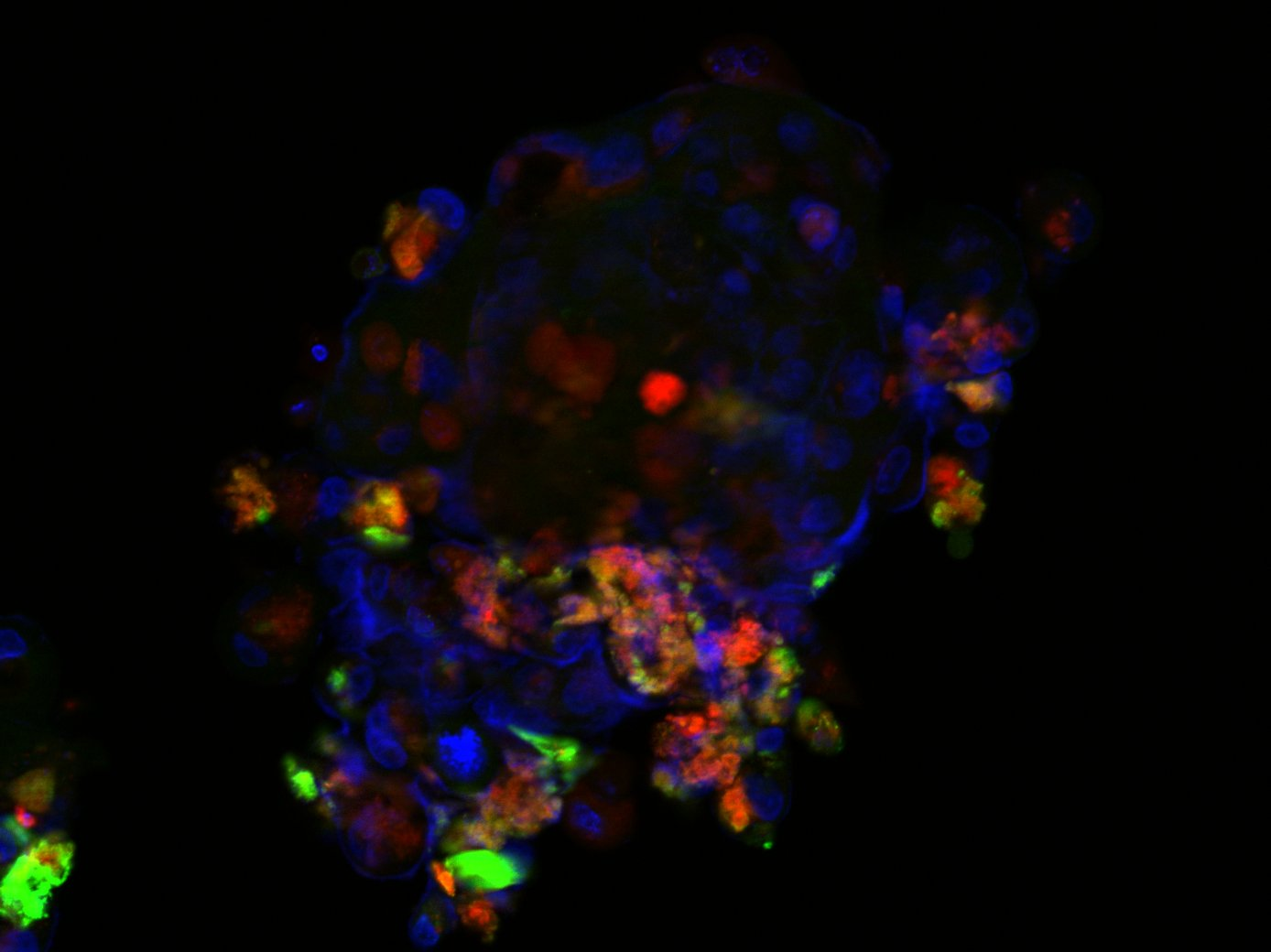

Supplement: Supplementary file 8 — Source data Fig. 6 [file 44321_2026_406_MOESM8_ESM.zip › Figure 6/Fig 6C/CAL33 TIF MP5.tif]

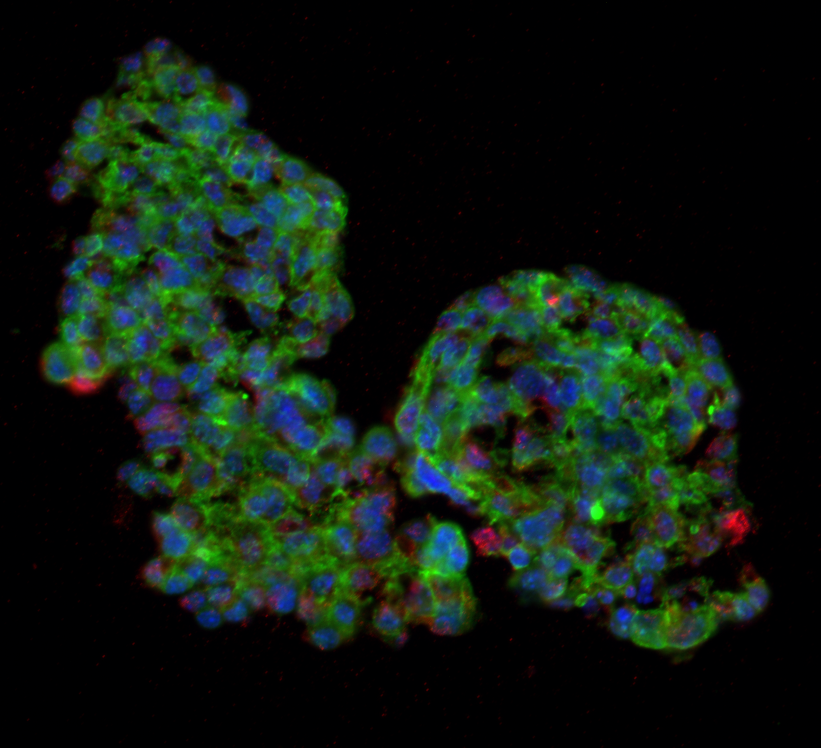

Supplement: Supplementary file 8 — Source data Fig. 6 [file 44321_2026_406_MOESM8_ESM.zip › Figure 6/Fig 6E/nircrop.tif]

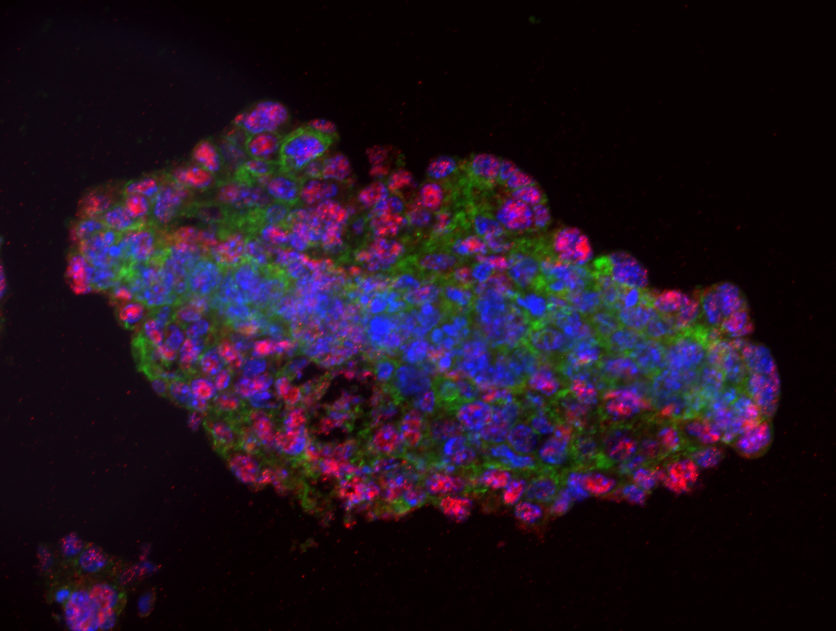

Supplement: Supplementary file 8 — Source data Fig. 6 [file 44321_2026_406_MOESM8_ESM.zip › Figure 6/Fig 6E/IRcrop.tif]

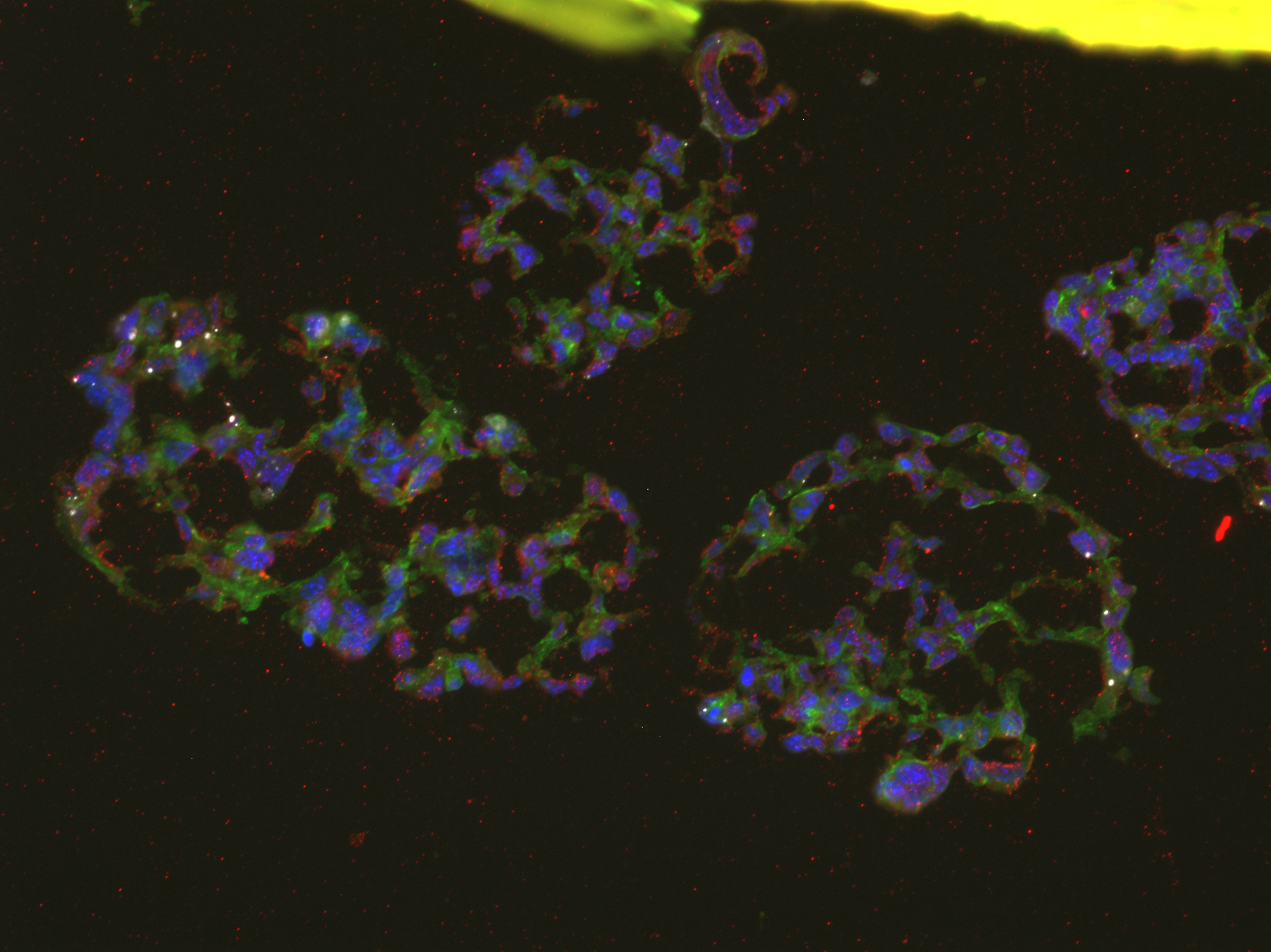

Supplement: Supplementary file 8 — Source data Fig. 6 [file 44321_2026_406_MOESM8_ESM.zip › Figure 6/Fig 6E/mp5.tif]

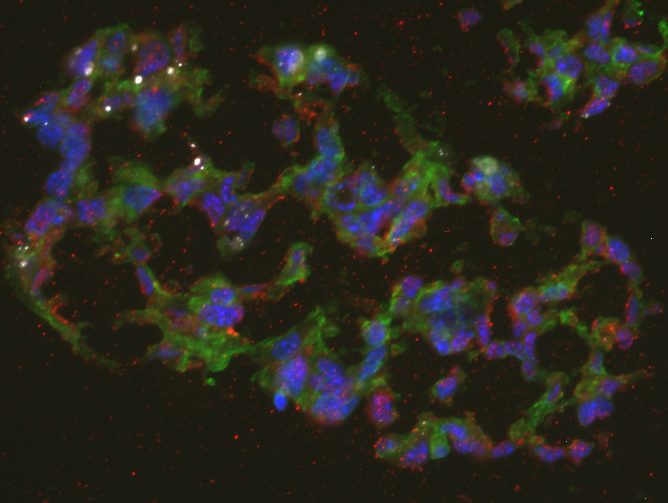

Supplement: Supplementary file 8 — Source data Fig. 6 [file 44321_2026_406_MOESM8_ESM.zip › Figure 6/Fig 6E/mp5crop.tif]

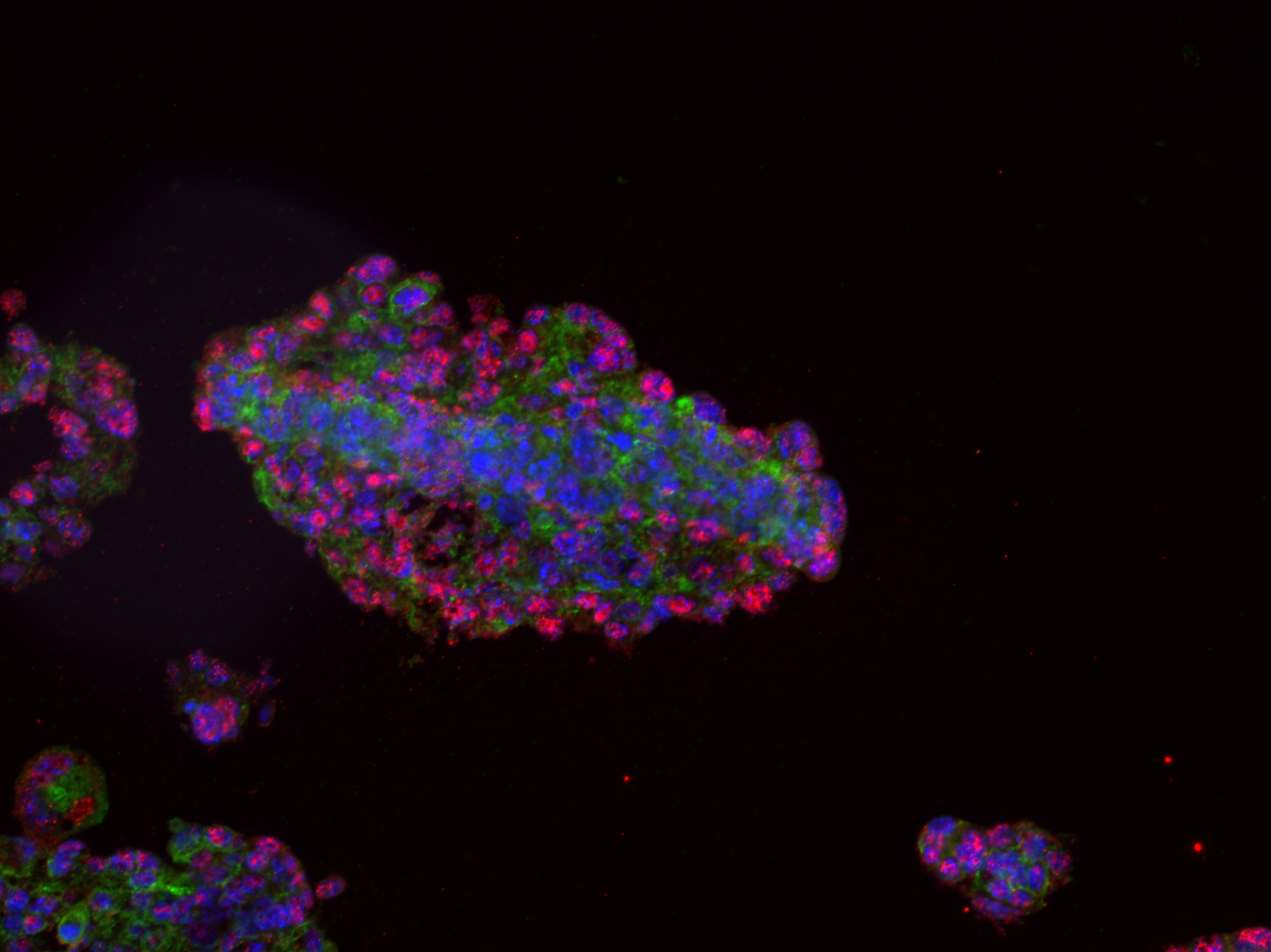

Supplement: Supplementary file 8 — Source data Fig. 6 [file 44321_2026_406_MOESM8_ESM.zip › Figure 6/Fig 6E/IR.tif]

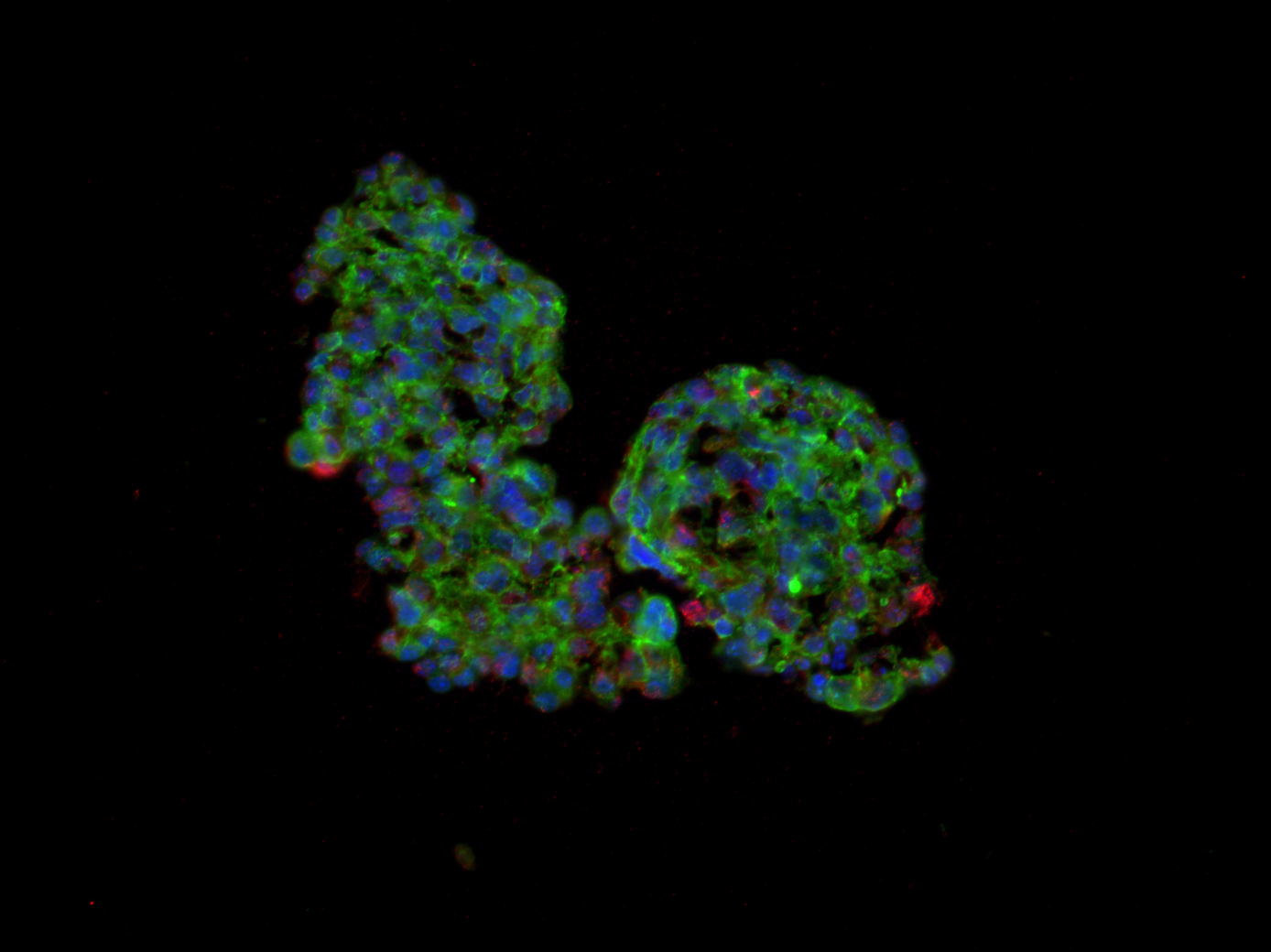

Supplement: Supplementary file 8 — Source data Fig. 6 [file 44321_2026_406_MOESM8_ESM.zip › Figure 6/Fig 6E/nir.tif]
