# Supplementary material for: Tenascin-C orchestrates radiotherapy-induced head and neck tumor regression
Source: EMBO Mol Med. 2026 Mar 31;18(5):1707–43. doi: 10.1038/s44321-026-00406-8 (PMC13179387; doi:10.1038/s44321-026-00406-8)
Supplement: Supplementary file 10 — Expanded View Figures [file 44321_2026_406_MOESM10_ESM.pdf]

# Expanded View Figures

**Figure EV1. Characterization of the 4NQO tumors and TdLNs after irradiation in WT and TNCKO mice.**

(**A**) Cartoon summarizing the 4NQO protocol combined with a single dose of 2 Gy. (**B**) Quantification of tongue tumor number in NIR and IR WT and TNCKO mice, $N = 7$–10 mice per group. (**C**, **D**) Representative IF images (of at least 25) for the indicated molecules in IR 4NQO WT and TNCKO tumors (**C**) and NIR and IR WT and TNCKO tumors (**D**). White arrows indicate the CD11c+ cells either trapped in the stroma (**C**) or reaching the tumor nests (**D**). Scale bar, 50 µm (**C**), 200 µm (**D**). (**E**–**H**) FACS analysis in TdLNs (LN) of dendritic cells (**E**), CCR7 + DC (**F**) CD86/80 + DC (**G**), and Treg (**H**) in NIR and IR 4NQO-induced WT and TNCKO tumor mice. $N = 5$ mice per group. (**I**) Gene Ontology analysis of the most highly deregulated Biological Processes in the TdLNs of WT and TNCKO mice. Mean ± SEM; Kruskal–Wallis test and Dunn post-test, *$P < 0.05$, **$P < 0.01$, ***$P < 0.005$. The exact $P$ values are listed in Appendix Table S5.

© The Author(s)

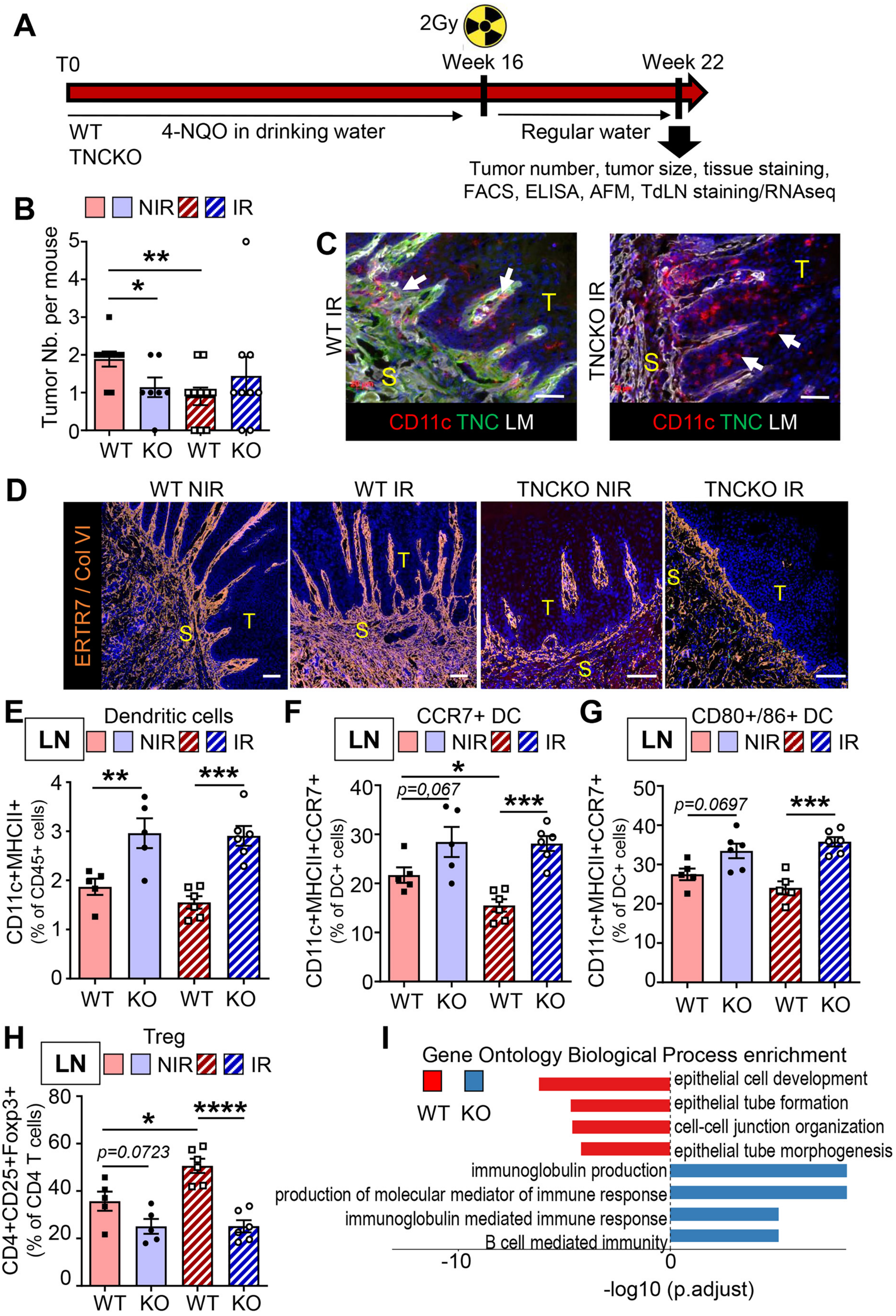
A
2Gy
T0
Week 16
Week 22
WT
TNCKO
4-NQO in drinking water
Regular water
Tumor number, tumor size, tissue staining, FACS, ELISA, AFM, TdLN staining/RNAseq
B
NIR IR
Tumor Nb. per mouse
WT KO WT KO
C
WT IR
TNCKO IR
CD11c TNC LM
D
WT NIR
WT IR
TNCKO NIR
TNCKO IR
ERTR7 / Col VI
E
Dendritic cells
LN
CD11c+MHCII+ (% of CD45+ cells)
F
CCR7+ DC
p=0,067
CD11c+MHCII+CCR7+ (% of DC+ cells)
G
CD80+/86+ DC
p=0.0697
H
Treg
p=0.0723
CD4+CD25+Foxp3+ (% of CD4 T cells)
I
Gene Ontology Biological Process enrichment
WT KO
epithelial cell development
epithelial tube formation
cell-cell junction organization
epithelial tube morphogenesis
immunoglobulin production
production of molecular mediator of immune response
immunoglobulin mediated immune response
B cell mediated immunity
-log10 (p.adjust)

 © The Author(s)

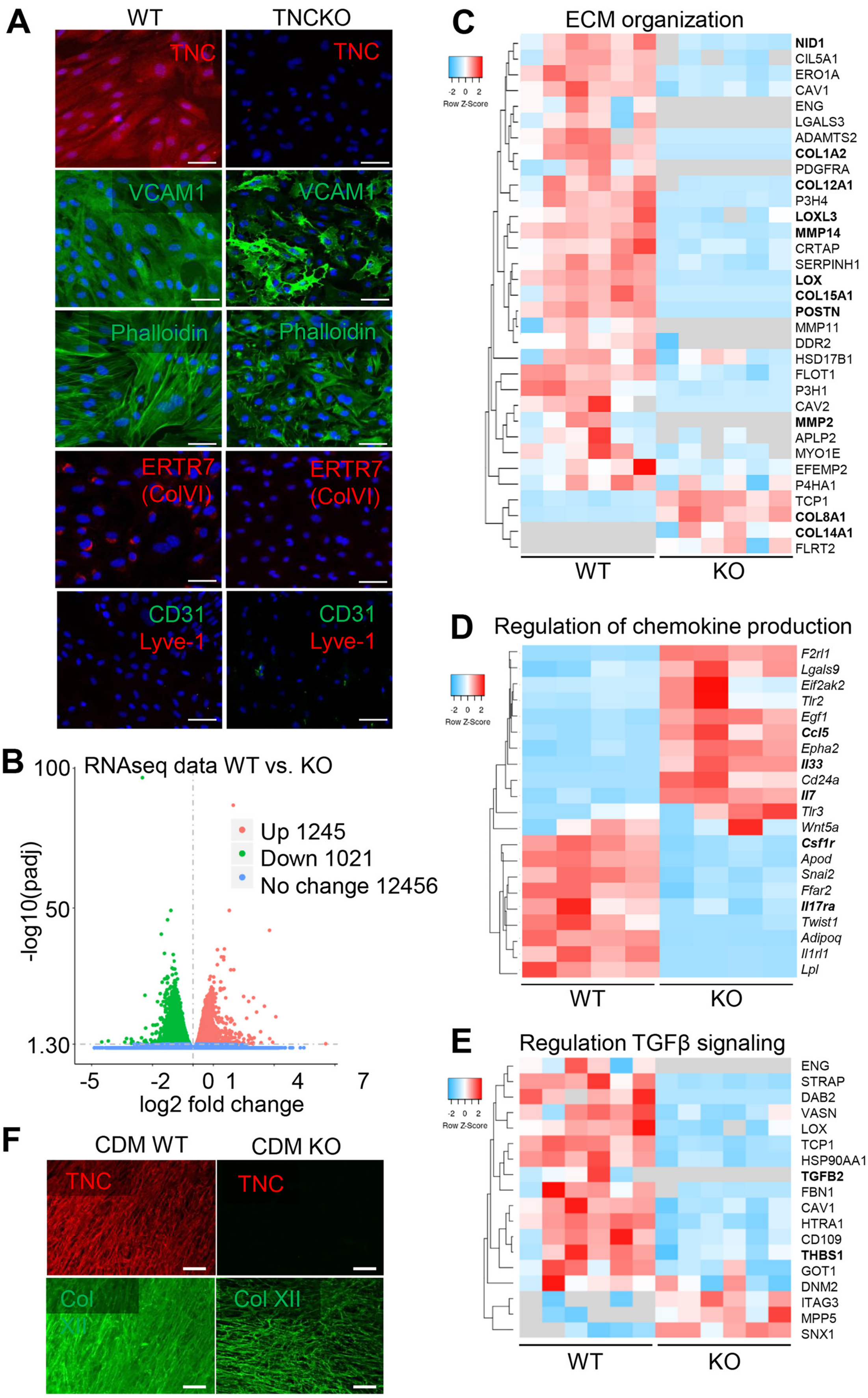
A
WT
TNCKO
TNC
VCAM1
Phalloidin
ERTR7 (ColVI)
CD31
Lyve-1
B
RNAseq data WT vs. KO
Up 1245
Down 1021
No change 12456
-log10(padj)
log2 fold change
C
ECM organization
Row Z-Score
NID1
CIL5A1
ERO1A
CAV1
ENG
LGALS3
ADAMTS2
COL1A2
PDGFRA
COL12A1
P3H4
LOXL3
MMP14
CRTAP
SERPINH1
LOX
COL15A1
POSTN
MMP11
DDR2
HSD17B1
FLOT1
P3H1
CAV2
MMP2
APLP2
MYO1E
EFEMP2
P4HA1
TCP1
COL8A1
COL14A1
FLRT2
WT
KO
D
Regulation of chemokine production
F2rl1
Lgals9
Eif2ak2
Tlr2
Egf1
Ccl5
Epha2
Il33
Cd24a
Il7
Tlr3
Wnt5a
Csf1r
Apod
Snai2
Ffar2
Il17ra
Twist1
Adipoq
Il1rl1
Lpl
E
Regulation TGFβ signaling
ENG
STRAP
DAB2
VASN
LOX
TCP1
HSP90AA1
TGFB2
FBN1
CAV1
HTRA1
CD109
THBS1
GOT1
DNM2
ITAG3
MPP5
SNX1
F
CDM WT
CDM KO
TNC
Col XII

© The Author(s)

◀ **Figure EV2. TNC expression plays a pivotal role in determining the FRC identity.**

(**A**) Representative IF images for TNC, VCAM-1, polymerized actin (phalloidin staining), collagen VI (ERTR7), CD31, and Lyve-1 in FRCs isolated from naïve lymph nodes of WT or TNCKO mice. For VCAM-1, the same microscopic field is shown that has been co-stained for GP38 in Fig. 2A. Scale bar, 50 µm. (**B**) Volcano plot of deregulated genes (DEGs) obtained after RNA sequencing of FRC WT and TNCKO cells. Volcano plot showing the fold change and the adjusted *P* value for the 14,722 genes expressed with overexpression (red dots) and downregulation (green dots) in FRC WT compared to TNCKO cells. (**C–E**) Proteomics data analysis represented as heatmaps for the most deregulated proteins that belong to ECM organization (**C**), Regulation of chemokine production (**D**), and regulation of the TGFβ signaling (**E**). Bold text indicates proteins with established roles in the respective categories that are discussed in the text. (**F**) Representative IF staining images of Col XII and TNC expression in the CDM obtained after 10 days of FRC WT and TNCKO cell cultures. Scale bar, 200 µm.

 © The Author(s)

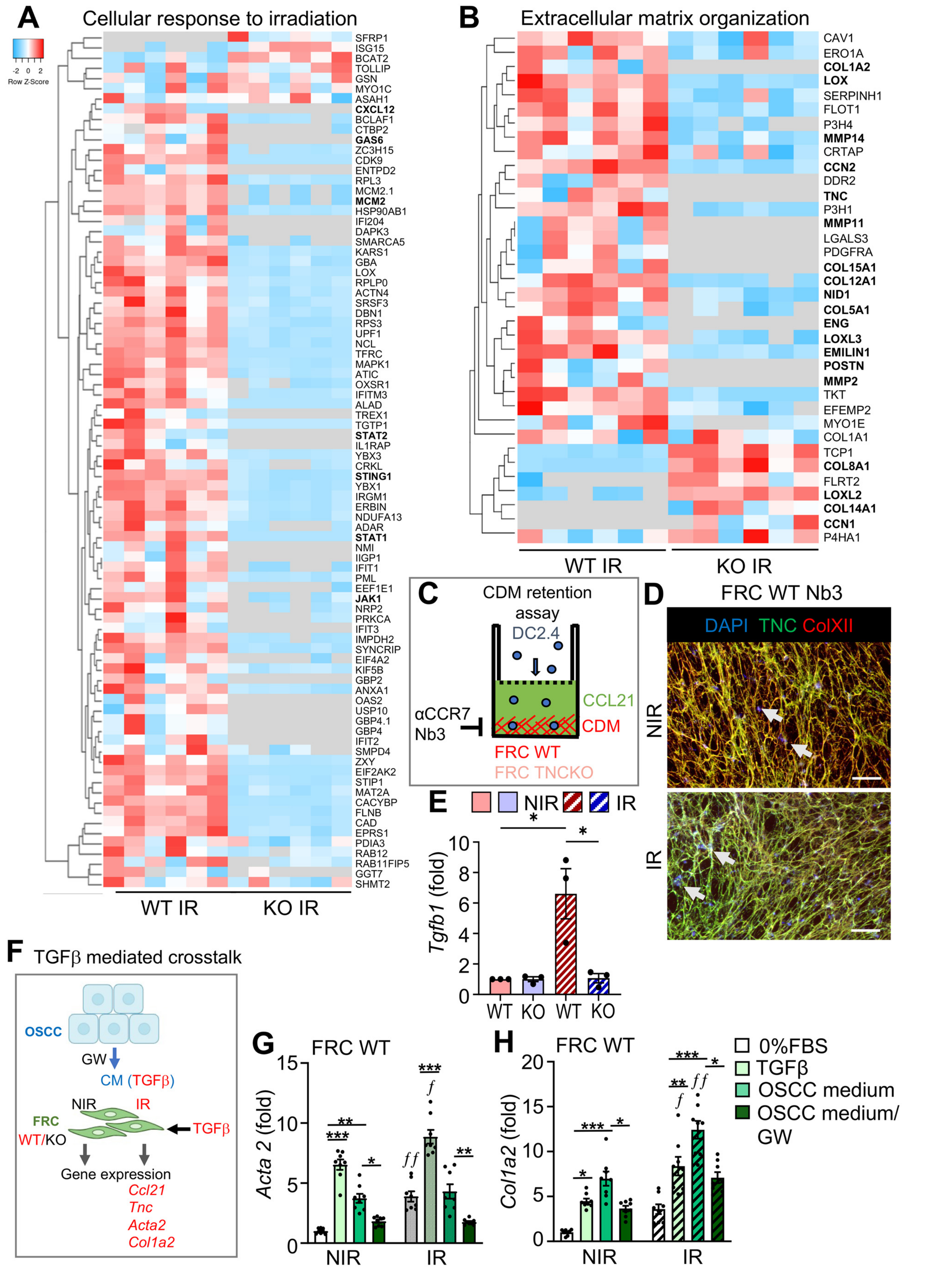
A
Cellular response to irradiation
Row Z-Score
WT IR
KO IR
B
Extracellular matrix organization
WT IR
KO IR
C
CDM retention assay
DC2.4
CCL21
CDM
αCCR7 Nb3
FRC WT
FRC TNCKO
D
FRC WT Nb3
DAPI TNC ColXII
NIR
IR
E
NIR
IR
Tgfb1 (fold)
WT KO WT KO
F
TGFβ mediated crosstalk
OSCC
GW
CM (TGFβ)
NIR
IR
FRC WT/KO
TGFβ
Gene expression
Ccl21
Tnc
Acta2
Col1a2
G
FRC WT
Acta 2 (fold)
NIR
IR
H
FRC WT
Col1a2 (fold)
NIR
IR
0%FBS
TGFβ
OSCC medium
OSCC medium/ GW

**Figure EV3. The FRC cell response to irradiation is determined by TNC expression.**

(**A**, **B**) Heatmap representation of the indicated proteome categories of irradiated FRCs (WT vs. TNCKO), represented as heatmaps for deregulated proteins involved in the cellular response to irradiation (**A**) and ECM organization (**B**). Bold text indicates proteins with established roles in the respective categories and/or that are discussed in the text. (**C**) Schematic representation of the DC retention assay with CCL21 (200 ng/ml) as chemoattractant and anti-CCR7 and Nb3 as TNC inhibitors. Note the less binding of cells to the CDM from TNCKO FRCs (light red) and upon inhibition of TNC. (**D**) Representative IF images of DC2.4 adherent on the CDM after the chemoretention assay in the presence of Nb3. Staining is as indicated. Arrows indicate the nuclei. Scale bar, 200 µm. (**E**) Gene expression (qRTPCR) of *Tgfb1* in WT and TNCKO FRCs, NIR or IR. $N = 3$. Mean ± SD, two-way ANOVA with $^*P < 0.05$. (**F**) Schematic representation of the experimental setup to assess a TGFβ-mediated cellular crosstalk between OSCC13 cells (OSCC) providing Conditioned Medium (CM) that was added to FRCs (WT, KO, NIR, IR) in the presence or absence of GW788388 (GW) in comparison to TGFβ (10 ng/ml). Gene expression of the indicated molecules was assessed by qRTPCR. Note a significant stimulating effect of the CM of the OSCC13 cells on irradiated WT FRCs in a TGFβ signaling depenndent manner (red). (**G**, **H**) Gene expression as determined by qRTPCR in FRC WT for *Acta2* and *Col1a2*. $N = 6$–9 per condition. Error bars represent mean ± SEM, two-way ANOVA with $^*P < 0.05$, $^{**}P < 0.01$, $^{***}P < 0.005$. The exact *P* values are listed in Appendix Table S5.

 © The Author(s)

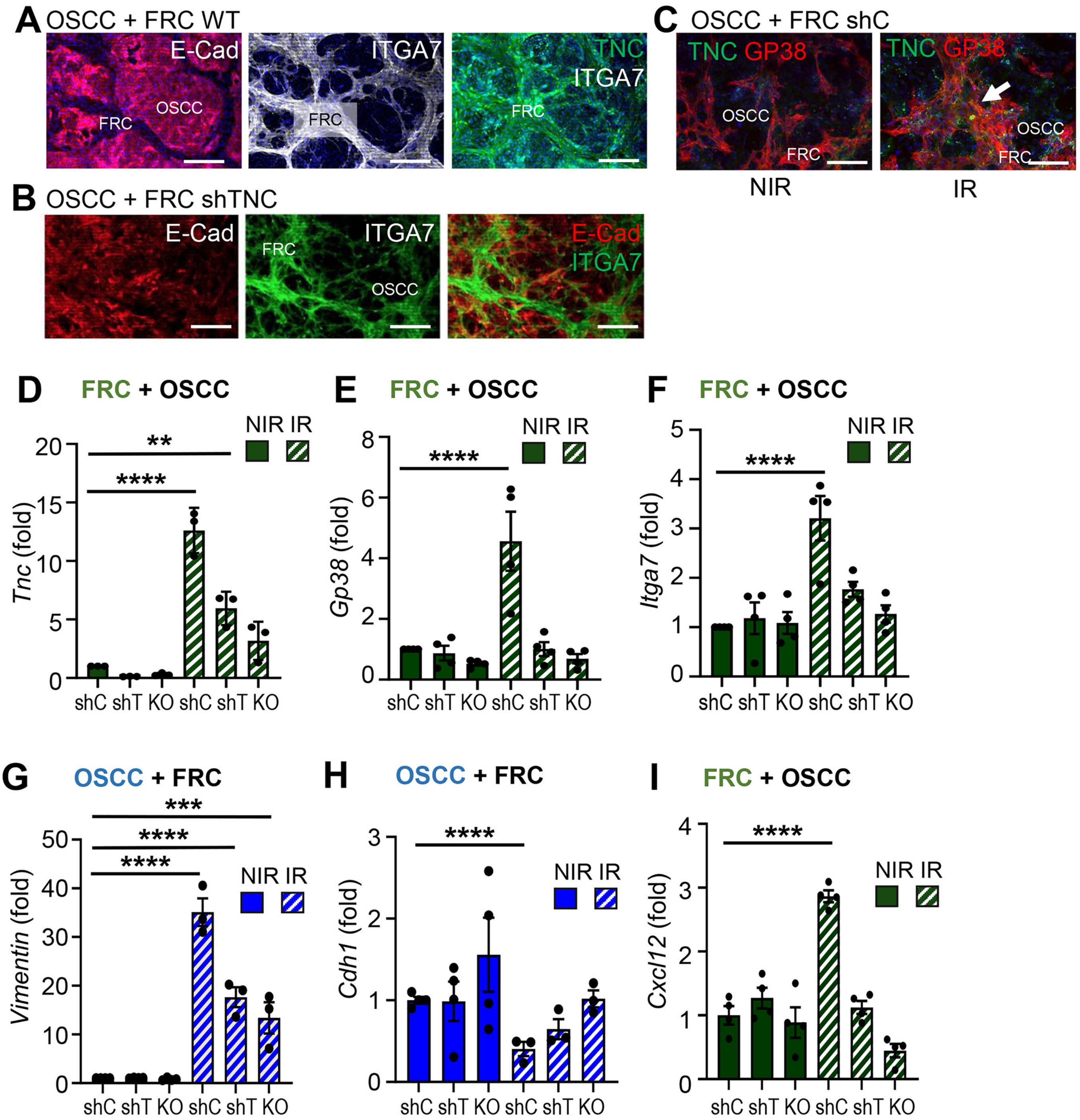


**Figure EV4. TNC impact on the OSCC-FRC crosstalk upon irradiation.**

(**A–C**) Immunofluorescence images after staining for the indicated molecules in cocultures of OSCC13 (OSCC) with FRCs at a 2.1 ratio for 4 days. FRCs and OSCC are labeled. Arrow points at increased GP38 and TNC levels in the IR condition (**C**). Scale bars, 200 µm. (**D–I**) Gene expression (qRTPCR) in FRCs (WT, shTNC (shT), TNCKO (KO)) (**D–F**, **I**) or OSCC13 (OSCC) (**G**, **H**) upon coculture, IR or NIR, and isolation by MACS for the indicated molecules. $N = 3$ experiments; Error bars represent mean ± SEM; ordinary one-way ANOVA test, ***$P < 0.005$. ****$P < 0.0001$. The exact $P$ values are listed in Appendix Table S5.

© The Author(s)

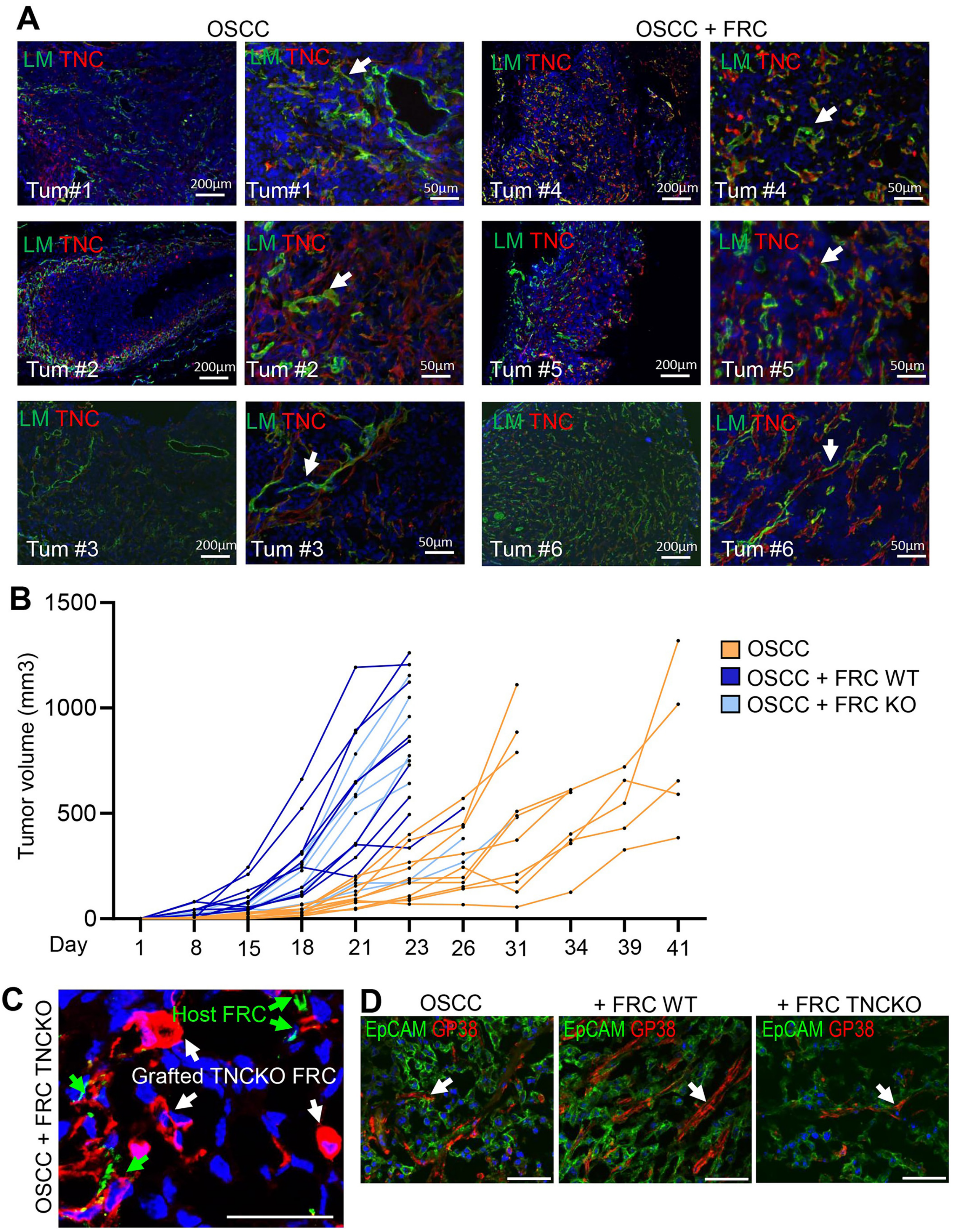
A
OSCC
OSCC + FRC
LM TNC
Tum#1
Tum #2
Tum #3
Tum #4
Tum #5
Tum #6
200µm
50µm
B
Tumor volume (mm3)
1500
1000
500
0
Day
1 8 15 18 21 23 26 31 34 39 41
OSCC
OSCC + FRC WT
OSCC + FRC KO
C
OSCC + FRC TNCKO
Host FRC
Grafted TNCKO FRC
D
OSCC
+ FRC WT
+ FRC TNCKO
EpCAM GP38

 © The Author(s)

**Figure EV5. Characterization of neck coengrafted tumors in C57Bl6 mice.**

(**A**) Representative IF images of neck tumors displayed in Fig. 5A, obtained after OSCC grafting ($3 \times 10^6$ cells) or OSCC/FRC (5:1 ratio) co-graftments in WT mice for the indicated markers. The LM/TNC costained panel of the OSCC/FRC tumor #6 represents an uncropped version of the image shown in Fig. 5B. Scale bar, 200 µm. (**B**) Monitoring of individual tumor growth (volume) of the engrafted OSCC13 (OSCC) cells alone ($3 \times 10^6$ cells) (orange) or combined with WT FRC (dark blue), or FRC TNCKO cells (light blue), in a 5:1 ratio, into the neck of WT mice during the indicated time frame (up to 41 days). (**C**) Enlarged image showing FRCs (GP38 $+$ ) in a OSCC/TNCKO FRC-engrafted tumor where the engrafted FRCs are negative for TNC while the host FRCs are positive as indicated with the colored arrows. Scale bar, 50 µm. (**D**) Representative immunofluorescence images for EpCAM and GP38 in the tumors displayed in Fig. 5D derived from engraftments of the OSCC cells alone ($3 \times 10^6$ cells) (orange) or in combination with WT FRC (dark blue), or FRC TNCKO cells (light blue), in a 5:1 ratio, into the neck of WT mice during the indicated time frame (up to 41 days). Scale bars, 50 µm. Arrow points at FRCs.

© The Author(s)

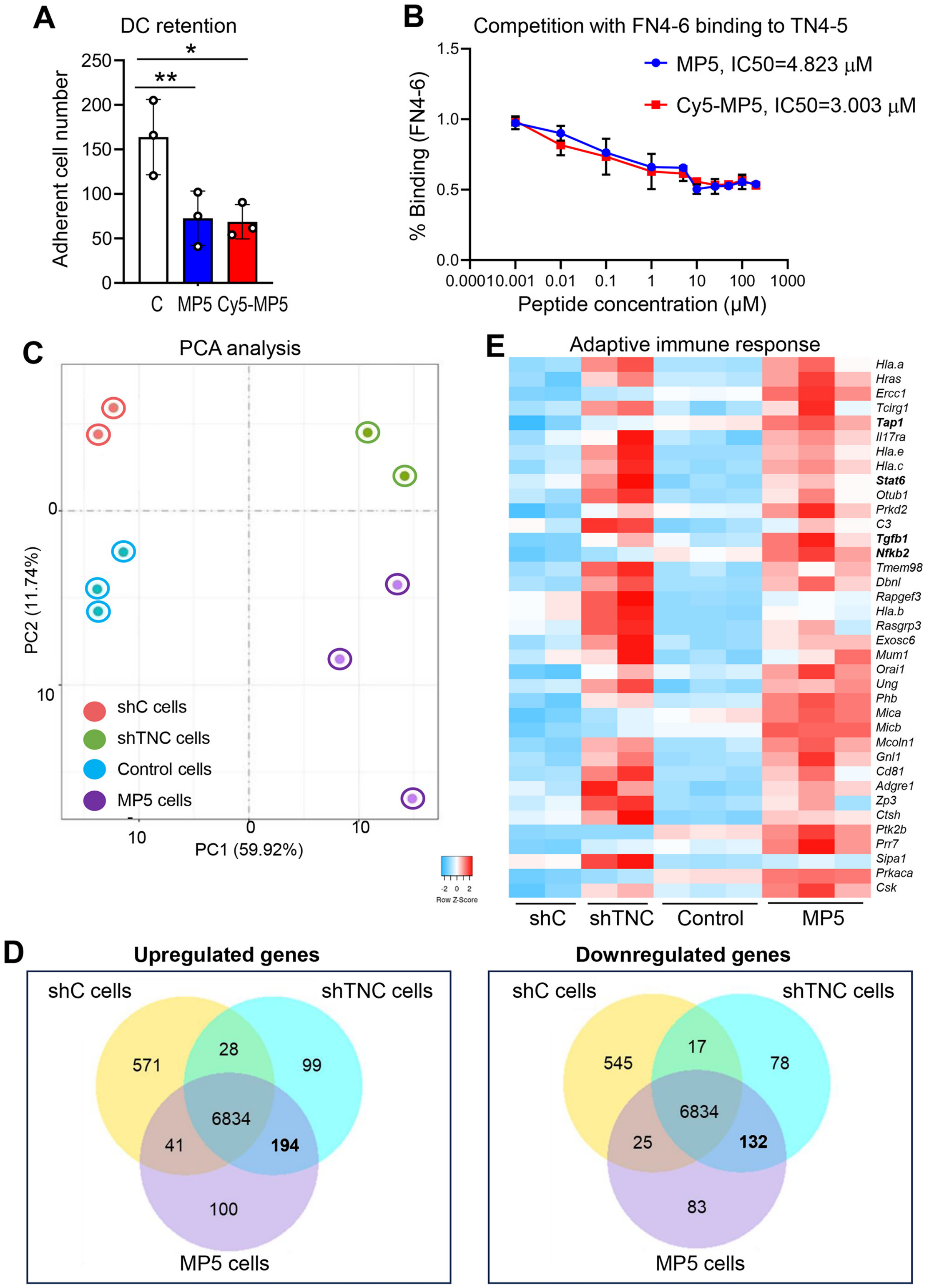
A
DC retention
Adherent cell number
*
**
C
MP5
Cy5-MP5
B
Competition with FN4-6 binding to TN4-5
% Binding (FN4-6)
MP5, IC50=4.823 μM
Cy5-MP5, IC50=3.003 μM
Peptide concentration (μM)
C
PCA analysis
PC2 (11.74%)
PC1 (59.92%)
shC cells
shTNC cells
Control cells
MP5 cells
E
Adaptive immune response
Hla.a
Hras
Ercc1
Tcirg1
Tap1
Il17ra
Hla.e
Hla.c
Stat6
Otub1
Prkd2
C3
Tgfb1
Nfkb2
Tmem98
Dbnl
Rapgef3
Hla.b
Rasgrp3
Exosc6
Mum1
Orai1
Ung
Phb
Mica
Micb
Mcoln1
Gnl1
Cd81
Adgre1
Zp3
Ctsh
Ptk2b
Prr7
Sipa1
Prkaca
Csk
Row Z-Score
shC
shTNC
Control
MP5
D
Upregulated genes
shC cells
shTNC cells
571
28
99
6834
41
194
100
MP5 cells
Downregulated genes
shC cells
shTNC cells
545
17
78
6834
25
132
83
MP5 cells

 © The Author(s)

**Figure EV6. Targeting TNC with MAREMO peptide MP5 reduces tumor cell proliferation and plasticity upon IR.**

(**A**) Chemoretention assay with DC2.4 cells measuring adhesion on the TNC-coated lower surface of the insert with 200 ng/mL CCL21 as chemoattractant. Note, both peptides (25 nM) cause release of the cells from TNC. $N = 3$ experiments; Mean ± SD; Ordinary one-way ANOVA test, $^*P < 0.05$. (**B**) Competitive ELISA measuring binding of FN4-6 to surface-adsorbed TN4-5 which is blocked in a dose-dependent manner by both MP5 and Cy5-MP5. Dose–response inhibition curves were fitted using nonlinear regression with a four-parameter logistic model. $IC_{50}$ values were compared using an extra sum-of-squares F test. $N = 3$ experiments, Mean ± SD. (**C**) PCA analysis of the RNA sequencing data from NT193 breast cancer cells (shC, shTNC, Control (PBS-treated) and MP5-treated), indicating a similar distribution of the shTNC and MP5 samples. (**D**) RNA sequencing analysis of the upregulated and downregulated genes in NT193 breast cancer cells shC, shTNC or MP5-treated. In the centre, the commonly expressed genes between all categories are shown. 194 genes are commonly upregulated in the MP5 and shTNC conditions compared to the shC condition, and 132 genes are commonly downregulated in the MP5 and shTNC conditions compared to the shC condition. (**E**) Heatmap representation of gene expression data (derived from RNA sequencing) linked to adaptive immune response between groups ($P < 0.05$). The exact $P$ values are listed in Appendix Table S5.

© The Author(s)
